# Spatiotemporal transcriptome and scRNA sequencing analysis reveals that *IKFZ1*‐mediated microglia underlies a therapy for intracerebral hemorrhage

**DOI:** 10.1002/ctm2.70127

**Published:** 2024-12-10

**Authors:** Wenqiao Qiu, Jie Tian, Gao Mou, Lili Guo, Tao Xu, Wei Liu, Jianwei Zhu, Yi Zhang, Xiaolin Hou, Yao Xie, Huan Xiong, Xinda Li, Yangyang Wang, Mingjun Gao, Anguo Wu, Longyi Chen, Jie Mei, Lulin Huang, Ruxiang Xu

**Affiliations:** ^1^ Department of Neurosurgery Sichuan Provincial People's Hospital School of Medicine University of Electronic Science and Technology of China Chengdu China; ^2^ Department of Neurosurgery Chinese PLA General Hospital Beijing China; ^3^ Biomanufacturing and Rapid Forming Technology Key Laboratory of Beijing Department of Mechanical Engineering Tsinghua University Beijing People's Republic of China; ^4^ Department of Obstetrics & Gynecology, Sichuan Provincial People's Hospital University of Electronic Science and Technology of China Chengdu China; ^5^ Sichuan Key Medical Laboratory of New Drug Discovery and Drugability Evaluation Luzhou Key Laboratory of Activity Screening and Druggability Evaluation for Chinese Materia Medica School of Preclinical Medicine Key Laboratory of Medical Electrophysiology of Ministry of Education; School of Pharmacy Southwest Medical University Luzhou China; ^6^ Sichuan Provincial Key Laboratory for Human Disease Gene Study and the Center for Medical Genetics Department of Laboratory Medicine Sichuan Academy of Medical Sciences and Sichuan Provincial People's Hospital University of Electronic Science and Technology of China Chengdu China; ^7^ Research Unit for Blindness Prevention of Chinese Academy of Medical Sciences (2019RU026) Sichuan Academy of Medical Sciences & Sichuan Provincial People's Hospital Chengdu China

1

Dear Editor,

After intracerebral hemorrhage (ICH), significant inflammatory response and neuronal damage are evident.^1^ Inflammatory factors induce neuronal apoptosis, which is the main cause of neuronal dysfunction.^2^ Although exosomes (Exo) are being studied as a treatment for neuronal dysfunction, their crucial mechanisms have remained unknown.^3^, ^4^

This study investigated the essential mechanisms by which Exo alleviates neuronal dysfunction post‐ICH. First, we confirmed that post‐Exo, the neuronal dysfunction and brain inflammation of ICH‐rats were significantly improved (Figure S1). Subsequently, brain tissues were collected (Figure S2A–C) for single‐cell sequencing (scRNA‐Seq) and spatial transcriptome (ST). Following the annotation of ST (Figure 1A,B), pseudo‐temporal analysis of the peri‐hematoma area (Figure 1C,D) revealed a decrease in the expression of pyroptosis‐related genes (V) post‐Exo. Subsequent KEGG analysis of different time‐points ST (Figure 1E; Table S1) indicated that post‐Exo, the enrichment scores of immune‐activations and apoptosis‐related pathways decreased, while those of synaptic plasticity‐related pathways increased.

**FIGURE 1:**
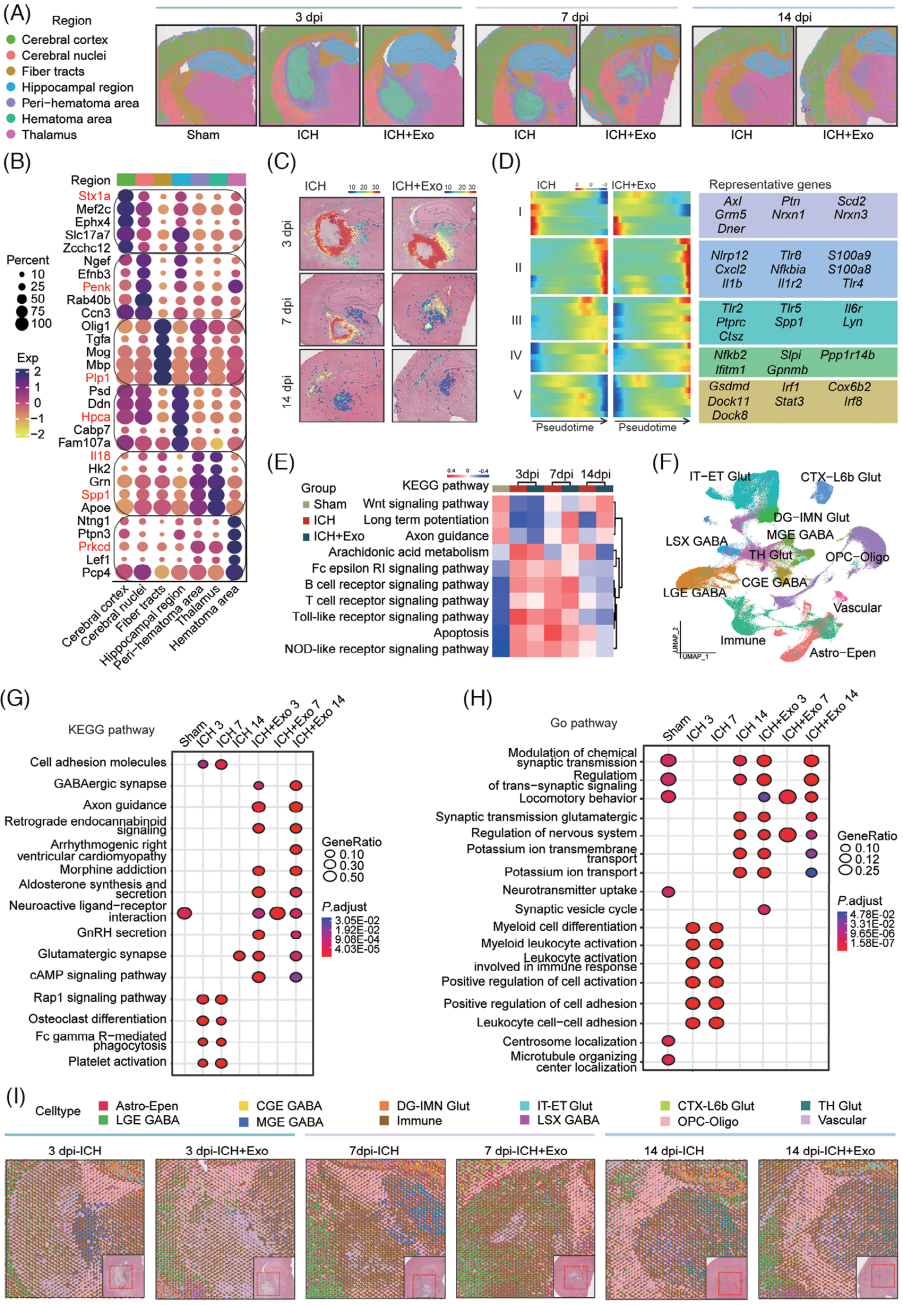
(A) Deconvolved the ST dataset to infer activity maps after hematoxylin and eosin (H&E) staining imaging and annotated the representative ST with tissue features and marker genes from the Allen Brain Atlas. Defined seven main regions in the ICH‐rat brain: cerebral cortex, cerebral nuclei, fibre tracts, hippocampal region, per‐hematoma area, hematoma area, and thalamus. (B) A bubble chart illustrating the characteristic markers of these brain regions. (C) Monocle‐2 analysis depicted the developmental trajectory of cells in the peri‐hematoma area over time (3–14 dpi) in ICH and ICH+Exo groups. (D) Heatmaps represent the dynamic expression of genes that exhibit significantly different patterns between the ICH and ICH+Exo groups, organized into five clusters through hierarchical clustering. (E) Comparative analysis of KEGG pathway activities across different ST samples was conducted using Gene Set Variation Analysis (GSVA), with pathway scores normalized and significance determined at an adjusted *p*‐value < .05. (F) Visualization of scRNA‐seq data was performed via Uniform Manifold Approximation and Projection (UMAP), identifying a total of 278 394 cells categorized into 12 distinct cell types: Astro‐Epen (astrocytes and ependymal cells), LGE GABA (GABAergic cells from the lateral ganglionic eminence), CGE GABA (GABAergic cells from the caudal ganglionic eminence), MGE GABA (GABAergic cells from the medial ganglionic eminence), DG‐IMN Glut (glutamatergic immature neurons from the dentate gyrus), immune (immune cells), IT‐ET Glu (glutamatergic cells from intratelencephalic and extratelencephalic regions), LSX GABA (GABAergic cells from the lateral septal complex), CTX‐L6b Glut (glutamatergic cells from the cerebral cortex layer 6b), OPC‐Oligo (oligodendrocyte precursor cells and oligodendrocytes), TH Glut (glutamatergic cells from the thalamus), and vascular. (G) Key KEGG pathway terms associated with significantly enriched genes identified by GSEA across different groups. (H) Representative GO biological process pathway terms linked to significantly enriched genes were determined by GSEA across various groups. (I) A comprehensive analysis of gene sets derived from scRNA‐seq data was performed, applying robust‐cell‐type decomposition (RCTD) to deconvolve ST data (3–14 dpi). The figure illustrates the distribution of cell types at ST monitoring points in the perhematoma area and hematoma area (displayed in a pie chart), with a full panoramic image and corresponding location depicted in the lower‐left corner.

The scRNA‐Seq results identified 12 cell types (Figure 1F; Table S2). KEGG and GO analysis (Figure 1G,H; Tables S3–S4) showed that Exo reduces immune activation and boosts neural protection pathways. Immune changed most significantly post‐Exo (Figure S3C,D). Moreover, the integration of ST and scRNA‐Seq indicated a notable clustering of immune in the peri‐hematoma area (Figure 1I).

Subtype analysis of scRNA‐Seq identified 29 subtypes (Figure S4A, Table S5). Immune is categorized as MDM, DC, microglia, and neutrophils (Figure S4B,C). Microglia were categorized as M_Ptn,_ M_Ikzf1_, M_Resident_, and M_few_ (Figure S4D–E). KEGG analysis showed significant enrichment of neurodevelopment pathways in the M_Resident_ and M_Ptn_, with immune regulation pathways enriched in the M_Ikzf1_ (Figure S4F, Table S6). M_Ikzf1_ and M_Ptn_ predominated in the ICH and ICH+Exo groups, respectively (Figure 2A,B). Subpopulations of microglia in the brain were analyzed using immunofluorescence and flow cytometry (Figure 2C–E) to assess the impact of Exo. An increased co‐expression of Ptn‐Iba1&CD11b post‐Exo, whereas a heightened co‐expression of Ikzf1&Iba1&CD11b was observed in the ICH group. To elucidate the primary driving genes in microglia, we employed SCENIC analysis (Figure 2F) and constructed a gene‐regulatory network specific to the MIkzf1 based on the top five transcripts (Figure S5A, Table S7). Through GO analysis of these transcripts (Figure S5B–E), Ikzf1 and its target genes exhibit a notable correlation with immune cell differentiation and activation (Figure S5F, Table S8). These findings emphasize the pivotal role of Ikzf1 in governing the immune response facilitated by microglia.

**FIGURE 2:**
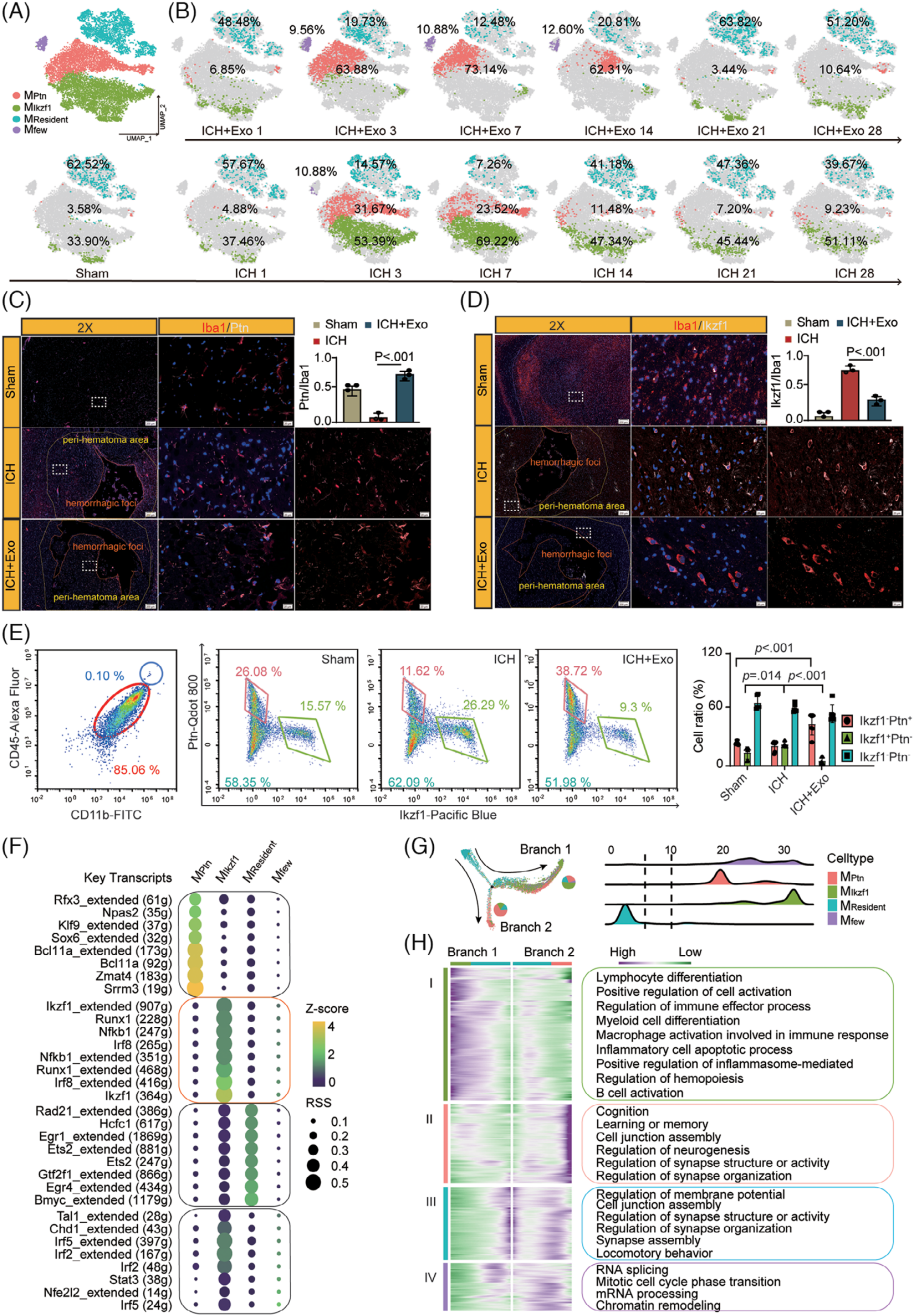
(A) T‐Distributed Stochastic Neighbor Embedding (TSNE) plot showing four microglia subclasses. (B) TSNE plots indicate temporal changes and proportions of microglia subclasses. (C) Co‐immunostaining of Iba1&Ptn in the peri‐hematoma area and quantitative analysis in 3 dpi, *n* = 3 rats per group. (D) Co‐immunostaining of Iba1&Ikzf1 in the peri‐hematoma area and quantitative analysis in 3 dpi, *n* = 3 rats per group. (E) Flow cytometry analysis was conducted to assess the proportion of microglia subclass transitions in rat brains at 3 dpi, *n* ≥ 6 rats per group. Data are presented as mean ± SD. The normal distribution was determined with a Shapiro–Wilk test, yielding a *p*‐value > .05. (F) Single‐cell regulatory network inference and clustering (SCENIC) regulon analysis depicting key regulons across microglia subclasses. (G) The developmental trajectory of microglia subclasses was inferred using Monocle‐2, with colouring based on subclass classification. Pie charts illustrate the distribution of cell subclasses across various branches. (H) A heatmap displays distinct gene expression patterns along two developmental trajectories (branch 1 and branch 2) originating from the M_Resident_ subclass. Genes are categorized into four clusters through hierarchical clustering, with GSEA‐enriched biological process pathways shown on the right.

Pseudo‐time analysis indicates that the developmental trajectories of M_Ikzf1_ and M_Ptn_ split into two branches from M_Resident_ (Figure 2G; Figure S5G). Genes on developmental trajectories with distinct trends are divided into four groups and subjected to GO analysis. The results show that genes biased towards branch 1 are enriched in various immune‐related pathways. Genes biased towards branch 2 are enriched in neuroprotective function pathways (Figure 2H; Table S9). Moreover, the expression of pathways related to cell differentiation and activation increases along branch 1, while it decreases along branch 2 and is predominantly enriched in the M_Ikzf1_ (Figure S5H,I). These findings suggest that the M_Ikzf1_ and M_Ptn_ delineate distinct pathways of microglial post‐ICH, with the M_Ikzf1_ displaying a heightened affinity for immune‐related pathways.

To identify key target genes for enhancing the ICH prognosis through post‐Exo, we analyzed the miRNA in Exo and identified 178 miRNAs targeting 346 genes (Figure S6A–C, Table S10). GO analysis enriched in pathways related to myeloid cell differentiation all contain the *Ikzf1* (Figure S6D, Table S11), indicating that Exo may modulate the transition of microglia subclasses by influencing critical genes like *Ikzf1*.

Annotate subclasses in peri‐hematoma area on STs and analyze cell‐chat (Figure 3A,B; Figure S7A). The cell‐chat in scRNA‐Seq (Figure 3C,D; Figure S7B) and STs (Figure 3E; Figure S7C) indicate a notable elevation in the PTN pathway post‐Exo, with particular emphasis on the PTN‐PTPRZ1 axis. Gene co‐expression and immunofluorescence (Figure 3F–G) reveal an enhanced co‐localization of PTN‐PTPRZ1 post‐Exo.

**FIGURE 3:**
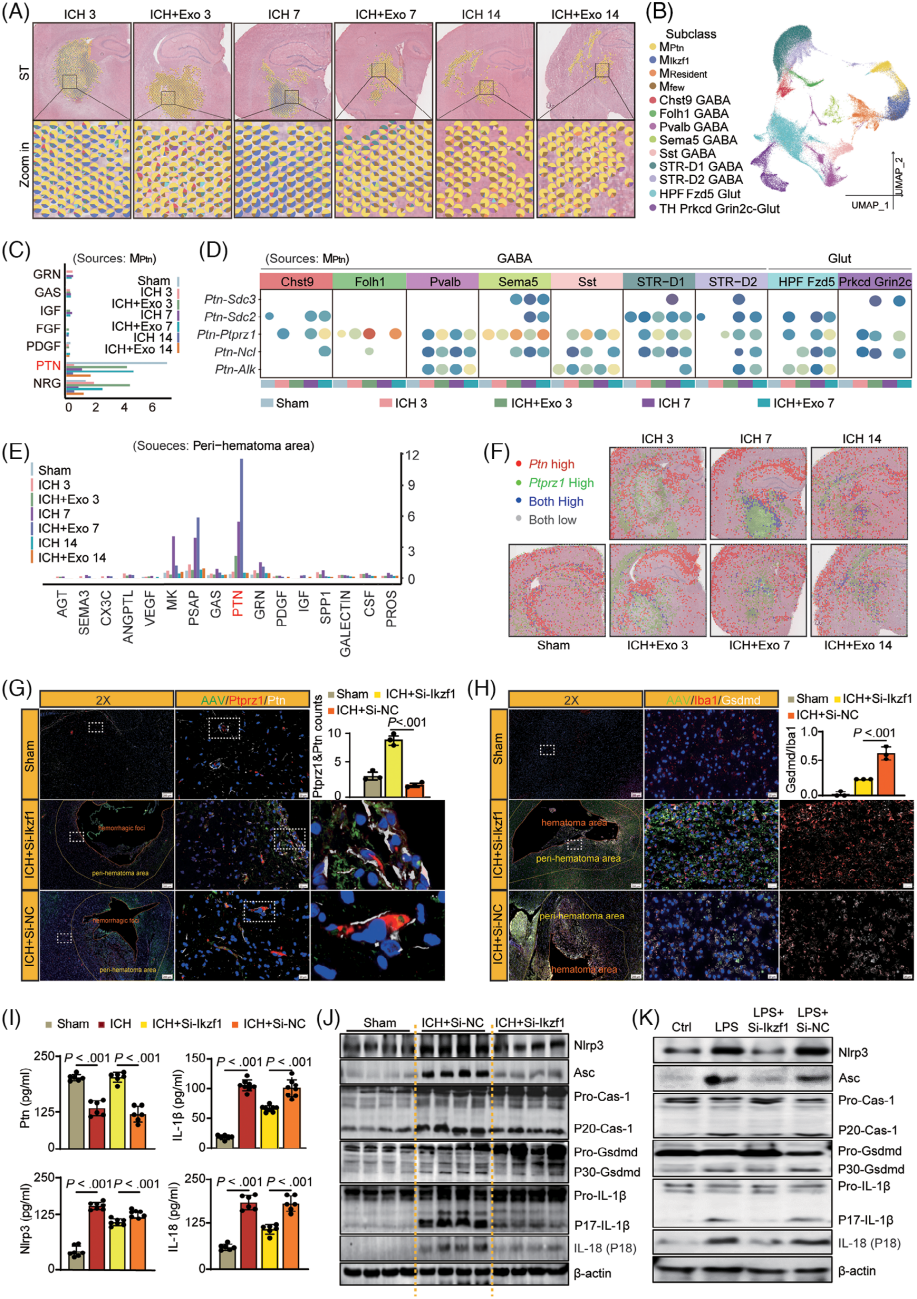
(A) Comprehensive analysis of gene sets derived from scRNA‐seq data was conducted, applying RCTD to deconvolve ST data. The top section includes a pie chart predicting spot distribution in the peri‐hematoma region of the ST data while the bottom section features a zoomed‐in view of the top image, with a pie chart visually representing the distribution of individual subclasses. (B) UMAP plot illustrating subclass matching in the peri‐hematoma and hematoma areas (The classification of cell subtypes is completed through the Mapmycell website). (C) Analyze cell‐chat between different subclasses in the peri‐hematoma area and hematoma area using single‐cell sequencing data. Determine signalling pathways based on their contribution to overall information flow, with M_Ptn_ serving as the signal source. The length of the bars in the chart reflects the strength of the signalling pathways. (D) Analyze cell‐chat between different subclasses in the peri‐hematoma area and hematoma area using single‐cell sequencing. Present the strength of signal transduction between MPtn and neurocytes across all subtypes of the PTN pathway with a bubble chart. (E) Analyze cell‐chat between different regions in STs. Rank significant signalling pathways by the weight of their contribution to overall information flow, using the peri‐hematoma area as the signal source. The length of the bar chart represents the strength of the signalling pathways. (F) Co‐localization of *Ptn* and *Ptprz1* expression in STs at 3–14 dpi, with red indicating high *Ptn* expression, green indicating high *Ptprz1* expression, and blue indicating co‐expression of both. (G) Co‐immunostaining of Ptn&Iba1 in the peri‐hematoma area regions and quantitative analysis in 3 dpi, *n* = 3 rats per group. (H) Co‐immunostaining of Gsdmd&Iba1 in the peri‐hematoma area regions and quantitative analysis in 3 dpi, *n* = 3 rats per group. (I) Protein expression of Ptn, IL‐1β, Nlrp3 and IL‐18 in the brain was detected using ELISA assay, *n* = 6 rats per group. (J) Protein expression of Ptn, IL‐1β, Nlrp3, and IL‐18 in the brains of Sham, ICH, ICH+Si‐Ikzf1 and ICH+Si‐NC group (3 dpi), *n* = 4 rats per group. The original western blot images are presented in Figure S18, where the protein molecular weight markers were labelled. (K) Protein expression of Nlrp3/β‐actin, Asc/β‐actin, caspase‐1 (P20/Pro), IL‐1β (P17/Pro), IL‐18/β‐actin and Gsdmd (P30/Pro) in Bv‐2 cells detected using western blot, *n* = 3 per group The original western blot images are presented in Figure S19, where the protein molecular weight markers were labelled.

The impact of Ikzf1 on the microglial state was explored by a virus to silence Ikzf1 in the brain and Bv‐2 cells (Figure S8A,B). Immunofluorescence revealed the co‐localization of Iba1&Ikzf1 was reduced, whereas the co‐localization of Iba1&Ptn increased (Figure S8C,D), post‐Si‐Ikzf1. Activation of Nlrp3‐inflammasomes triggers pyroptosis and the secretion of pro‐inflammatory cytokines.^5^, ^6^, ^7^ Si‐Ikzf1 treatment markedly decreased the co‐expression of Iba1&Gsdmd (Figure 3H). Furthermore, it upregulated the expression of Ptn in brain and Bv‐2 cells while simultaneously downregulating the levels of Nlrp3, IL‐18, and IL‐1β proteins (Figure 3I; Figure S8E). The expression of Nlrp3 inflammasome‐related proteins, including Asc, cleaved‐caspase‐1, Gsdmd, IL‐1β, and IL‐18, was significantly inhibited (Figure 3J,K; Figure S9, S10). These findings indicate that Si‐Ikzf1 suppresses the excessive activation of the Nlrp3 inflammasome, consequently alleviating inflammation in the ICH model.

Microglia pyroptosis can result in cell membrane perforation, elevation of inflammatory factors in the microenvironment while triggering neuronal apoptosis.^2^, ^8^

TEM revealed a reduction in the formation of Gsdmd‐N pores in Bv‐2 cells and the brain (Figure 4A,B) post‐Si‐Ikzf1. To assess the influence of pro‐inflammatory factors released from pyroptosis‐microglia on neuronal apoptosis, the conditioned medium from LPS‐BV‐2 cells was utilized in primary‐mouse neurons and Sh‐Sy5y cells (Figure S11). Flow cytometry (Figure 4C; Figure S12) and apoptosis staining (Figure 4D) demonstrated induction of apoptosis by this conditioned medium, which was subsequently reversed post‐Si‐Ikzf1. Furthermore, Si‐Ikzf1 treatment decreased the ratio of Bax/Bcl‐2 and the cleaved caspase‐3, caspase‐9, and PARP1 in Sh‐Sy5y cells and the brain (Figure 4E,F; Figures S13, S14). TUNEL and co‐immunofluorescence test (Figure S15A; Figure 4G) supported the findings. These data indicate that Si‐Ikzf1 not only promotes the transition from M_Ikzf1_ to M_Ptn_ but also significantly reduces neuronal apoptosis in the microenvironment.

**FIGURE 4:**
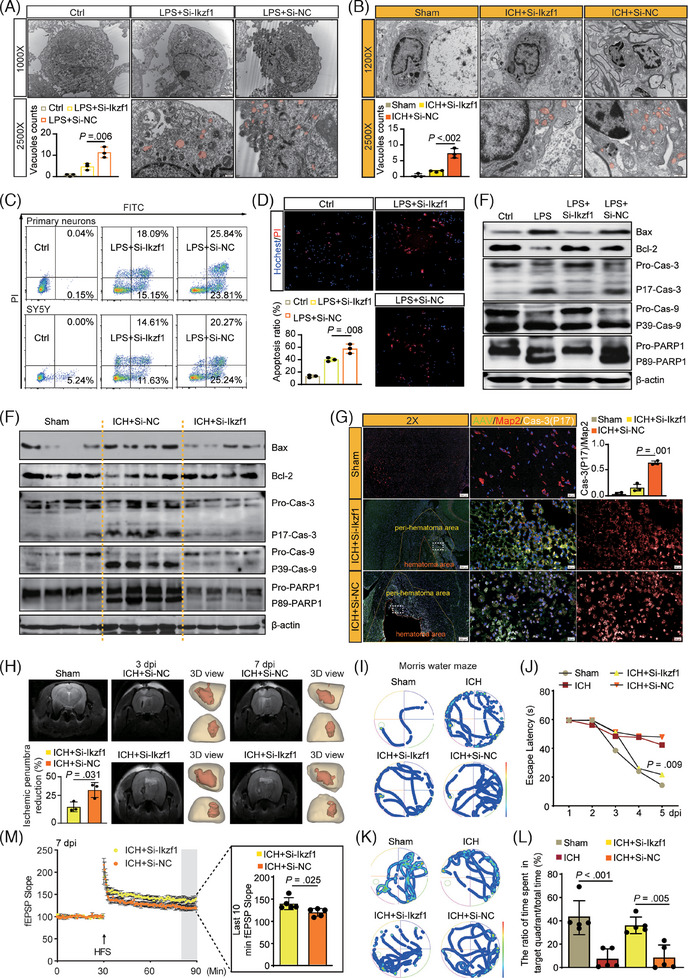
(A) Representative transmission electron microscope (TEM) images of BV‐2 cells are shown. The bottom image displays a higher magnification inset of the top image, where the red shading highlights the focal pyroptosis vesicles in the BV‐2 cells. Bar charts indicate the vesicle counts. Magnification: ×10 000, scale bar: 2 µm; *n* = 3 per group. (B) Representative TEM of microglia (Under the electron microscope, the microglia exhibit deep staining, with flattened or serrated nuclei). The bottom image showed the inset of the top image at higher magnification, the red shading marks the focal pyroptosis vesicles in the microglia. Bar charts indicate the vesicle counts. Magnification: ×10 000, scale bar: 2 µm; *n* = 3 rats per group. (C) The cell apoptosis in LPS‐treated conditioned medium cultured mouse‐primary neuron and Sh‐Sy5y cells was assessed by flow cytometry using Annexin V‐PE apoptosis detection kit. (D) Hoechst 33342/PI staining of LPS‐treated conditioned medium cultured mouse primary neuron. Bar charts indicate the PI/Hoechst ratio, *n* = 3 per group. (E) Protein expressions of the ratios of Bax/Bcl2, Capase‐3 (P17)/ Capase‐3 (Pro), Capase‐9 (P39)/ Capase‐9 (Pro), and PARP1 (P89)/ PARP1 (Pro) in LPS‐treated conditioned medium cultured Sh‐Sy5y cells detected using western blot. The original western blot images are presented in Figure S20, where the protein molecular weight markers were labelled. (F) Protein expressions of the ratios of Bax/Bcl2, Capase‐3 (P17)/ Capase‐3(Pro), Capase‐9 (P39)/ Capase‐9 (Pro), and PARP1 (P89)/ PARP1 (Pro) in brain tissue were detected using western blot. The original western blot images are presented in Figure S21, where the protein molecular weight markers were labelled. (G) Co‐immunostaining of Caspase‐3 (P17) &Map2 in the peri‐hematoma area and quantitative analysis in 3 dpi, *n* = 3 rats per group. (H) Magnetic Resonance Imaging (MRI) T2*WI images and 3D modelling of volume changes in the peri‐hematoma area. Bar charts showing the percentage reduction in peri‐hematoma area, *n* = 3 rats per group. (I–L) Cognitive assessment of rat in Sham, ICH+Si‐Ikzf1, and ICH+Si‐NC group using the Morris water maze (MWM) test (*n* ≥ 5). Escape latencies are depicted in (J), and the proportion of time spent in the target quadrant is relative to total time in (L). (M) Time course of field excitatory postsynaptic potential (fEPSP) slope in CA3‐CA1 long‐term potentiation (LTP) in hippocampal slices from ICH+Si‐NC and ICH+Si‐Ikzf1 groups. The arrow indicates the point of high‐frequency stimulation. Bar charts inside the border show the normalized 10 min average fEPSP slope 60 min post‐high‐frequency stimulation, relative to the last 10 min baseline.

MRI revealed a significant reduction in the volume of the peri‐hematoma area post‐Si‐Ikzf1 (Figure 4H). The mNSS and Foot‐fault testing indicated that Si‐Ikzf1 partially reversed the sensorimotor deficits post‐ICH (Figure S15A,B). MWM test results confirmed that Si‐Ikzf1 alleviated cognitive impairments in ICH‐rats (Figure 4I–L). TEM showed that Si‐Ikzf1 improved neuronal demyelination (Figure S14D,E). Patch‐clamp recordings revealed that Si‐Ikzf1 enhanced long‐term potentiation in the hippocampus CA3‐CA1 of ICH‐rats (Figure 4M, Table S14).

The Ikzf1‐SIRT1 axis regulates macrophage activation through AMPK signalling.^9^ Our study showed that Si‐Ikzf1 reduced Ikzf1 expression while increasing SIRT1 and p‐AMPK expression in LPS‐Bv‐2 cells and ICH‐brain (Figure S15F,G). These results suggest that the Ikzf1‐SIRT1 axis influences microglial function via the AMPK pathway.

Overall, downregulating Ikzf1 promotes the differentiation of post‐ICH microglia toward a neuroprotective phenotype, reducing inflammation, pyroptosis, and neuronal apoptosis. Ikzf1 represents a promising therapeutic target for ICH, providing new insights into neuroinflammation and neuronal injury treatment.

## AUTHOR CONTRIBUTIONS

Ruxiang Xu designed the experiments. Wenqiao Qiu, Jie Mei, and Jie Tian performed bioinformatic analysis. Mingjun Gao, Lili Guo, and Tao Xu performed confocal microscopy. Wei Liu, Jianwei Zhu, and Yi Zhang analyzed histological data. Huan Xiong, Yao Xie, and Xiaolin Hou performed miRNA sequencing and scRNA‐sequencing. Xinda Li, Yangyang Wang, Anguo Wu, and Mingjun Gao performed brain MRI experiments. Wenqiao Qiu and Jie Mei wrote the manuscript and edited by Lulin Huang. All authors read and approved the manuscript.

## CONFLICT OF INTEREST STATEMENT

The authors declare no conflict of interest.

## FUNDING INFORMATION

This work was supported by the National Natural Science Foundation of China (project nos. 82171355, 81971295, 81671189, 82271105). National Key Research and Development Program of China (2023YFF1204200, 23ZYZYTS0271). Special project of Sichuan Province for central guidance of local scientific and technological development (2023ZYD0059).

## ETHICS STATEMENT

Ethics approval was obtained from the Medical Ethics Committee of Sichuan Provincial People's Hospital (approval number: 2022–154).

## Supporting information



Supporting Information

## Data Availability

All sequencing data are available from the corresponding author (R.X.) upon a reasonable request.
