# Supplementary material for: Spatiotemporal transcriptome and scRNA sequencing analysis reveals that IKFZ1‐mediated microglia underlies a therapy for intracerebral hemorrhage
Source: Clin Transl Med. 2024 Dec 10;14(12):e70127. doi: 10.1002/ctm2.70127 (PMC11631562; doi:10.1002/ctm2.70127)
Supplement: Supplementary file 1 — Supporting Information [file CTM2-14-e70127-s001.docx]

**Supplementary data**

***Spatiotemporal transcriptome and scRNA sequencing analysis reveals that IKFZ1-mediated microglia underlies a therapy for intracerebral hemorrhage***

Wenqiao Qiu^1^, Jie Tian^1^, Gao Mou^5^, Lili Guo^1^, Tao Xu^6^, Wei Liu^1^, Jianwei Zhu^1^, Yi Zhang^6^, Xiaolin Hou^1^, Yao Xie^2^, Huan Xiong^1^, Xinda Li^1^, Yangyang Wang^1^, Mingjun Gao^1^, Anguo Wu^7^, Longyi Chen^1^, Jie Mei ^2✉^, Lulin Huang ^3,4^^✉^ and Ruxiang Xu^1✉^

^1^Department of Neurosurgery, Sichuan Provincial People's Hospital, School of Medicine, University of Electronic Science and Technology of China, Chengdu 610072, China

^2^Department of Obstetrics &. Gynecology, Sichuan Provincial People's Hospital, University of Electronic Science and Technology of China, Chengdu 610072, China

^3^Sichuan Provincial Key Laboratory for Human Disease Gene Study and the Center for Medical Genetics, Department of Laboratory Medicine, Sichuan Academy of Medical Sciences and Sichuan Provincial People's Hospital, University of Electronic Science and Technology of China, Chengdu, China.

^4^Research Unit for Blindness Prevention of Chinese Academy of Medical Sciences (2019RU026), Sichuan Academy of Medical Sciences & Sichuan Provincial People's Hospital, Chengdu, Sichuan, China

^5^Department of Neurosurgery, Chinese PLA General Hospital, Beijing 100853, China.

^6^Biomanufacturing and Rapid Forming Technology Key Laboratory of Beijing, Department of Mechanical Engineering, Tsinghua University, Beijing 100084, People's Republic of China.

^7^Sichuan Key Medical Laboratory of New Drug Discovery and Drugability Evaluation; Luzhou Key Laboratory of Activity Screening and Druggability Evaluation for Chinese Materia Medica; School of Preclinical Medicine; Key Laboratory of Medical Electrophysiology of Ministry of Education; School of Pharmacy; Southwest Medical University; Luzhou, China.

 ✉ Correspondence should be addressed to Ruxiang Xu, Lulin Huang or Jie Mei:

Ruxiang Xu, Department of Neurosurgery, Sichuan Provincial People's Hospital, University of Electronic Science and Technology of China, 32 The First Ring Road West 2, Chengdu, Sichuan, 610072, China. Email: xuruxiang1123@uestc.edu.cn

Lulin Huang, Sichuan Provincial Key Laboratory for Human Disease Gene Study, Sichuan Provincial People's Hospital, University of Electronic Science and Technology of China, Chengdu, China, 32 The First Ring Road West 2, Chengdu, Sichuan, 610072, China. Email: huangluling@yeah.net

Jie Mei, Department of Obstetrics and Gynecology, Sichuan Provincial People's Hospital, University of Electronic Science and Technology of China, 32 The First Ring Road West 2, Chengdu, Sichuan, 610072, China. Email: meijie@med.uestc.edu.cn

# Material and Methods

**Key Resources Table**

All the reagents used, their sources and identifiers are listed in the Supplementary information, Table. S15

**Cell culture**

Sh-Sy5y and BV-2 cells were acquired from the American Type Culture Collection (Rockville, MD, USA). All cells were maintained in DMEM supplemented with 10% FBS and 1% penicillin-streptomycin (Invitrogen, Scotland, UK) within a 5% CO2 incubator at 37°C.

**Mouse primary neuron isolation and culture**

In this study, mouse primary neurons were obtained from the cerebral cortices of C57BL/6 mice within five days of birth. The brain was carefully dissected under a stereomicroscope, removing the meninges and blood vessels to isolate the cerebral hemisphere. The cerebral hemisphere tissue was then cut using microsurgical forceps and digested with 0.175% trypsin (50:1 DNase) for 10 minutes. The digestion was halted by adding an equal volume of culture medium with 10% fetal bovine serum (FBS; Gibco). The cells were then dispersed by gentle pipetting to achieve a single-cell suspension in the culture medium, which was subsequently filtered through a 40μm sieve. Cell subculture was performed every 5-7 days.

**Animals**

In this study, adult male Sprague–Dawley rats weighing 250–300 g were obtained from Ensiweier Biotechnology Co, Ltd. (Chongqing, China). The rats were housed under specific-pathogen-free conditions and allowed free access to fodder and water. All experimental procedures followed the National Institute of Health's guidelines for the Care and Use of Laboratory Animals and were approved by the ethics committee of Sichuan Provincial People’s Hospital.

**Spatial transcriptome assay and sequencing**

OCT-embedded brain tissues were cryosectioned at a thickness of 10 μm and mounted on Visium spatial gene expression slides (10x Genomics, #1000184; Pleasanton, CA, USA). Tissues were stained with H&E and microscopic images were captured with a 10× objective. Brain tissues were then permeabilized, allowing RNA to diffuse and bind to the slide surface. Using the Spatial Tissue Optimization procedure (10× Genomics, #1000193), permeabilization was performed for 12 min. Then, cDNA was synthesized on-slide from the immobilized RNA and the cDNA was collected to generate sequencing libraries containing unique sample indices, as well as P5 and P7 primers for Illumina-compatible sequencing. Paired-end sequencing (2 × 100) was performed on an Illumina NovaSeq 6000 instrument (San Diego, CA, USA).

**Preparation of single-cell suspensions from rat brain tissue**

Rat brain tissues were collected and rinsed three times with sterile PBS. A maximum of 200 mg brain tissue was transferred to tubes containing 4.9 mL HEPES buffer, 10 μL DNase I solution, and 100 μL collagenase D solution (2 mg/ml) per 150–450 mg brain tissue. The cells were filtered through mesh cell strainers (70 μm) and collected in 50 mL centrifuge tubes. The mesh cell strainers were washed with 5 mL HEPES buffer at RT, and the resulting cell suspensions were centrifuged at 250 × g for 15 min. After the removal of the supernatants, the cells were resuspended with the desired volume of PEB buffer and prepared for single-cell sequencing.

**Single-cell sequencing library preparation and quality control**

ScRNA-Seq libraries were prepared using Single Cell 3' v2 chemistry. Next-generation sequencing was performed on the Illumina NextSeq500 using a transcript length of 150 bp. CellRanger Count v3.0.2 (10× Genomics) was used to perform sample de-multiplexing, barcode processing, and single-cell gene UMI counting. Seurat v4 was used to perform dimensionality reduction, clustering, and visualization. Cells that had fewer than 200 detected genes were filtered out. SCTransform and Find Variable Features were used to normalize and scale the data. All samples were merged using the CCA method ^1^. The principal component analysis was performed using Seurat’s RunPCA function. The first 20 principal components were used for UMAP visualization and Louvain clustering using Seurat. CellMarker and Single were used to perform subcluster analysis. FindMarker was used to identify the differentially expressed genes based on a fold-change cutoff of 0.2 and an adjusted *P*-value < 0.05.

**Mapmycell**

MapMyCells (RRID:SCR_024672). Upload droplet_library.h5ad to the site via the file system or drag and drop. Choose 10x Whole Human Brain (CCN202210140) as the Reference Taxonomy. Choose the desired Mapping Algorithm (in this case Hierarchical Mapping). Click Start and wait ~5 minutes. (Optional) You may have a panel on the left that says Map Results. Where can also wait for run to finish. When the mapping is complete, will have an option to download the tar file with mapping results. Copy [NUMBER].csv to current working directory and rename it “droplet_library_mapping.csv”). Now go back to R and continue the script below.

**Spatial transcriptomics** **library preparation**

We used the 10× Genomics Spatial RNAseq Visium platform for the spatial transcriptomics experiments. The oligos on each spot featured a PCR handle, unique spatial barcode, Unique Molecular Identifier (UMI), and a poly-dT-VN tail for capturing the 3’ end of mRNA molecules. Spatially tagged cDNA libraries were built using the 10× Genomics Visium Spatial Gene Expression 3’ Library Construction V1 Kit. cDNA libraries were sequenced on an Illumina NextSeq 500/550 using 150 cycle high output kits. Fluidigm (South San Francisco, CA, USA) frames around the capture area on the Visium slide were aligned manually and spots covering the tissue were selected using Loop Browser 4.0.0 software (10x Genomics). Sequencing data were then aligned to the chicken reference genome using the Space Ranger 1.0.0 pipeline to derive a feature spot-barcode expression matrix.

**Celltype annotation**

After quality control, a total of 278,394 cells were profiled for data analysis. Twelve distinct celltypes were identified through the utilization of the MapMyCells (RRID:SCR_024672)^2^, in conjunction with the marker genes for individual celltypes, including Astro-Epen, LGE GABA, CGE GABA, MGE GABA, DG-IMN Glut, Immune, IT-ET Glu, LSX GABA, CTX-L6b Glut, OPC-Oligo, TH Glut, and Vascular .

**RNA sequencing**

Total RNA in exosomes were extracted using the Total Exosome RNA and Protein Isolation Kit (Invitrogen, New York, NY, USA). Then the cDNA library was sequenced on an Illumina Hiseq 2000. Raw reads were collected using the Illumina analysis software.

**Cell trajectory analyses**

Using Monocle2 for a pseudo-time-based cell trajectory analysis, significantly different genes between two groups were used to run trajectory analysis, with an adjusted *P*-value < 0.05 ^3^.

**Transcripts regulatory network analysis**

SCENIC workflow was used to infer TF regulatory networks^4^. Three R/Bioconductor packages including GENIE3, RcisTarget, and AUCell were used to identify potential TF targets and regulons, then to score the activity of regulons on single cells.

**GSEA and Pathway enrichment analysis**

Molecular Signatures Database GO gene sets (C5) were used for GSEA enrichment analysis by R package ClusterProfiler ^5^ An adjusted P-value < 0.05 was used as a cutoff. The differential genes were mapped to human genome resources using the genome-wide genes as background and enriched the pathway including the KEGG pathway and GO biological processes pathway.

**Western blot**

Total protein content from cells or brain tissues was extracted at 4°C using RIPA lysis buffer. Lysates were collected and centrifuged at 5200 g for 15 min at 4°C. Subsequently, the supernatant was transferred to new tubes. Protein concentrations were determined utilizing the Bradford Protein Assay Kit (Beyotime Biotechnology Co, Ltd. Shanghai, China). Proteins were subjected to electrophoresis and transferred onto polyvinylidene fluoride membranes (PALL, New York, NY, USA). The membranes were then blocked in Quick Block Western (Proteintech, Wuhan, China) and incubated with primary antibodies (1:1000) overnight at 4 °C, followed by incubation with secondary antibodies (1:1000) for 2h at 30 °C. After washes with TBST, bands were visualized using UltraSignal™ ECL Western blotting detection reagent (4A Biotech Co., Ltd., Beijing, China) and imaged with a Tanon 4600 Imaging System (Tanon, Shanghai, China). Protein band intensities, representing relative expression levels, were quantified using ImageJ software (National Institutes of Health, Bethesda, MD, USA).

**Flow cytometry analysis**

**Flow cytometry apoptosis detection experiment**

The Annexin V-FITC/PI apoptosis detection kit (4A Biotech Co., Ltd., Beijing, China) was used to analyze cell apoptosis. Following treatment, cells were collected and centrifuged at 1500 rpm for 8 min. After removing the supernatant, the cells were resuspended in 500 μL of solution with propidium iodide (PI) and Annexin V-FITC solution. After a 15-minute incubation at room temperature, cells were analyzed using NovoCyte Quanteon 4016 (Agilent Technologies, Santa Clara, CA, USA). Data acquisition and cell viability analysis were conducted using FlowJo v10.0 software (Leland Stanford Junior University, Stanford, CA, USA).

**Isolation and flow cytometry-based subpopulation identification experiment of** **rats primary microglial**

In this study, primary microglia from rat brain slice tissue were obtained from the peri-hematoma and hematoma areas of rats in the Sham, ICH, and ICH+Exo groups. The tissue was then dissected using microsurgical forceps and digested with 0.175% trypsin (50:1 DNase) for 10 minutes. The enzymatic digestion was stopped by adding an equal volume of culture medium containing 10% fetal bovine serum (FBS; Gibco). The cells were then gently pipetted to achieve a single-cell suspension in the culture medium, followed by filtration through a 40 μm sieve. CD11b/c-expressing cells were isolated using MicroBeads, rat/CD11b/c (Miltenyi Biotec., Germany). Immunostaining of cell membrane proteins involved CD45-Alexa Fluor 700 and CD11b/c-FITC antibodies. After a 30-minute incubation at 4 °C, the cells were fixed using a cell perforation and fixation kit, followed by intracellular protein staining with Ptn-CoraLite® Plus 750 and Ikzf1-CoraLite® Plus 405. Subsequently, after a 45-minute incubation at 4 °C, cell analysis was performed using the NovoCyte Quanteon 4016 (Agilent Technologies, Santa Clara, CA, USA). Data acquisition and cell viability analysis were conducted using FlowJo v10.0 software (Leland Stanford Junior University, Stanford, CA, USA).

**Transmission electron microscopy**

**BV-2 cells**

BV-2 cells were fixed in 0.5% phosphate-buffered glutaraldehyde overnight at 4°C.

**Rat brain tissue**

Rat brain tissue was fixed in 2.5% phosphate-buffered glutaraldehyde overnight at 4°C, washed in PBS, postfixed in 1% osmic acid, washed with Milloning’s buffer, dehydrated in a graded ethyl alcohol series, exposed to propylene oxide and embedded using EMbed 812 kits (Electron Microscopy Sciences, Hatfield, PA, USA). Following polymerization, semithin sections of Epon blocks were cut at 1 μm. Images were acquired utilizing a transmission electron microscope (JEM-1400FLASH).

**Behavioral Experiments**

**Morris water maze test**

The MWM apparatus consisted of a circular (160 cm diameter, 80 cm height), dark gray tank filled with 25°C±2 water. An escape platform was situated 1 cm below the water surface in the center of three quadrants. During the training period, each rat was trained twice daily for five consecutive days. Following the five-day training period, cognitive function was assessed as described previously ^5^. Rats were released into the water from the same quadrant, and their escape latency was recorded. Concurrently, the number of mice that entered the quadrant containing the original platform was documented using the Animals Behavior Analysis System (Zhongshi Science & Technology Co., Ltd., Beijing, China).

**Modified neurological severity scores**

The mNSS score was assessed as previously reported ^6^. It includes motor, sensory, reflex, and balance tests, as well as 1, 3, 7, 14, and 28 dpi recordings of neurological function.

**Foot-fault test**

The foot-fault test was described previously ^7^, and was performed to evaluate motor function. Rats were placed on a grid and allowed to move along it. If a paw fell between the wire or slips, it was recorded as a foot fault. The total number of steps that the rats used to cross the grid was counted, and the total number of foot faults for each forelimb was recorded. The percentage of forelimb faults to the total number of steps was calculated.

**Enzyme-Linked Immunosorbent Assay**

Rat Nlrp3, IL-1β, IL-18 (RUIXIN BIOTECH Co., Ltd., Quanzhou, China), and Ptn Quantizing ELISA Kits (Cloud-Clone Corp Co, Ltd. Wuhan, China) were used to measure the quantity of protein secreted into the cell culture supernatant and in rat brain tissue, according to the manufacturer's instructions. Sample absorbance was measured at 450 nm within 30 min.

**Hoechst 33342 and PI staining**

After treatment, primary neuronal cells were fixed in 4% paraformaldehyde and subjected to staining with 5 mg/L Hoechst 33342 and 5 mg/L PI solution for 10 min. After that, the slides were taken out to air-dry and mounted with FluorSave™ mounting media (Calbiochem, San Diego, CA, USA). The representative images showing the blue and red signals were captured and merged by a fluorescence microscope. Cell death was analyzed by calculating the percentage of PI/Hoechst.

**Immunofluorescent staining**

Apoptotic cells and the expression of Iba1, Ptn, Gsdmd, Caspas-3 (P17), and Map2 in rat brain tissue were detected using an immunofluorescence method and TUNEL staining, respectively. Briefly, the sections were blocked in 10% goat serum with 0.1% Triton X-100 for 0.5 h. Afterward, the sections were incubated with primary antibodies overnight at 4°C, followed by a 2 h incubation with fluorescent dye-conjugated second antibodies. After incubation, the sections were counterstained with DAPI and photographed by a Zeiss LSM 800 confocal microscope. The mean fluorescence intensity was measured by ImageJ software.

**Patch clamp technique**

**Experimental plan**

1. Rat brain slices were prepared by dissecting the hippocampus and cutting the tissue into 380 μm-thick brain slices. (2) After 1–2 h of incubation, recording was started. (3) We recorded the process CA3–CA1 LTP. We recorded the baseline with a stimulus intensity that could cause a maximum response amplitude of 40%–50% and considered it the base stimulus intensity. After stabilizing the reaction, >30 min was recorded as the baseline. The fluctuation range of the baseline slope was required to be < 10%. Immediately after inducing LTP basal stimulation, HFS was run at 100 Hz per strand, lasting for 1 s. The stimulation was performed three times with an interval of 10 seconds between stimulations. We recorded the post-induction response for 60 min using a single test pulse stimulation.

**Data processing**

The slope of fEPSP changes over time: For each LTP response, the slope of fEPSP after stimulation was standardized with the mean (100%) of the baseline fEPSP slope as a reference. We calculated the mean of the data at the same time point in the experiment. The degree of LTP was expressed as a percentage of the baseline fEPSP slope. To compare long-term synaptic plasticity between groups, for each LTP response, the mean of 20 signals at 10 min before and after stimulation was used as a reference for standardization, and the mean was taken. Afterward, we took the mean for the same group.

**Acquisition of** **MRI data for rat brain images**

Anesthesia was induced with 5% isoflurane in a 2:1 mixture of N_2_O and carbogen (95% oxygen, 5% co2) using a small animal anesthesia machine (Zhongshi Science & Technology Co, Ltd., Beijing, China). Rats were transferred to a cradle and fixed into place using ear bars and an incisor bar. The MRI data was collected in accordance with the methods described by Prior et al^8^.

**RERENCES**

1. Herradon G, Perez-Garcia C. Targeting midkine and pleiotrophin signalling pathways in addiction and neurodegenerative disorders: recent progress and perspectives. *Br J Pharmacol*. Feb 2014;171(4):837-48. doi:10.1111/bph.12312

2. Yao ZZ, van Velthoven CTJ, Kunst M, et al. A high-resolution transcriptomic and spatial atlas of cell types in the whole mouse brain. *Nature*. Dec 14 2023;624(7991)doi:10.1038/s41586-023-06812-z

3. Trapnell C, Cacchiarelli D, Grimsby J, et al. The dynamics and regulators of cell fate decisions are revealed by pseudotemporal ordering of single cells. *Nat Biotechnol*. Apr 2014;32(4):381-386. doi:10.1038/nbt.2859

4. van de Sande B, Flerin C, Davie K, et al. A scalable SCENIC workflow for single-cell gene regulatory network analysis. *Nat Protoc*. Jul 2020;15(7):2247-2276. doi:10.1038/s41596-020-0336-2

5. Wu T, Hu E, Xu S, et al. clusterProfiler 4.0: A universal enrichment tool for interpreting omics data. *Innovation (Camb)*. Aug 28 2021;2(3):100141. doi:10.1016/j.xinn.2021.100141

6. Wu H, Shao A, Zhao M, et al. Melatonin attenuates neuronal apoptosis through up-regulation of K(+) -Cl(-) cotransporter KCC2 expression following traumatic brain injury in rats. *J Pineal Res*. Sep 2016;61(2):241-50. doi:10.1111/jpi.12344

7. Zheng J, Sun Z, Liang F, et al. AdipoRon Attenuates Neuroinflammation After Intracerebral Hemorrhage Through AdipoR1-AMPK Pathway. *Neuroscience*. Aug 1 2019;412:116-130. doi:10.1016/j.neuroscience.2019.05.060

8. Prior MJW, Bast T, McGarrity S, et al. Ratlas-LH: An MRI template of the Lister hooded rat brain with stereotaxic coordinates for neurosurgical implantations. *Brain Neurosci Adv*. Jan-Dec 2021;5:23982128211036332. doi:10.1177/23982128211036332

# Supplementary Figure

**Figure S1**


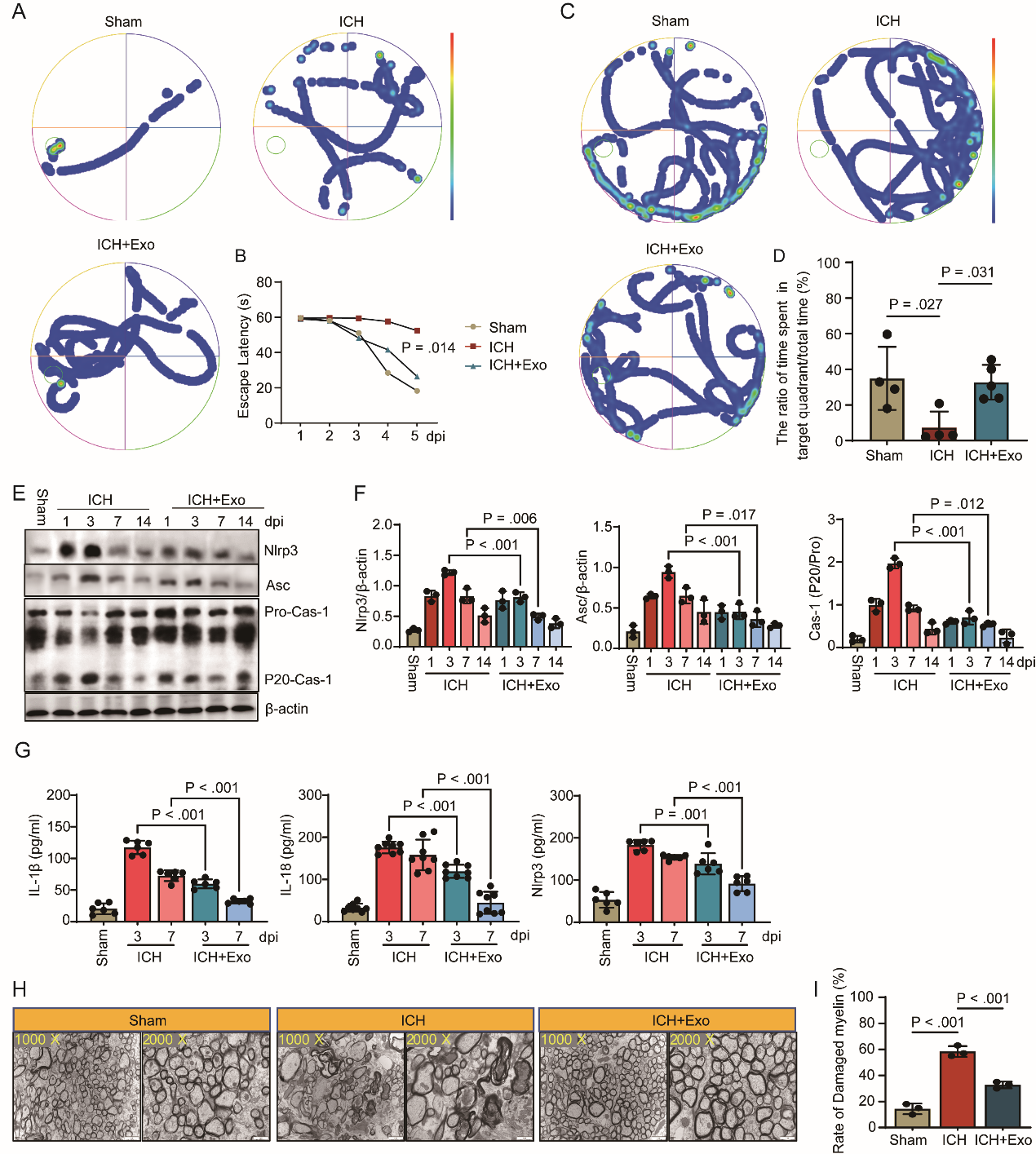


**Figure S1** (A, C) The cognitive functions in sham, ICH and ICH+Exo group rats were evaluated by Morris water maze (MWM) tests. (B) Line charts indicate the escape latencies. (D) Bar charts indicate the ratio of time spent in the target quadrant/total time (n≥4). (E) Brains of rats in the ICH and ICH+Exo groups; protein expression was detected using Western blot. The original Western blot images are presented in Figure.S17, where the protein molecular weight markers were labeled. (F) Bar charts indicate the ratios of Nlrp3/β-actin, Asc/β-actin and caspase-1 (P20)/ Pro-Caspase-1 in brain (n=3). (G) Brain tissue from the Sham, ICH, and ICH+Exo groups (3–7 dpi). Protein expression was detected using ELISA assay (n=6). (H) Representative electron micrographs showing the ultrastructure of rat brains in the Sham, ICH, and ICH+Exo groups (3 dpi). (I) Bar charts indicate the ratios of damaged myelin (n=3).

**Figure S2**

**
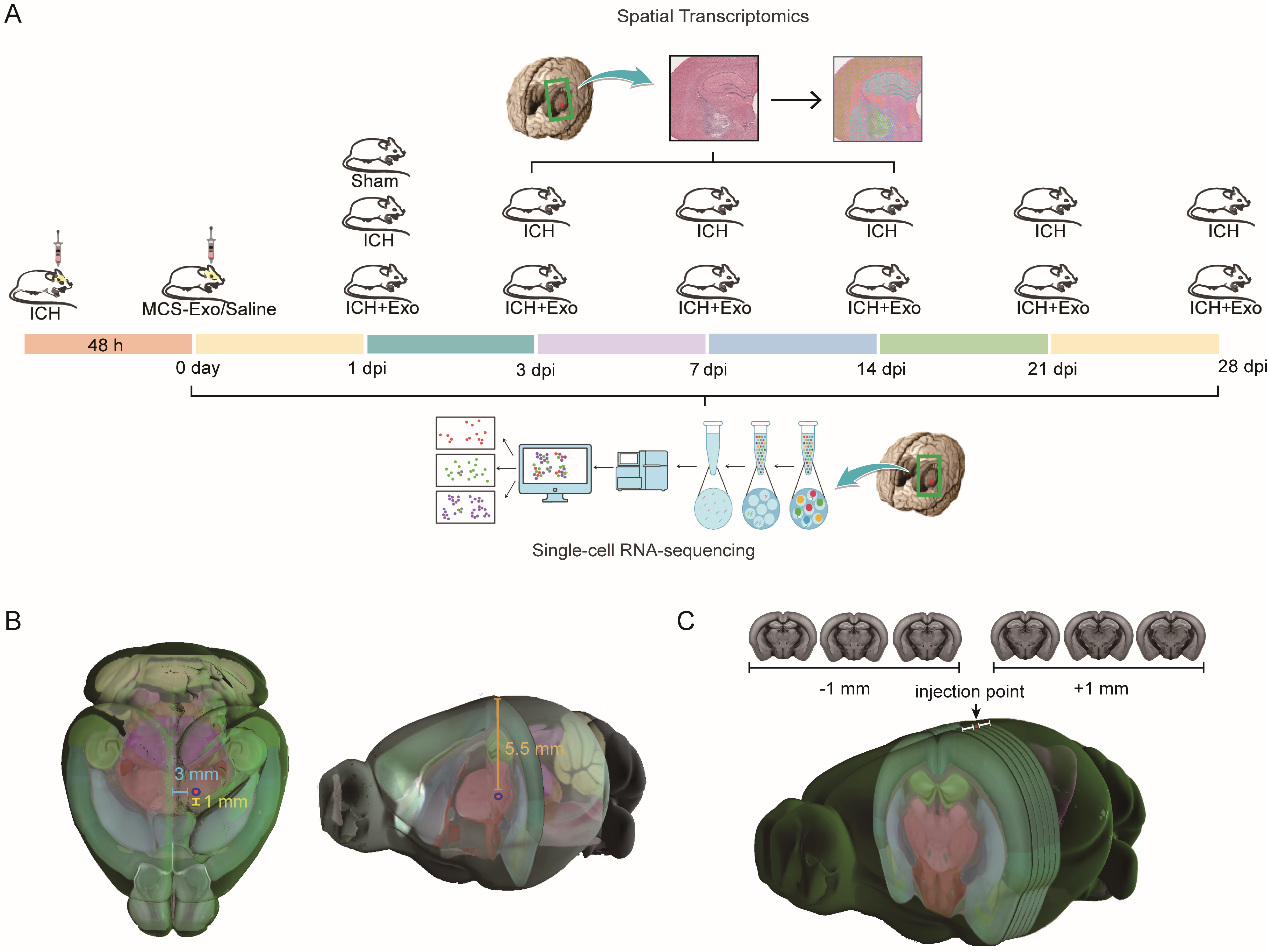
**

**Figure S2** (A) Workflow of the entire study, including sample acquisition, sequencing and integration analysis. After performed on type IV collagenase-induced ICH, ICH+Exo treated and sham rat brains (named ICH group, ICH+Exo group, and Sham group, respectively), brain tissue samples were collected from rats at one-millimeter intervals before and after the hematoma area along the coronal plane for subsequent standardized time-course (1, 3, 7, and 14 dpi) for ST analysis, standardized time-course (1, 3, 7, 14, 21, and 28 dpi) for scRNA-seq analysis. (B) The precise injection site of collagenase during the establishment of a rat ICH model. (C) The precise sampling locations of scRNA-seq and ST.

**Intracerebral hemorrhage model and Exosome injection.**

The rats were anesthetized using isoflurane gas anesthesia and were fixed in the stereotaxic apparatus after removing the head hair. After disinfection, the skin was incised, the periosteum was exposed, and a hole was drilled 0.1 mm behind the sagittal suture and 3 mm to the right of the midline. Then, 2 µL of collagenase IV (0.25 U/µL) was extracted from the vial, and the needle was injected slowly to a distance of 5.5 mm. The needle was left in place for 10 min, bone wax was used to close the hole, and the wound was sutured and disinfected again.

At 48 hours post-ICH, we assessed the model using the mNSS scoring system and selected rats with mNSS scores exceeding 10 for subsequent experiments. Then intrathecally injected with 2×10^10^ particles/rats Exo along the original hole. To reduce the impact of the hematoma areas effect of Exo, we selected the injection site at a depth of 4.5 mm. The sham group only punctured the brain tissue with a microsyringe without collagenase injection, while the ICH group was injected with saline instead of Exo at 48 hours post-ICH.

**Exosomes information**

Exosomes from human bone marrow-derived mesenchymal stem cells (HQExoA002L3001, Huamei BioTech Co. Ltd., Wuhan, China). Exo identification and absorption data can be found in Figure S16.

**Figure S3**

**
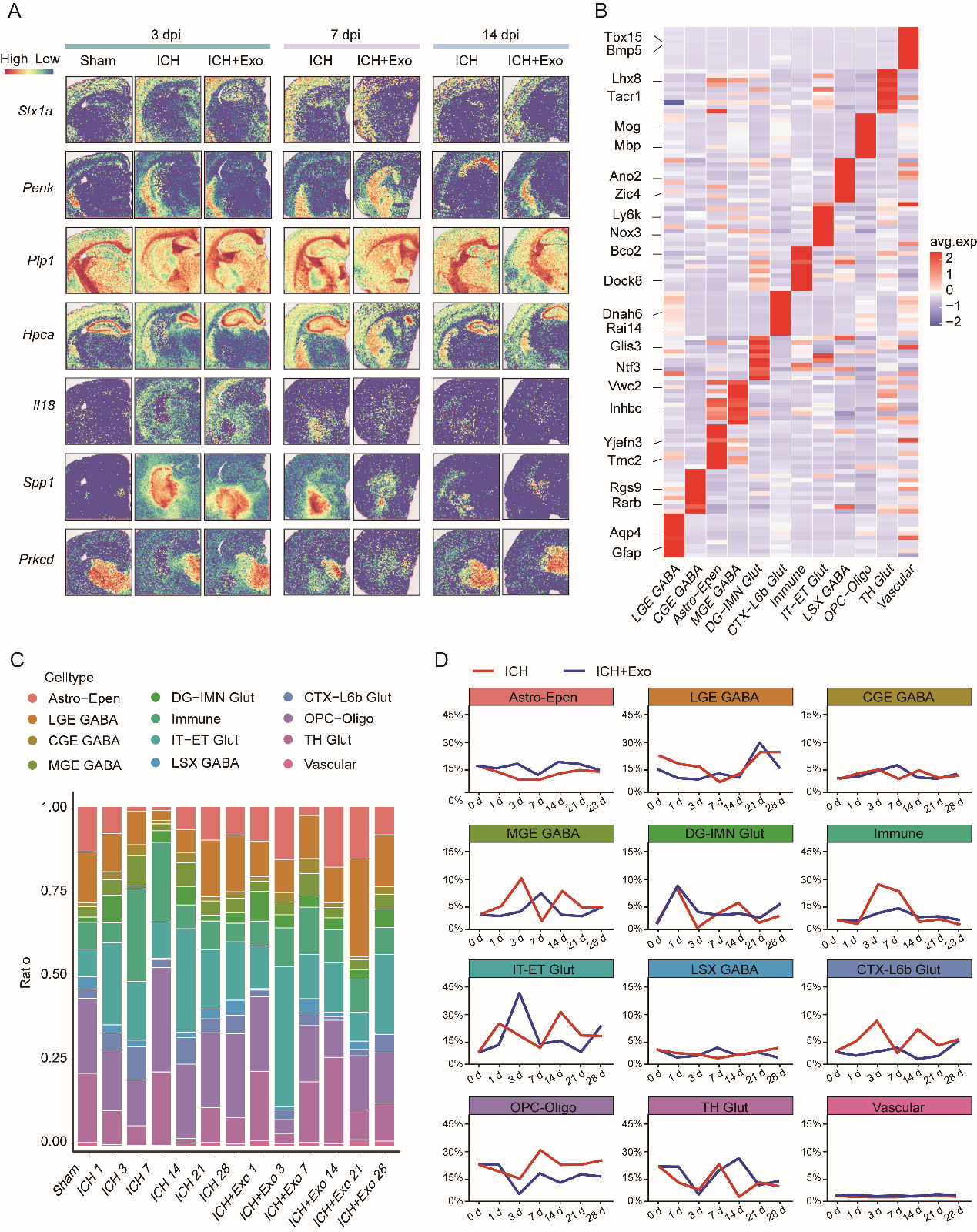
**

**Figure S3** (A) Expression profiles of genes *Stx1a*, *Penk*, *Plp1*, *Hpca*, *Il18*, *Spp1*, and *Prkcd* across the ST slides. (B) The heatmap displays the feature genes of the 12 cell types detected by scRNA-seq. (C) Bar chart depicting the proportion of each cell type at various time points for both ICH and ICH+Exo groups. (D) Line chart represents the changing trends of cell types in the ICH and ICH+Exo groups.

**Figure.S4
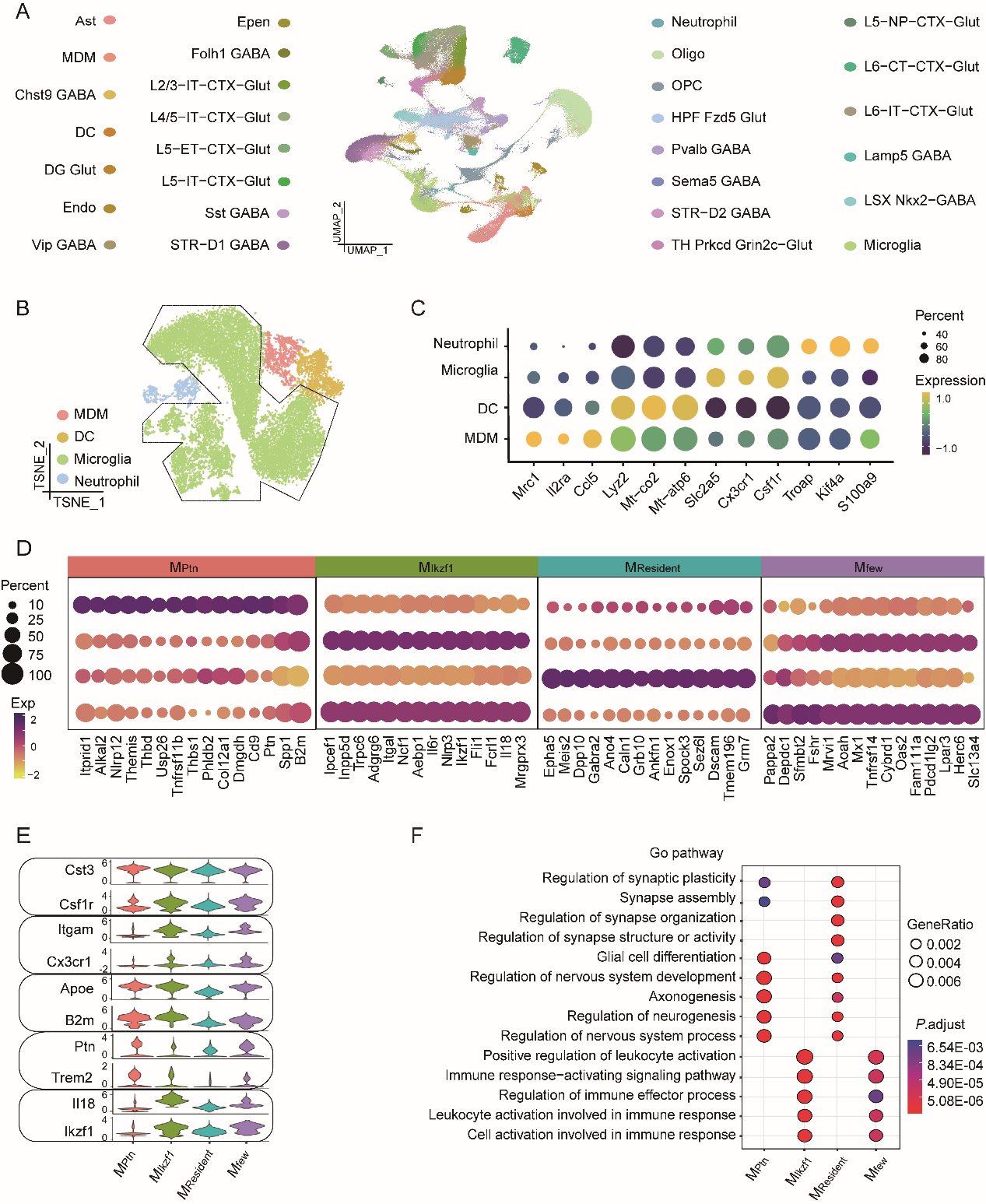
**

**Figure S4** (A) Dimension-reduced single-cell transcriptomic data were visualized through UMAP. Cells identified into 29 subtypes, including Ast (astrocyte), MDM (macrophages derived from monocytes), Chast9 GABA (chst9 GABAergic neurocyte), DC (Dendritic cells), DG Glut (dentate gyrus glutamatergic neurocyte), Endo ( endothelial cells), Epen (ependymal), Folh1 GABA (folh1 GABAergic neurocyte), L2/3-IT-CTX-Glut (layer 2/3, intratelencephalic, cerebral cortex, glutamatergic neurocyte), L4/5-IT-CTX-Glut (layer 4/5, intratelencephalic, cerebral cortex, glutamatergic neurocyte), L5-ET-CTX-Glut (layer 5, extratelencephalic, cerebral cortex, glutamatergic neurocyte), L5-IT-CTX-Glut (layer 5, intratelencephalic, cerebral cortex, glutamatergic neurocyte), L5-NP-CTX-Glut (layer 5, near-projecting, cerebral cortex, glutamatergic neurocyte), L6-CT-CTX-Glut (layer 6, corticothalamic, cerebral cortex, glutamatergic neurocyte), L6-IT-CTX-Glut (layer 6, intratelencephalic, cerebral cortex, glutamatergic neurocyte), Lamp5 GABA (lamp5 GABAergic neurocyte), LSX Nkx2-GABA (lateral septal complex nkx2, GABAergic neurocyte), Microglia, Neutrophil, Oligo (oligodendrocytes), OPC (oligodendrocyte precursor cells), HPF Fzd5 Glut (hippocampal formation glutamatergic neurocyte), Pvalb GABA (pvalb GABAergic neurocyte), Sema5 GABA (sema5 GABAergic neurocyte), Sst GABA (sst GABAergic neurocyte), STR-D1 GABA (striatum, dorsal 1 GABAergic neurocyte), STR-D2 GABA (striatum, dorsal 2 GABAergic neurocyte), TH Prkcd Grin2c-Gult (thalamus prkdc grin2c, glutamatergic neurocyte), and Vip GABA (vip GABAergic neurocyte). (B) TSNE plot showing four immune. (C) Dotplot of normalized mean expression of signature genes for each subtype. (D) The bubble chart displays the feature genes of four subclasses of microglia. (E) Violin chat of normalized mean expression of homeostatic genes of microglia and disease-associated microglia (DAM) genes for four subclasses of microglia. (F) Representative GO biological processes pathway activities scored per subclasses by GSEA.

**Figure S5**

**
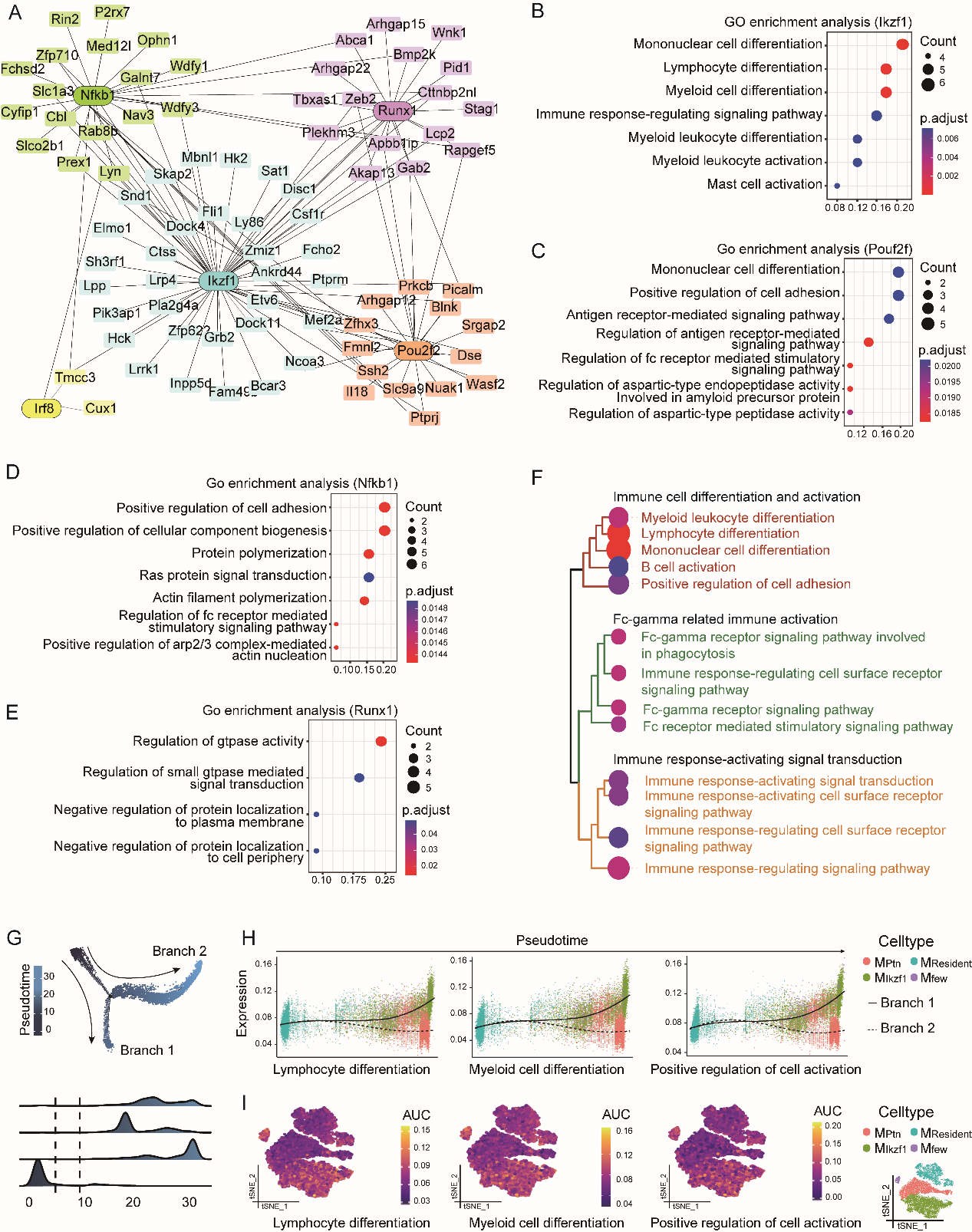
**

**Figure S5** (A) Gene regulatory network (GRN) diagram for the Ikzf1, illustrating interactions between key regulators and high-scoring target genes. (B) Enriched GO pathways (biological processes) of *Ikzf1*, *Pouf2f* (C), *Nfkb1* (D), and *Runx1* (E) transcripts and their highly active target genes through GSEA. (F) GSEA of representative GO biological processes for high-scoring target genes for the Ikzf1. (G) Developmental trajectory of microglia visualized using Monocle 2, with coloring based on pseudotime scoring. (H) Monocle 2-derived fitting curve illustrating expression trends of specific pathways in branch 1. (I) Area under the curve (AUC) scores for specific pathways displayed on a tSNE plot of microglia.

**Figure S6**


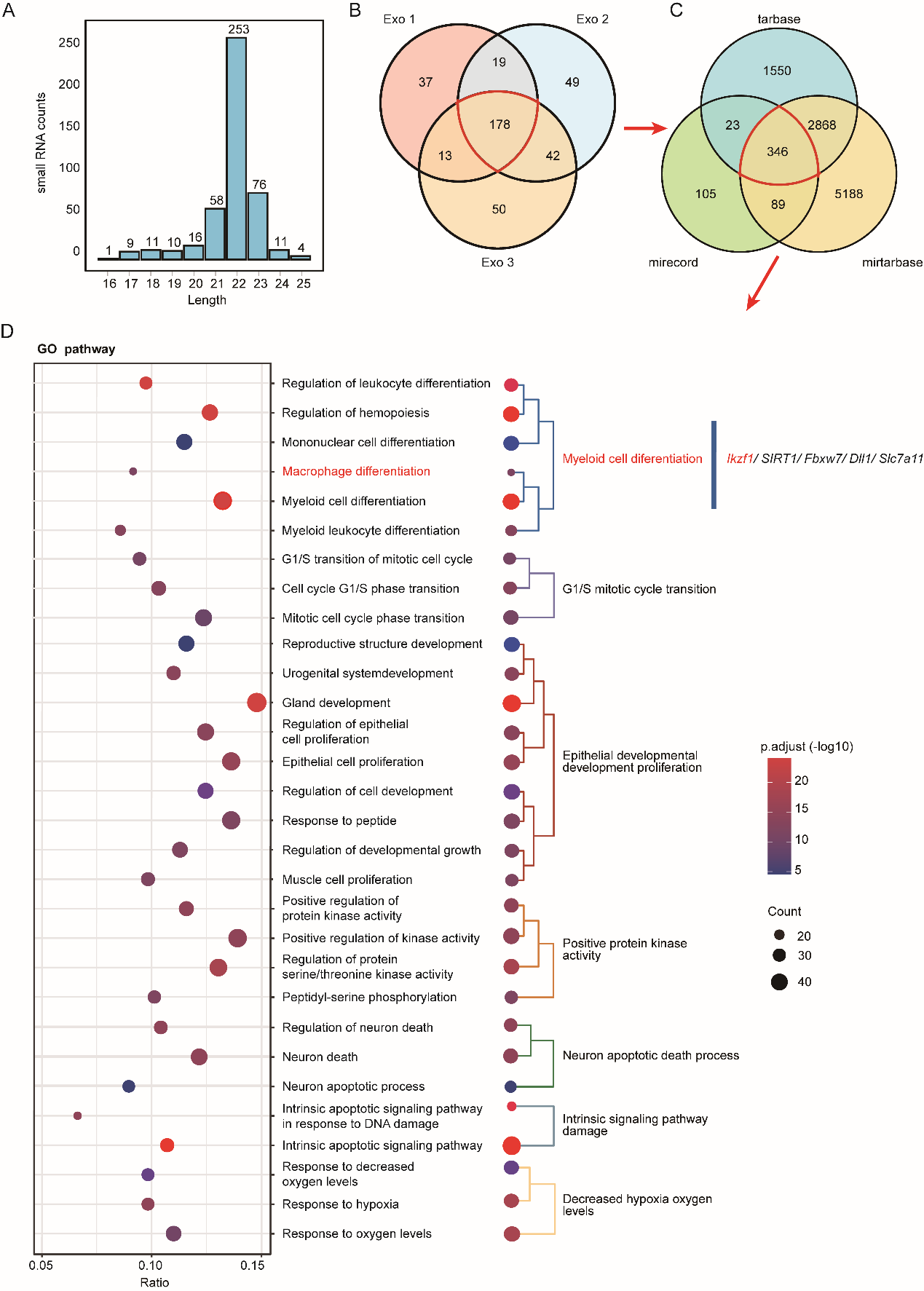


**Figure S6** (A) The bar chart represents the distribution of small RNA lengths. (B) Venn diagram of 178 miRNAs consistently expressed in three validations. (C) Ven chart showing 346 co-expression of robust prediction target intersections in the tarbase, mirecord, and mirtarbase databases. (D) Representative GO (biological processes) pathway terms in the target genes by GSEA. The significance threshold was set to an adjusted P-value < 0.05.

**Figure S7**


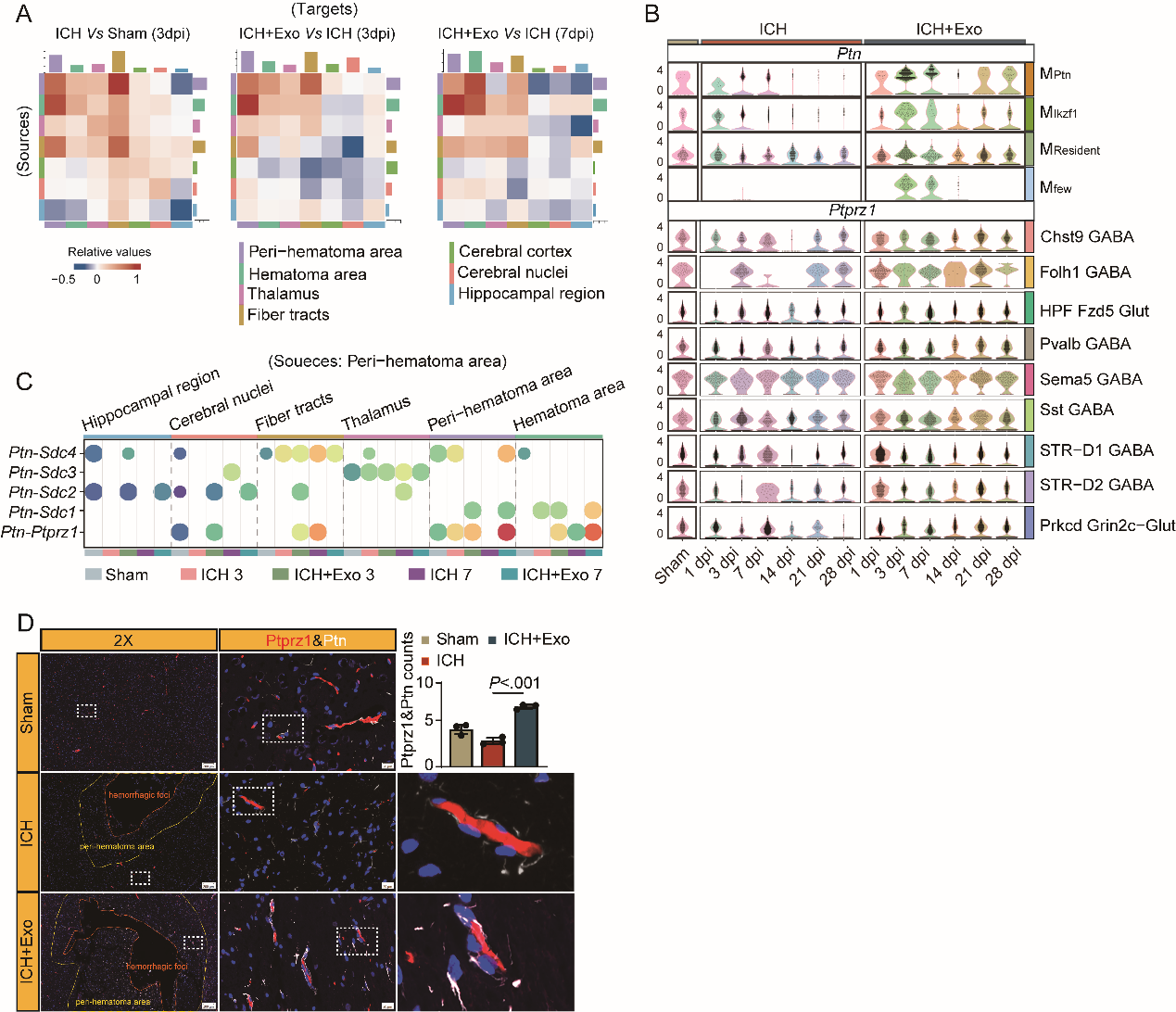


**Figure S7** (A) Heatmap showing changes in cell communication weights across different regions within the ST in three experimental conditions: ICH *vs*. Sham at 3 dpi, ICH+Exo *vs*. ICH at 3 dpi, and ICH+Exo *vs*. ICH at 7 dpi. (B) Bubble chart depicting the strength of signal transduction in the peri-hematoma area and other regions, across all subtypes of the PTN pathway. (C) Expression of Ptprz1 in Chst9 GABA, Folh1 GABA, HPF Fzd5 Glut, Sema5 GABA, Sst GABA, STR-D1 GABA, STR-D2 GABA, and Th Prkcd Grin2c-Glut and Ptn in microglia subclasses. (D) Double immunostaining of Ptn and Ptprz1 in the peri-hematoma area with quantitative analysis in Sham, ICH, and ICH+Exo groups (3dpi), n=3 rats per group.

**Figure S8**


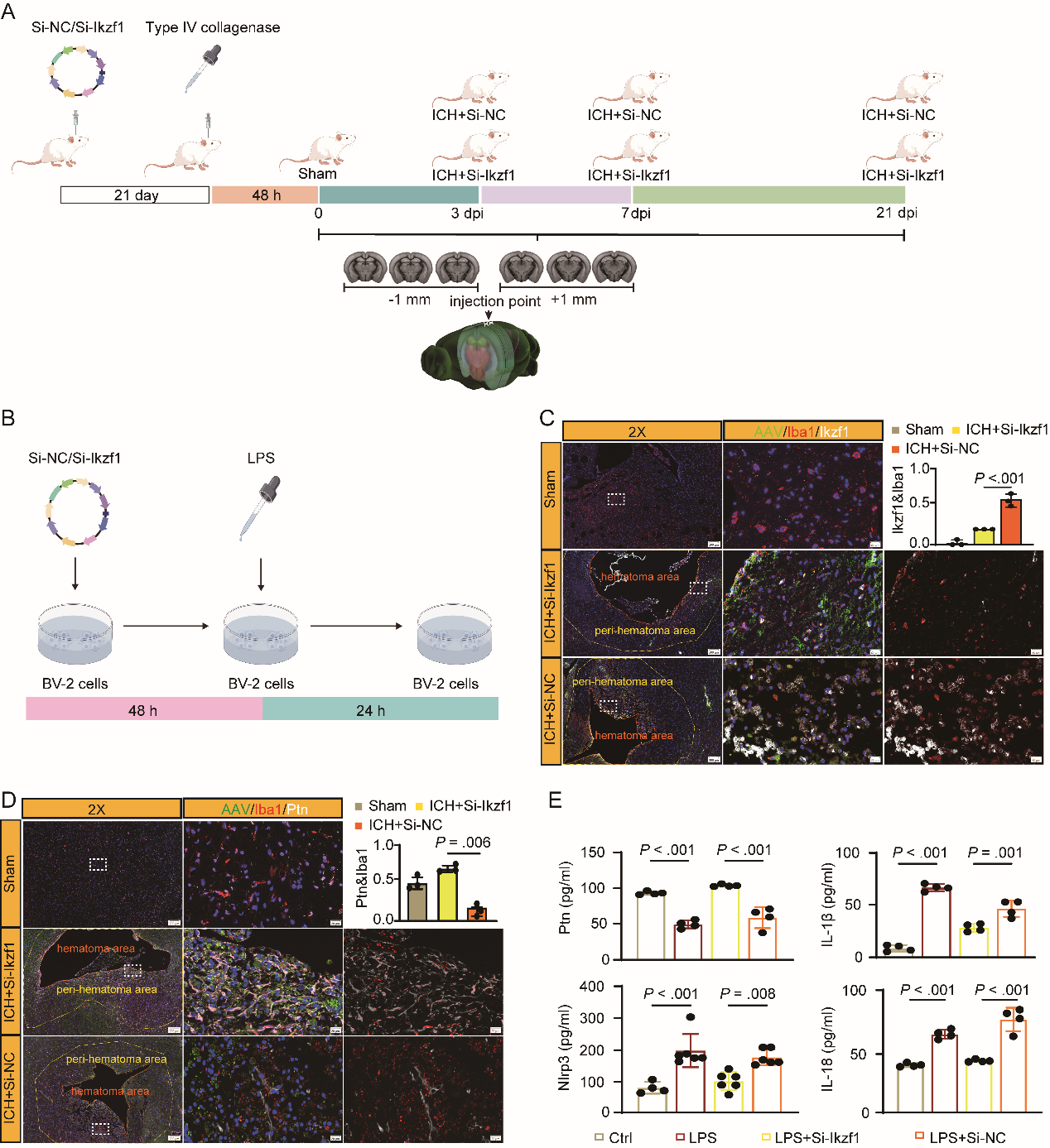


**Figure S8** (A) The procedure of infecting rat brains with adenovirus and establish a model of ICH. We employed adenovirus to specifically silence the *Ikzf1* in rat brain microglia (ICH+Si-Ikzf1 group), utilizing blank vector virus as a control (ICH+Si-NC group), virus information can be seen in Table. S12. Injecting adenovirus into the rat’s brain 21 days in advance, followed by type IV collagenase injection, brain tissue samples were collected from rats at one-millimeter intervals before and after the hematoma area along the coronal plane for subsequent standardized time-course (1, 3, 7, and 14 dpi) for ST analysis, standardized time-course (1, 3, 7, 14, 21, and 28 dpi) for scRNA-seq analysis. (B) Infect Bv-2 cells with lentivirus 48 hours prior to intervention, followed by cell collection 24 hours post LPS stimulation. (C) Double immunostaining of Ikzf1 and Iba1 in the peri-hematoma area in 3 dpi, n=3 rats per group. (D) Double immunostaining of Ptn and Iba1 in the peri-hematoma area regions and quantitative analysis in 3 dpi, n=3 rats per group. (E) Protein expression of Ptn, IL-1β, Nlrp3 and IL-18 in BV-2 cell was detected using ELISA assay, n≥4 per group.

**Figure S9**


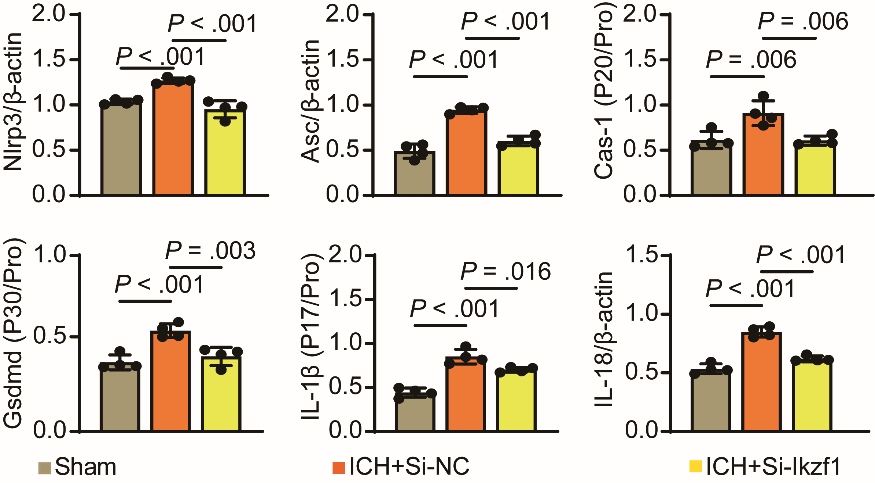


**Figure S9** Bar charts for figure.3 J indicate the ratios of Nlrp3/β-actin, Asc/β-actin, caspase-1 (P20/Pro), IL-1β (P17/Pro), IL-18/β-actin and Gsdmd (P30/Pro), n=4 per group. (H) Protein expression of brains of Sham, ICH, ICH+Si-Ikzf1, and ICH+Si-NC group (3 dpi) was detected using Western blot. The original Western blot images are presented in Figure S24, where the protein molecular weight markers were labeled.

**Figure S10**


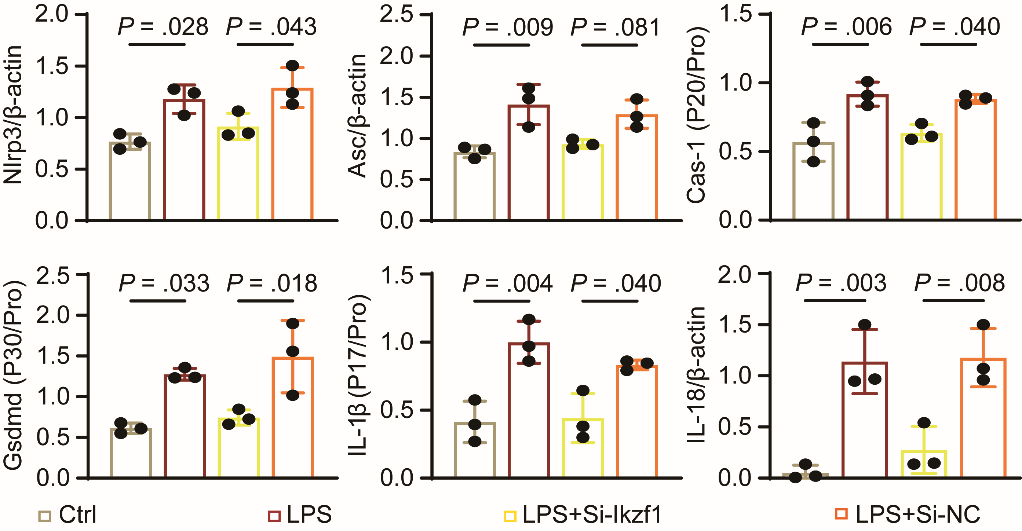


**Figure S10** Bar charts for figure.3 K indicate the ratios of Nlrp3/β-actin, Asc/β-actin, caspase-1 (P20/Pro), IL-1β (P17/Pro), IL-18/β-actin and Gsdmd (P30/Pro), n=4 per group. (H) Protein expression of Bv-2 cells of Ctrl, LPS, LPS+Si-Ikzf1, and LPS+Si-NC group was detected using Western blot. The original Western blot images are presented in Figure S24, where the protein molecular weight markers were labeled.

**Figure S11**

**
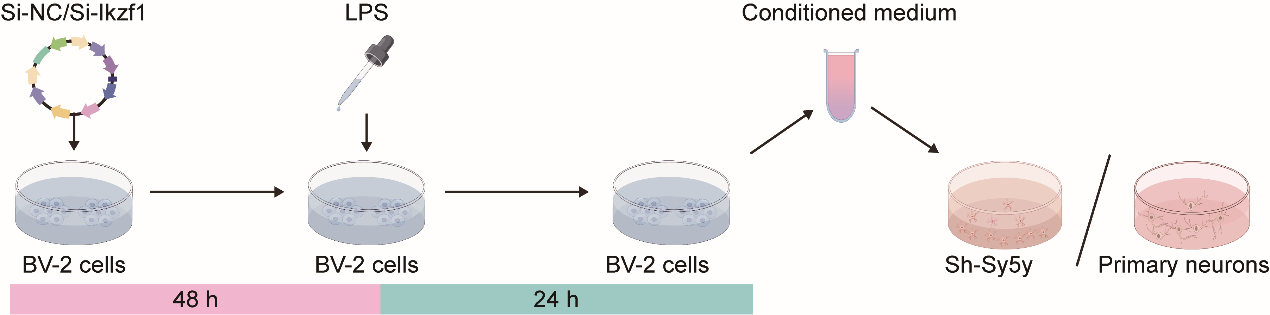
**

**Figure S11** The experimental protocol includes infecting BV-2 cells with lentivirus and harvesting conditioned medium for application in interventions with Sh-Sy5y cells and primary mouse cells. BV-2 cells were exposed to lentivirus 48 hours prior to the intervention, while conditioned medium collection occurred 24 hours post LPS stimulation for subsequent interventions with Sh-Sy5y cells and primary mouse cells.

**Figure S12**

**
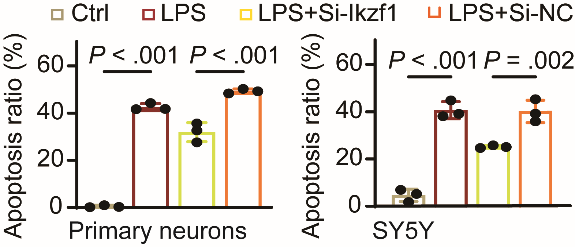
**

**Figure S12** Bar charts for figure.4C display the apoptosis ratio, n=3 per group.

**Figure S13**

**
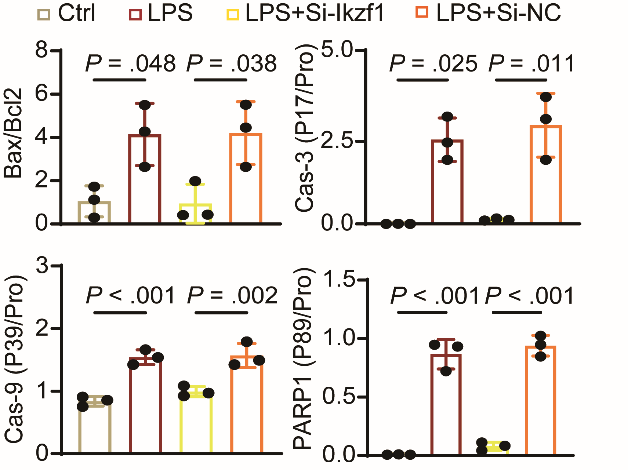
**

**Figure S13** Bar charts for figure.4E indicate the ratios of Bax/Bcl2, Capase-3 (P17)/ Capase-3(Pro), Capase-9 (P39)/ Capase-9 (Pro) and PARP1 (P89)/ PARP1 (Pro) in Sh-Sy5y cells, n=3 per group.

**Figure S14**

**
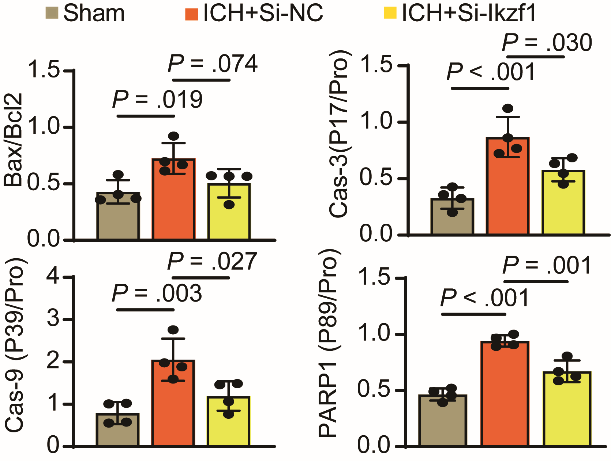
**

**Figure S14** Bar charts for figure.4F indicate the ratios of Bax/Bcl2, Capase-3 (P17)/ Capase-3(Pro), Capase-9 (P39)/ Capase-9 (Pro) and PARP1 (P89)/ PARP1 (Pro) in BV-2 cells, n=4 rats per group.

**Figure.S15**

**
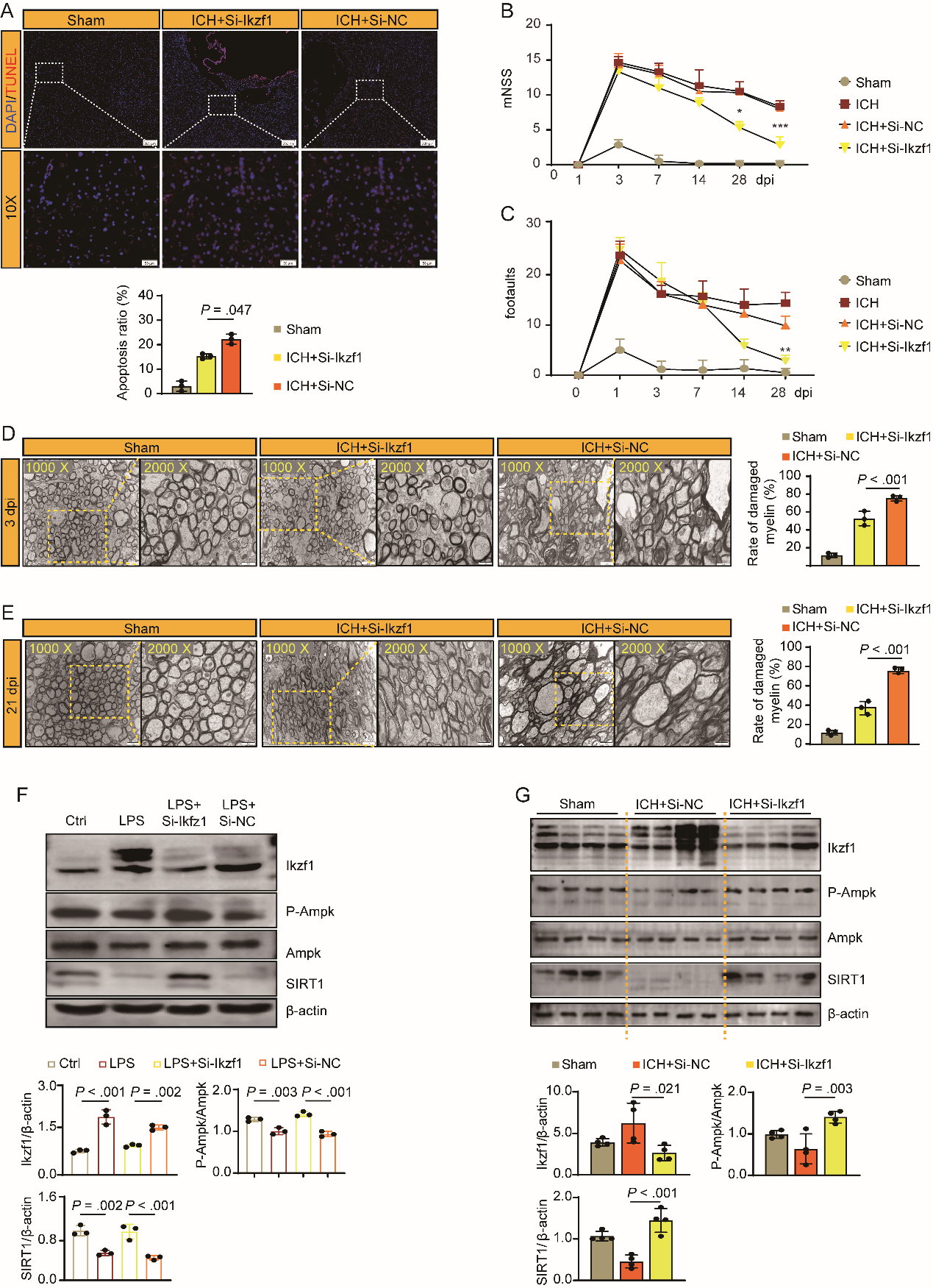
**

**Figure.S15** (A) TUNEL staining of Sham, ICH+Si-NC, and ICH+Si-Ikzf1 group (3 dpi). Bar charts indicate the PI/Hoechst ratio. (B) mNss was assessed in ICH rat at 1–28 dpi, n≥5 rats per group. (C) Sensorimotor dysfunction in ICH rats at 1–28 dpi was assessed by foot-fault test, n≥5 rats per group. (D) TEM images illustrating the myelin sheath integrity in Sham, ICH+Si-Ikzf1, and ICH+Si-NC group. Bar charts showing the percentage of damaged myelin (3 dpi), n=3 rats per group. (E) TEM images illustrating the myelin sheath integrity in Sham, ICH+Si-Ikzf1, and ICH+Si-NC group. Bar charts showing the percentage of damaged myelin (21 dpi), n=3 rats per group. (F) Western blot analysis of protein expression in Ctrl, LPS, LPS+Si-Ikzf1, and LPS+Si-NC group. Bar charts showing the expression ratios of Ikzf1/β-actin, P-Ampk/Ampk, and SIRT1/β-actin in BV-2 cells, n=3 per group. The original Western blot images are presented in Figure.S22, where the protein molecular weight markers were labeled. (G) Western blot analysis of brain protein expression in Sham, ICH+Si-Ikzf1, and ICH+Si-NC group. Bar charts showing the expression ratios of Ikzf1/β-actin, P-Ampk/Ampk, and SIRT1/β-actin in ICH rats (3 dpi), n=4 rats per group. The original Western blot images are presented in Figure.S23, where the protein molecular weight markers were labeled.

**Figure S16**


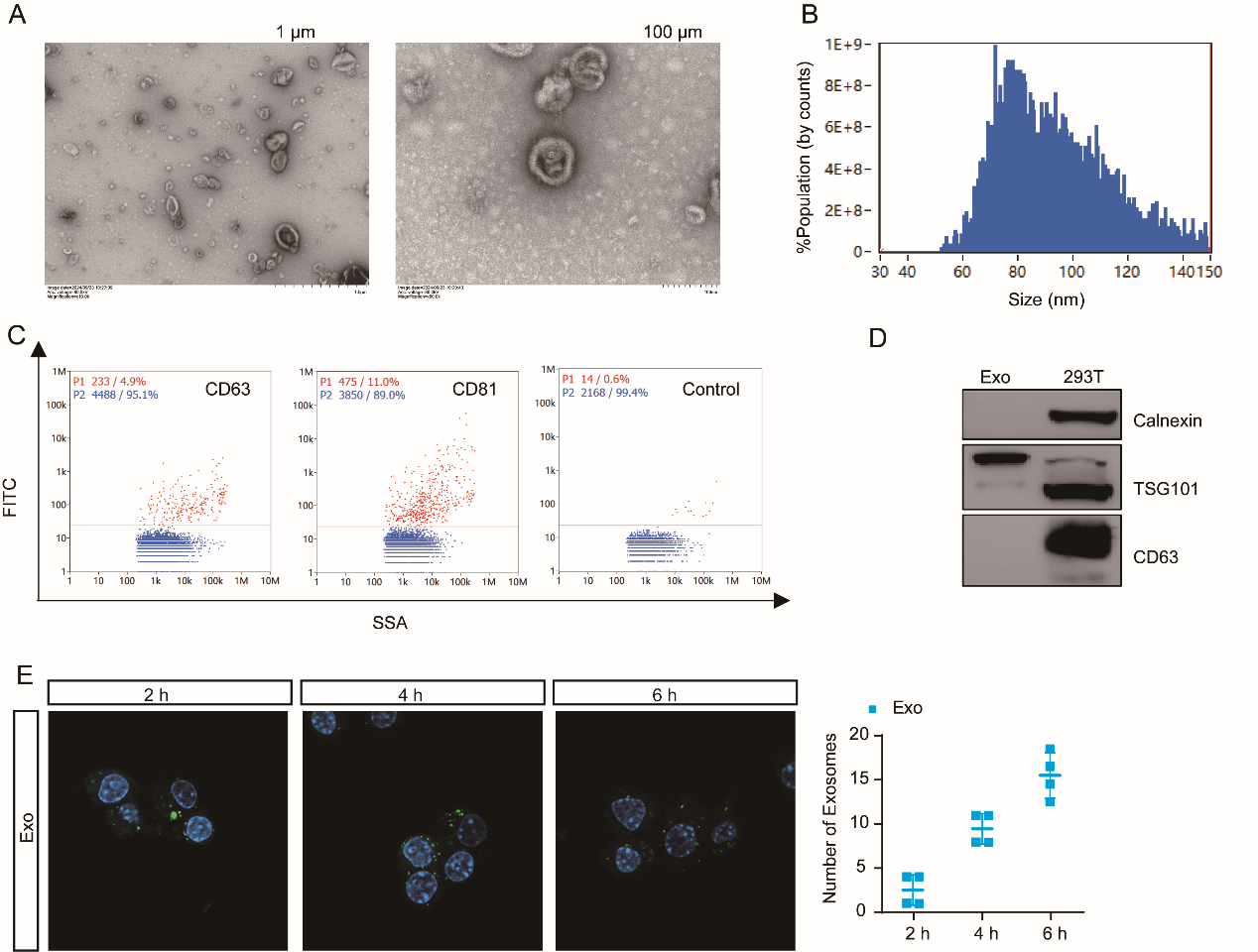


**Figure S16.** (A) Representative TEM of Exo. (B) Schematic diagram of Exo particle size. (C) Flow cytometry technique detect the expression of Exo marker protein. (D) Western blot was used to detect the expression of Exo marker protein. (E) The images of Exo uptake by Bv-2 cells. Scatter plots illustrate the uptake of Exo by Bv-2 cells at different time points (2h, 4h, 6h), n=6 per group.

**Figure S17.**


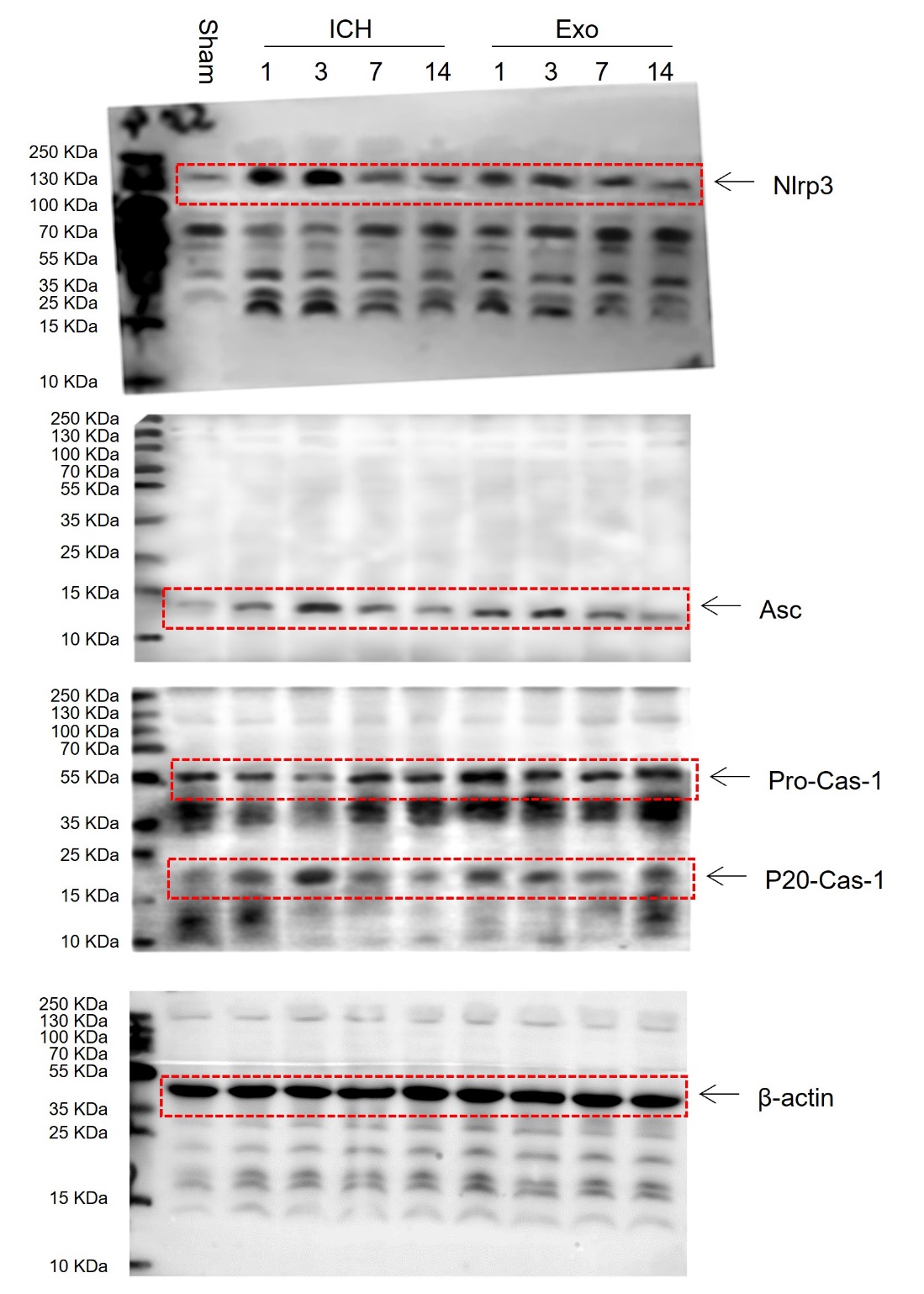


**Figure S17.** The original Western blot, where the protein molecular weight markers were labeled.

**Figure S18.**

**
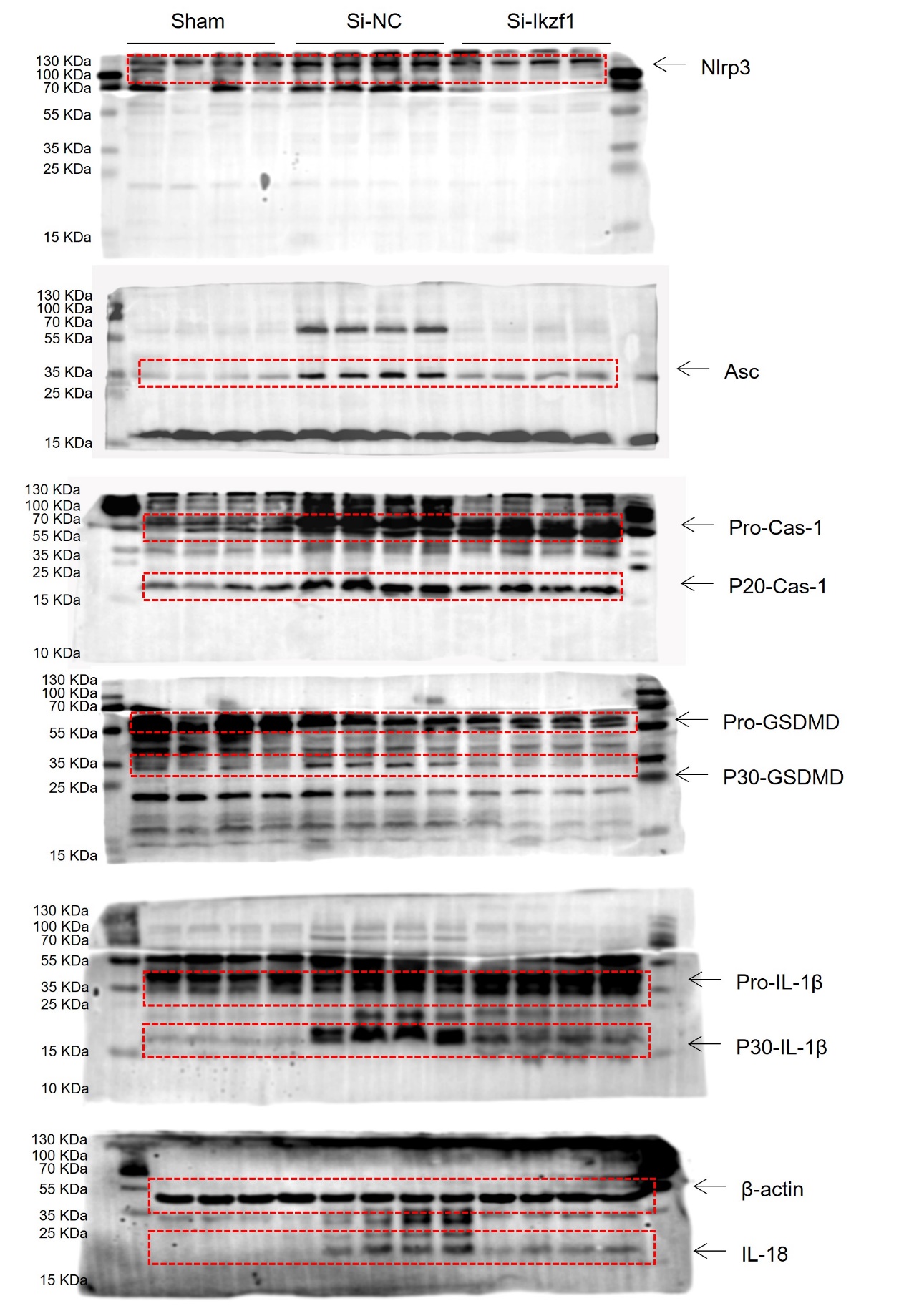
**

**Figure S18.** The original Western blot, where the protein molecular weight markers were labeled.

**Figure S19.**


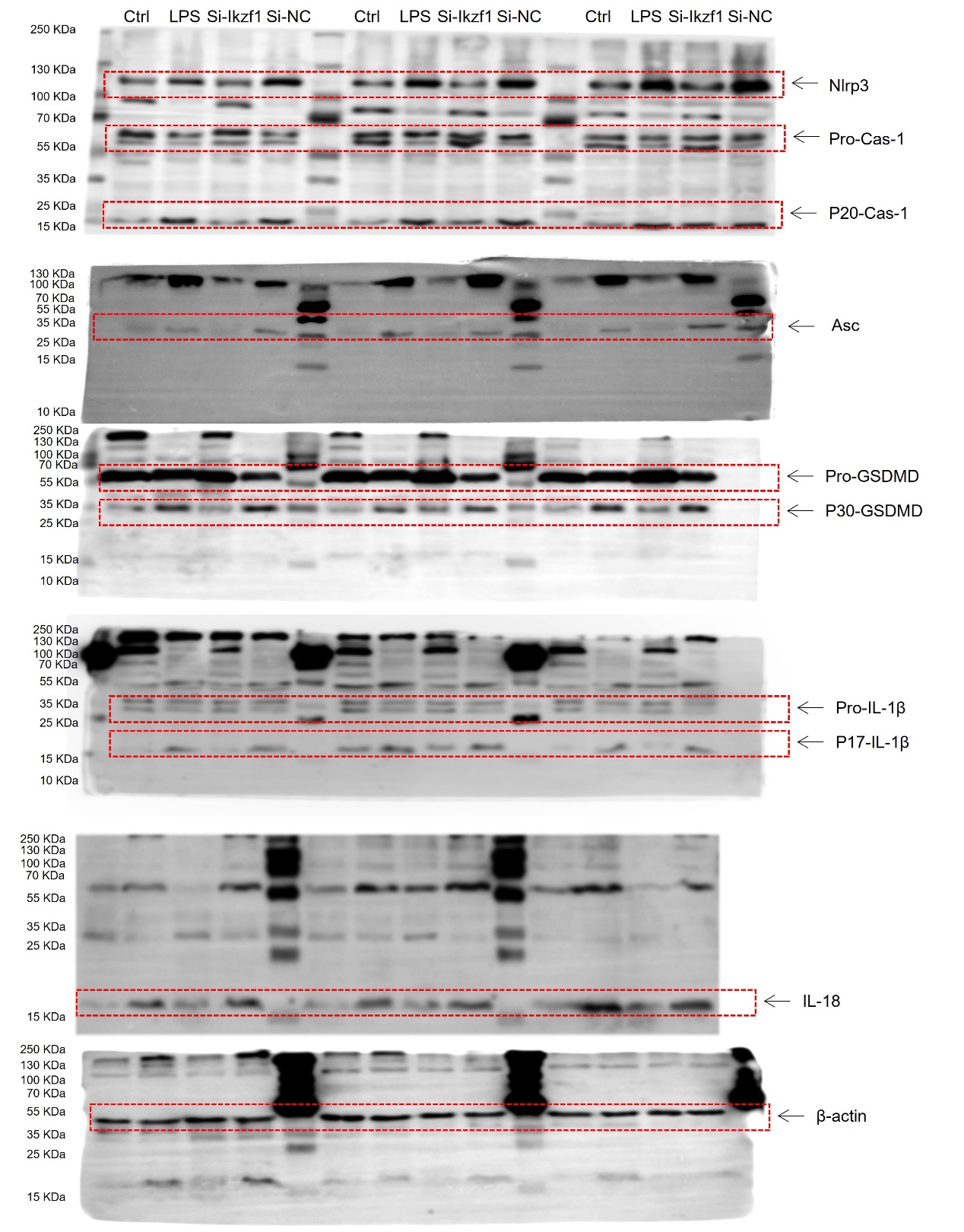


**Figure S19.** The original Western blot, where the protein molecular weight markers were labeled.

**Figure S20**

**
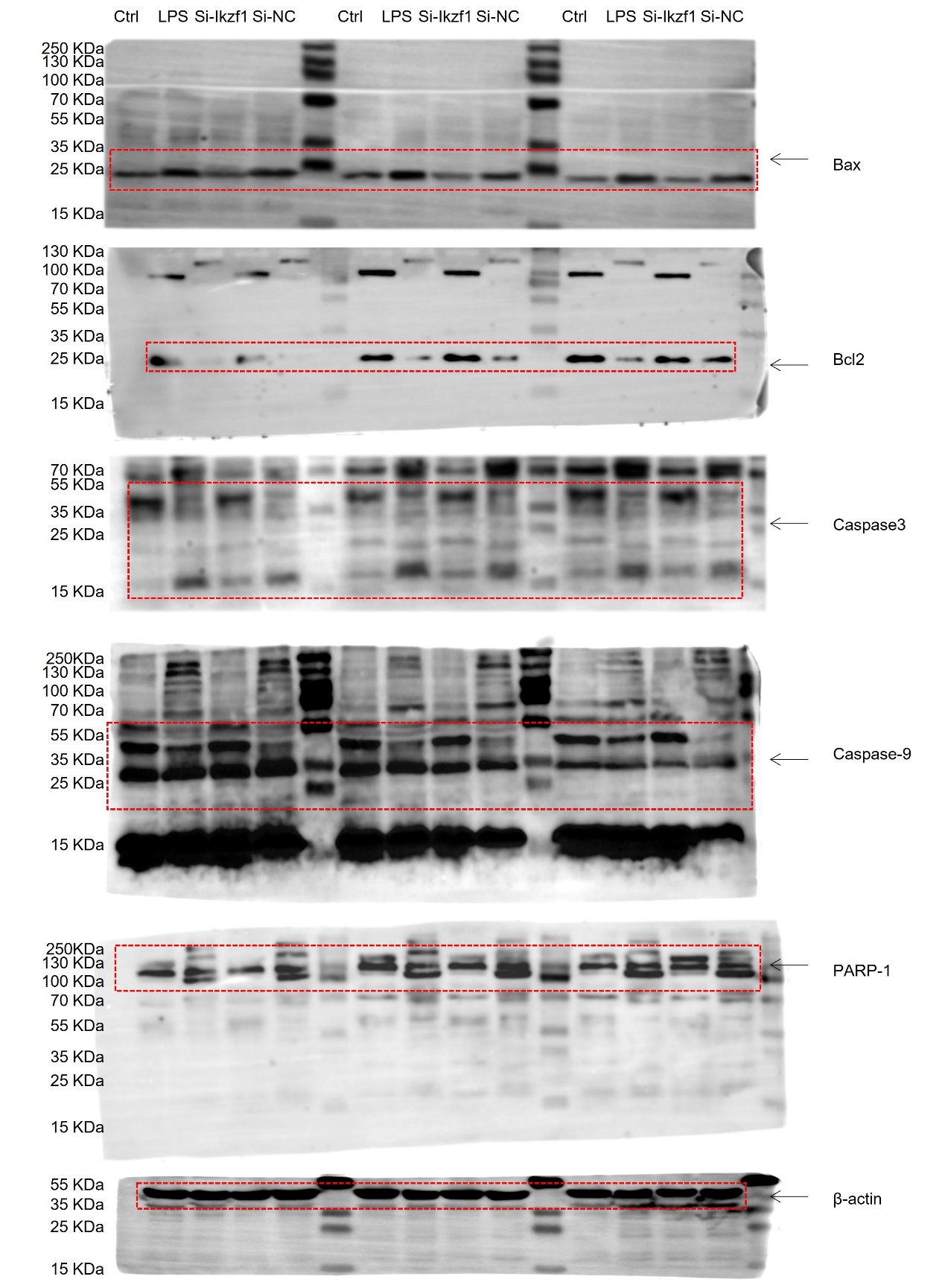
**

**Figure S20.** The original Western blot, where the protein molecular weight markers were labeled.

**Figure S21.**

**
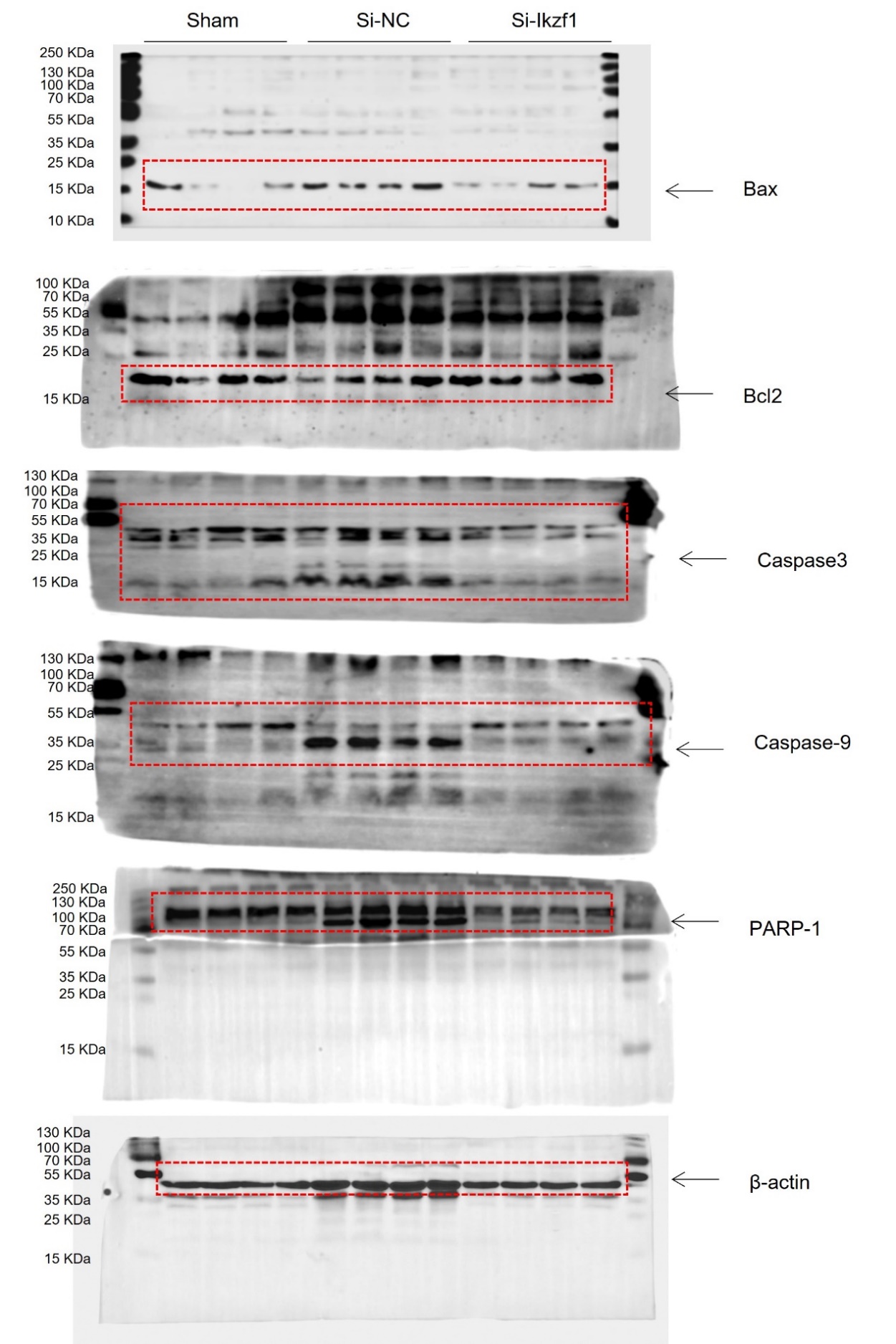
**

**Figure S21.** The original Western blot, where the protein molecular weight markers were labeled.

**Figure S22.**

**
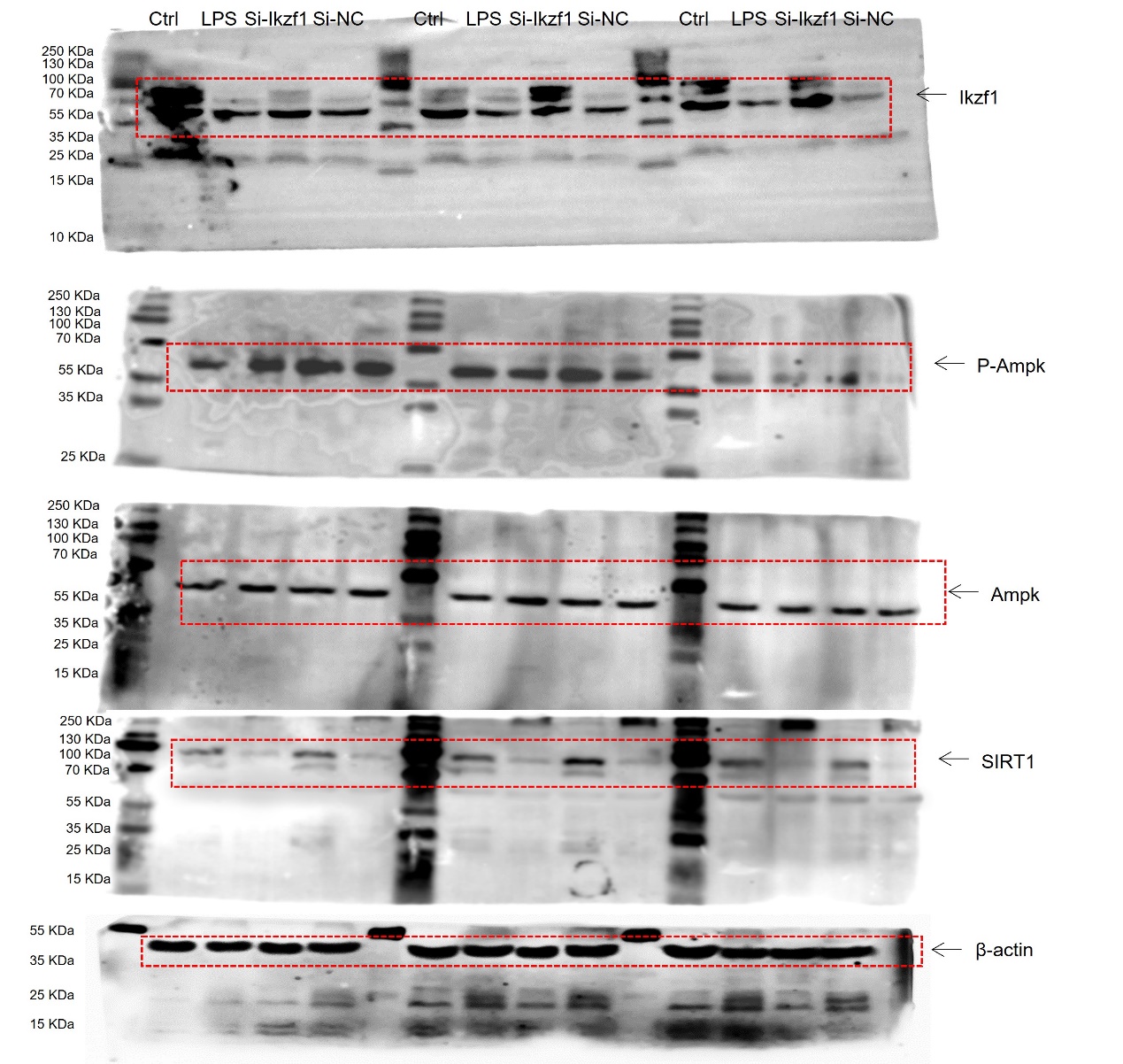
**

**Figure S22.** The original Western blot, where the protein molecular weight markers were labeled.

**Figure S23.**

**
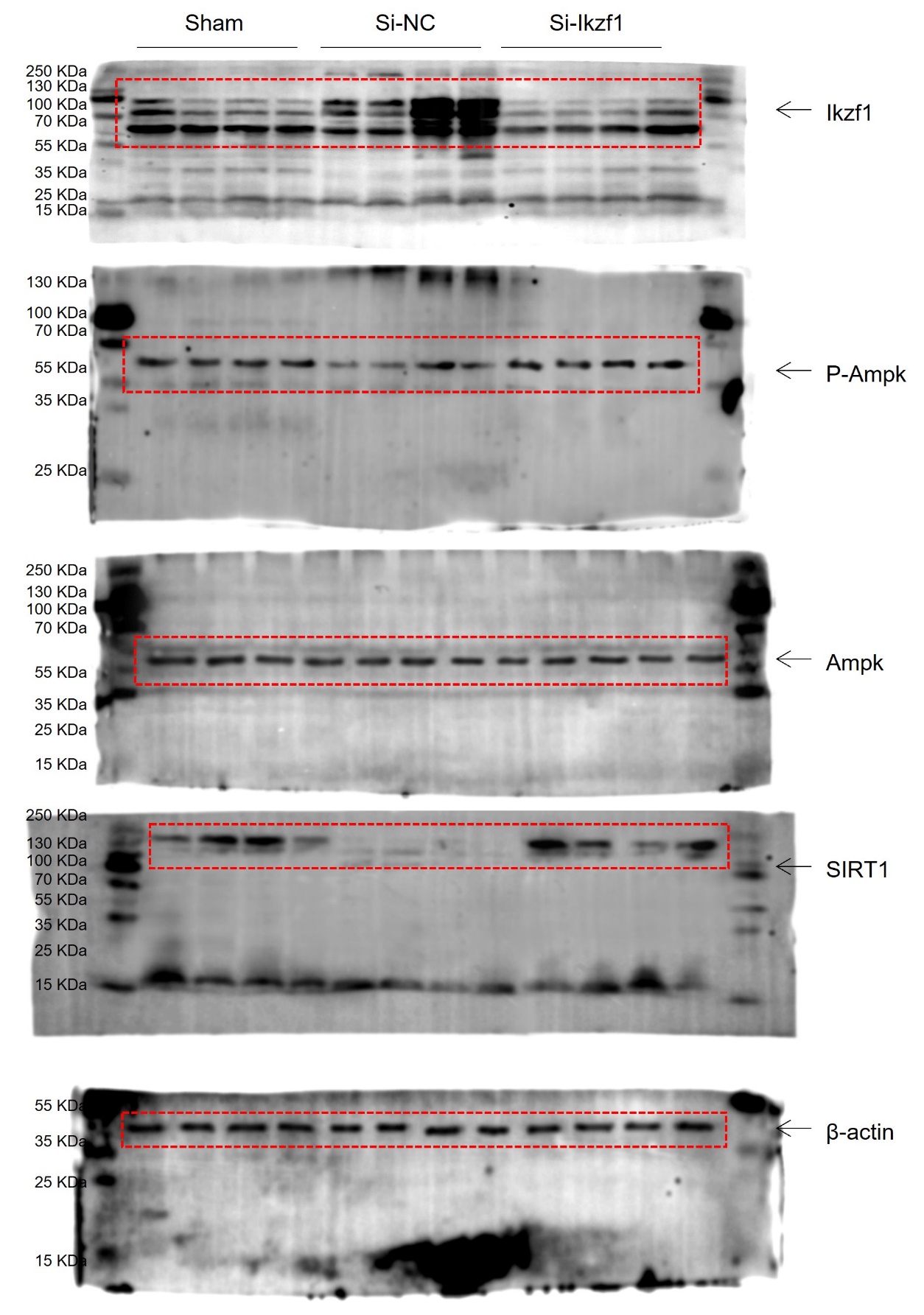
**

**Figure S23.** The original Western blot, where the protein molecular weight markers were labeled.

# Supplementary Tables

**Table S1**. Table of GSEA KEGG enrichment results for specific genes in the peri-hematoma area region of different groups of ST data.

| Pathways | Sham | ICH 3 | Exo 3 | ICH 7 | Exo 7 | ICH 14 | Exo 14 |
| --- | --- | --- | --- | --- | --- | --- | --- |
| Wnt signaling pathway | 0.1873 | -0.3084 | -0.3546 | 0.0008 | -0.1294 | 0.0617 | 0.2048 |
| Long term_depression | -0.0405 | -0.2614 | -0.2275 | -0.0507 | 0.0812 | -0.2798 | 0.1677 |
| Axon guidance | 0.1843 | -0.1884 | -0.3168 | 0.0020 | 0.1742 | -0.1303 | 0.0379 |
| Arachidonic acid metabolism | -0.1068 | 0.3138 | 0.2373 | -0.1801 | 0.0236 | 0.1906 | -0.3289 |
| Fc epsilon RI signaling pathway | -0.2487 | 0.1774 | 0.0755 | 0.1342 | 0.0090 | -0.1457 | -0.1553 |
| B cell receptor signaling pathway | -0.4100 | 0.2040 | 0.1415 | 0.1913 | 0.2816 | -0.1232 | -0.1868 |
| T cell receptor signaling pathway | -0.3885 | 0.0592 | -0.0284 | 0.1963 | 0.2385 | -0.0320 | -0.1375 |
| Toll like receptor signaling pathway | -0.4828 | 0.2065 | 0.0940 | 0.3476 | 0.1791 | 0.0819 | -0.1082 |
| Apoptosis | -0.4050 | 0.3541 | 0.2082 | 0.2582 | 0.0842 | -0.1649 | -0.2647 |
| NOD-like receptor signaling pathway | -0.5472 | 0.2166 | 0.1558 | 0.2020 | 0.1080 | -0.0329 | -0.1059 |

**Table S2**. Canonical marker genes used to identify cell-types

| Cell-type | Gene |
| --- | --- |
| Astro-Epen | *Itih3, Kcnk16, Fmo2, Etnppl, Aox1, Gfap, Aqp4, Grin2c, Slco1c1, Maob* |
| CNU-LGE GABA | *Rgs9, Sh3rf2, Meox2, Il20ra, Dach1, Syndig1l, Itgb6, Rarb, Strip2, Foxp2* |
| CTX-CGE GABA | *Tmc2, Yjefn3, Adarb2, Cps1, Egfr, Tp63, Pde11a, Frem1, Pde5a, Ngf* |
| CTX-MGE GABA | *Inhbc, Kcnc2, Kcnmb2, Lypd6b, Vwc2, Kcnip1, Mybpc1, Adamts15, Btbd11, Pparg* |
| DG-IMN Glut | *Ntf3, Glis3, Palmd, Ptpn14, Syt17, Vwa3a, Ccdc27, Il16, Pla2g4f, Kcnj6* |
| Immune | *Bco2, Dock8, Fyb1, Pik3ap1, Ptprc, Slfn4, Stab1, Aoah, Blnk, Pik3cg* |
| IT-ET Glut | *Xirp2, Cyp11b3, Ly6k, Clca4, Nox3, Clca4l, Satb2, Tacr3, Trim54, Hnmt* |
| LSX GABA | *Ano2, Crhr2, Ano1, Nox4, Gnat3, Myo5b, Grid2ip, Zic4, Trpc4, Dgkg* |
| NP-CTX-L6b Glut | *Trabd2b, Chrna5, Rai14, Dnah6, Adamts18, Zfpm2, Tnfsf8, Dmgdh, Itga11, Clic5* |
| OPC-Oligo | *Hapln2, Bcas1, Mobp, Mag, Mbp, Mog, Rpe65, Cdh19, Plxnb3, Gpd1* |
| TH Glut | *Slc17a8, Lhx8, Insrr, Stk32b, Tacr1, Chat, Ndst4, Dgkk, Upp2, Chodl* |
| Vascular | *Slc6a13, Abcc9, Tbx15, Prrx2, Akr1d1, Foxd1, Atp13a5, Rgs5, Bmp5, C7* |

**Table S3**. Table of GSEA KEGG enrichment results of specific genes in different groups of scRNA-seq data.

| Group | Description | GeneRatio | p.adjust | Count |
| --- | --- | --- | --- | --- |
| Saline | Neuroactive ligand-receptor interaction | 6/19 | 0.008703 | 6 |
| ICH 7 | Cell adhesion molecules | 9/111 | 0.005177 | 9 |
| ICH 7 | Fc gamma R-mediated phagocytosis | 9/111 | 0.000290 | 9 |
| ICH 7 | Osteoclast differentiation | 9/111 | 0.002471 | 9 |
| ICH 7 | Platelet activation | 10/111 | 0.000290 | 10 |
| ICH 7 | Rap1 signaling pathway | 12/111 | 0.000829 | 12 |
| ICH 3 | Cell adhesion molecules | 8/115 | 0.019237 | 8 |
| ICH 3 | Fc gamma R-mediated phagocytosis | 9/115 | 0.000548 | 9 |
| ICH 3 | Osteoclast differentiation | 11/115 | 0.000347 | 11 |
| ICH 3 | Platelet activation | 9/115 | 0.002621 | 9 |
| ICH 3 | Rap1 signaling pathway | 11/115 | 0.004214 | 11 |
| ICH 14 | Glutamatergic synapse | 7/60 | 0.001978 | 7 |
| ICH+Exo 7 | Neuroactive ligand-receptor interaction | 5/10 | 0.000908 | 5 |
| ICH+Exo 3 | Aldosterone synthesis and secretion | 7/46 | 0.000040 | 7 |
| ICH+Exo 3 | Axon guidance | 6/46 | 0.004029 | 6 |
| ICH+Exo 3 | cAMP signaling pathway | 7/46 | 0.002165 | 7 |
| ICH+Exo 3 | GABAergic synapse | 4/46 | 0.009690 | 4 |
| ICH+Exo 3 | Glutamatergic synapse | 6/46 | 0.000639 | 6 |
| ICH+Exo 3 | GnRH secretion | 7/46 | 0.000005 | 7 |
| ICH+Exo 3 | Morphine addiction | 6/46 | 0.000250 | 6 |
| ICH+Exo 3 | Neuroactive ligand-receptor interaction | 7/46 | 0.019234 | 7 |
| ICH+Exo 3 | Retrograde endocannabinoid signaling | 8/46 | 0.000040 | 8 |
| ICH+Exo 14 | Aldosterone synthesis and secretion | 5/60 | 0.008669 | 5 |
| ICH+Exo 14 | Arrhythmogenic right ventricular cardiomyopathy | 6/60 | 0.000651 | 6 |
| ICH+Exo 14 | Axon guidance | 9/60 | 0.000370 | 9 |
| ICH+Exo 14 | cAMP signaling pathway | 6/60 | 0.030519 | 6 |
| ICH+Exo 14 | GABAergic synapse | 7/60 | 0.000370 | 7 |
| ICH+Exo 14 | Glutamatergic synapse | 5/60 | 0.012633 | 5 |
| ICH+Exo 14 | GnRH secretion | 4/60 | 0.011203 | 4 |
| ICH+Exo 14 | Morphine addiction | 6/60 | 0.001358 | 6 |
| ICH+Exo 14 | Neuroactive ligand-receptor interaction | 9/60 | 0.011026 | 9 |
| ICH+Exo 14 | Retrograde endocannabinoid signaling | 8/60 | 0.000461 | 8 |

**Table S4**. Table of GSEA GO biological process enrichment results of specific genes in different groups of scRNA-seq data.

| Group | Description | GeneRatio | p.adjust |
| --- | --- | --- | --- |
| Saline | centrosome localization | 3/37 | 0.008884801 |
| Saline | locomotory behavior | 5/37 | 0.008884801 |
| Saline | microtubule organizing center localization | 3/37 | 0.008884801 |
| Saline | modulation of chemical synaptic transmission | 6/37 | 0.016483875 |
| Saline | neurotransmitter uptake | 3/37 | 0.015830776 |
| Saline | regulation of trans-synaptic signaling | 6/37 | 0.016483875 |
| ICH 3 | leukocyte activation involved in immune response | 21/193 | 3.85E-09 |
| ICH 3 | leukocyte cell-cell adhesion | 23/193 | 1.77E-08 |
| ICH 3 | myeloid cell differentiation | 21/193 | 2.50E-07 |
| ICH 3 | myeloid leukocyte activation | 20/193 | 1.48E-09 |
| ICH 3 | myeloid leukocyte differentiation | 14/193 | 3.85E-06 |
| ICH 3 | positive regulation of cell activation | 25/193 | 1.06E-08 |
| ICH 3 | positive regulation of cell adhesion | 25/193 | 1.73E-08 |
| ICH 3 | regulation of myeloid leukocyte differentiation | 10/193 | 2.25E-05 |
| ICH 3 | regulation of myeloid leukocyte mediated immunity | 8/193 | 9.65E-06 |
| ICH 3 | regulation of nervous system process | 6/193 | 0.029502024 |
| ICH 3 | synaptic transmission, glutamatergic | 6/193 | 0.007178844 |
| ICH 7 | leukocyte activation involved in immune response | 20/188 | 1.42E-08 |
| ICH 7 | leukocyte cell-cell adhesion | 26/188 | 1.08E-10 |
| ICH 7 | myeloid cell differentiation | 17/188 | 4.74E-05 |
| ICH 7 | myeloid leukocyte activation | 18/188 | 1.68E-08 |
| ICH 7 | myeloid leukocyte differentiation | 10/188 | 0.001735174 |
| ICH 7 | positive regulation of cell activation | 23/188 | 8.01E-08 |
| ICH 7 | positive regulation of cell adhesion | 28/188 | 1.08E-10 |
| ICH 7 | regulation of myeloid leukocyte mediated immunity | 7/188 | 0.000123253 |
| ICH 14 | modulation of chemical synaptic transmission | 13/146 | 0.006760832 |
| ICH 14 | potassium ion transmembrane transport | 13/146 | 1.95E-05 |
| ICH 14 | potassium ion transport | 13/146 | 3.26E-05 |
| ICH 14 | regulation of nervous system process | 9/146 | 0.000771184 |
| ICH 14 | regulation of trans-synaptic signaling | 13/146 | 0.006760832 |
| ICH 14 | synaptic transmission, glutamatergic | 10/146 | 1.31E-05 |
| ICH+Exo 3 | locomotory behavior | 5/94 | 0.040984323 |
| ICH+Exo 3 | modulation of chemical synaptic transmission | 12/94 | 0.001426258 |
| ICH+Exo 3 | potassium ion transmembrane transport | 8/94 | 0.001919653 |
| ICH+Exo 3 | potassium ion transport | 9/94 | 0.001532448 |
| ICH+Exo 3 | regulation of nervous system process | 7/94 | 0.001910936 |
| ICH+Exo 3 | regulation of trans-synaptic signaling | 12/94 | 0.001426258 |
| ICH+Exo 3 | synaptic transmission, glutamatergic | 6/94 | 0.001910936 |
| ICH+Exo 3 | synaptic vesicle cycle | 6/94 | 0.012110431 |
| ICH+Exo 7 | locomotory behavior | 4/17 | 0.004603394 |
| ICH+Exo 7 | regulation of nervous system process | 4/17 | 0.004125433 |
| ICH+Exo 14 | locomotory behavior | 12/145 | 1.27E-05 |
| ICH+Exo 14 | modulation of chemical synaptic transmission | 20/145 | 1.58E-07 |
| ICH+Exo 14 | potassium ion transmembrane transport | 7/145 | 0.033080402 |
| ICH+Exo 14 | potassium ion transport | 7/145 | 0.047793541 |
| ICH+Exo 14 | regulation of nervous system process | 6/145 | 0.021937043 |
| ICH+Exo 14 | regulation of trans-synaptic signaling | 20/145 | 1.58E-07 |
| ICH+Exo 14 | synaptic transmission, glutamatergic | 6/145 | 0.005543411 |

**Table S5**. Canonical marker genes used to identify subtypes

| Subtype | Gene |
| --- | --- |
| Ast | Itih3, Etnppl, Aox1, Fmo2, RGD1560146, Gfap, Ccdc103, Grin2c, Ednrb, Gramd2b |
| MDM | Slco1a2, Tmem72, Ttr, Lmx1a, Igf2, Cgnl1, F5, Cdh3, Cfap65, Scara5 |
| Chst9 GABA | Chst9, Col14a1, Eya2, Mdfic, Foxp2, Fgf10, Otof, Gng4, Meis2, Erbb4 |
| DC | Ms4a4c, Gzmk, Serpinb8, Ptpn22, Treml4, Prrx2, Anxa2, Mt-nd3, Mt-nd5, Themis |
| DG Glut | Ntf3, Glis3, Palmd, Ptpn14, Syt17, Vwa3a, Ccdc27, Il16, Pla2g4f, Kcnj6 |
| Endo | Slc6a13, Abcc9, Tbx15, Prrx2, Akr1d1, Foxd1, Atp13a5, Rgs5, Bmp5, C7 |
| Epen | Vwf, Spata18, Kcnk16, Erg, Ankdd1b, Flacc1, Ankrd66, Egfl7, Zfp366, Adgrl4 |
| Folh1 GABA | Ankfn1, Cmtm8, Col11a1, Col9a1, Drd3, Edar, Epha3, Esr1, Fstl1, Gpr149 |
| L2/3-IT-CTX-Glut | Slco2a1, Ly6k, Cyp11b3, Robo3, Nipal1, RGD1565367, LOC689599, Plk5, Arhgap10, Bin2a |
| L4/5-IT-CTX-Glut | Boc, Catsperz, Cd200r1, Cdh12, Clca4, Clca4l, Cnih3, Col13a1, Cyp11b3, Cyp26b1 |
| L5-ET-CTX-Glut | Nox3, Eaf2, Cftr, Npr3, Inhbc, Naglt1, Chst8, Sema3c, Gm9918, Klhl1 |
| L5-IT-CTX-Glut | Bmper, Catsperz, Chst8, Col6a2, Cyp11b3, Esrp1, Esrrg, Gxylt2, Hes1, Il1rapl2 |
| L5-NP-CTX-Glut | Trabd2b, Adamts18, Vwc2l, Lama4, Ddx43, Chrdl1, Tmem100, Htr4, Chrna5, Unc13b |
| L6-CT-CTX-Glut | Antxr1, Ccn1, Ccn2, Chrna5, Clic5, Dmgdh, Dnah6, Etl4, Hcrtr2, Hs3st2 |
| L6-IT-CTX-Glut | Adamts2, Bmper, Clstn2, Col6a2, Cyp11b3, Galnt14, Hes1, Itprid1, Lipm, Ly6k |
| Lamp5 GABA | Adarb2, Adra1a, Alk, Cpne7, Cps1, Egfr, Fam83b, Fgf13, Frem1, Grik1 |
| LSX Nkx2-GABA | Ano1, Ano2, Atp8b1, Clic4, Col25a1, Crhr2, Dgkg, Dnah11, Dnah9, Fgd5 |
| Microglia | Bco2, Fyb1, Blnk, Dock8, Stab1, Pik3cg, Pik3ap1, Fcrl1, Slco2b1, Fermt3 |
| Neutrophil | Bub1, Bub1b, Cdca2, Cenpe, Cenpf, Cenph, Cep55, Ckap2, Depdc1, Diaph3 |
| Oligo | Abca8a, Aspa, Cdh19, Cyp2j10, Ermn, Fa2h, Galnt6, Gpd1, Hapln2, Hhip |
| OPC | Afap1l2, Arhgap31, Armh4, Bcas1, Bmp4, C1ql1, Calcrl, Cdh3, Cfap52, Col20a1 |
| HPF Fzd5 Glut | Baiap3, Chat, Chodl, Dgkk, Dlk1, Dysf, Ebf3, Ecel1, Glra3, Gpr139 |
| Pvalb GABA | Inhbc, Mybpc1, Adamts15, Kcnc2, Kcnmb2, Vwc2, Btbd11, Cps1, Rnf144b, Kcnip1 |
| Sema5 GABA | Rlbp1, Col5a3, Arhgap31, Afap1l2, Fam89a, Pdgfra, Shc4, Casr, Bcas1, Nipal1 |
| Sst GABA | Npas1, Lypd6b, Reln, Cdk15, Grin3a, Crhbp, Cdh9, Col19a1, Slc45a2, Elfn1 |
| STR-D1 GABA | Meox2, Rgs9, Sh3rf2, Syndig1l, Itgb6, Dach1, Strip2, Il20ra, Mme, Rarb |
| STR-D2 GABA | Cyp2c23, Rgs9, Meox2, Dach1, Sh3rf2, Itgb6, Gnal, Adamts19, Rarb, Foxp2 |
| Th Prkcd Grin2c-Glut | Ndst4, Ntng1, Qrfpr, Lepr, Bin2a, Tfap2d, Ptpn14, Abi3bp, Grid2ip, Mybpc1 |
| Vip GABA | Cps1, Adarb2, Inhbc, Frem1, Yjefn3, Pde5a, Egfr, Slc44a5, Th, Rgs12 |

**Table S6**. Table of GSEA GO biological process enrichment results of specific genes in different groups of scRNA-seq data.

| Subclass | Description | GeneRatio | p.adjust |
| --- | --- | --- | --- |
| Mptn | synapse assembly | 12/371 | 0.00654 |
| Mptn | regulation of synaptic plasticity | 12/371 | 0.00632 |
| Mptn | axonogenesis | 24/371 | 0.000326 |
| Mptn | regulation of neurogenesis | 27/371 | 2.32E-06 |
| Mptn | regulation of nervous system development | 30/371 | 2.22E-06 |
| Mptn | glial cell differentiation | 24/371 | 9.21E-08 |
| Mptn | regulation of nervous system process | 24/371 | 5.32E-08 |
| MIkzf1 | regulation of immune effector process | 22/264 | 5.08E-06 |
| MIkzf1 | immune response-activating signaling pathway | 25/264 | 2.62E-06 |
| MIkzf1 | cell activation involved in immune response | 20/264 | 2.6E-06 |
| MIkzf1 | leukocyte activation involved in immune response | 20/264 | 2.34E-06 |
| MIkzf1 | positive regulation of leukocyte activation | 29/264 | 2.15E-10 |
| MResident | glial cell differentiation | 25/858 | 0.00303 |
| MResident | regulation of neurogenesis | 43/858 | 4.46E-06 |
| MResident | regulation of nervous system development | 54/858 | 3.17E-08 |
| MResident | regulation of nervous system process | 25/858 | 1.66E-08 |
| MResident | regulation of synapse structure or activity | 40/858 | 6E-10 |
| MResident | regulation of synaptic plasticity | 37/858 | 3.92E-10 |
| MResident | regulation of synapse organization | 40/858 | 3.06E-10 |
| Mfew | regulation of immune effector process | 16/241 | 0.00234 |
| Mfew | immune response-activating signaling pathway | 19/241 | 0.000834 |
| Mfew | cell activation involved in immune response | 16/241 | 0.000254 |
| Mfew | leukocyte activation involved in immune response | 16/241 | 0.00024 |
| Mfew | positive regulation of leukocyte activation | 20/241 | 0.000049 |

**Table S7**. Target gene of Ikzf1.

| highlightedTFs | NES | AUC | TF_highConf |
| --- | --- | --- | --- |
| Ikzf1 | 5.98 | 0.0419 | Ikzf1 (inferredBy_Orthology). |
| Ikzf1 | 11.2 | 0.0904 | Spib (inferredBy_Orthology). |
| Ikzf1 | 10.6 | 0.0775 | Spib (inferredBy_Orthology). |
| Ikzf1 | 10.5 | 0.0602 | Spib (inferredBy_Orthology). |
| Ikzf1 | 9.52 | 0.0563 | Spi1 (directAnnotation). |
| Ikzf1 | 9.35 | 0.0556 | Spib (inferredBy_Orthology). |
| Ikzf1 | 9.27 | 0.0552 | Spi1 (directAnnotation). |
| Ikzf1 | 9.09 | 0.0767 | Spi1 (directAnnotation). |
| Ikzf1 | 8.86 | 0.0536 | Spi1 (directAnnotation). |
| Ikzf1 | 8.53 | 0.0662 | Spi1 (inferredBy_Orthology). |
| Ikzf1 | 8.51 | 0.0522 | Spi1 (inferredBy_Orthology). |
| Ikzf1 | 8.47 | 0.052 | Spi1 (inferredBy_Orthology). |
| Ikzf1 | 8.3 | 0.0513 | Spi1 (inferredBy_Orthology). |
| Ikzf1 | 8.25 | 0.0511 | Spi1 (inferredBy_Orthology). |
| Ikzf1 | 8.17 | 0.0642 | Spi1 (inferredBy_Orthology). |
| Ikzf1 | 7.48 | 0.048 | Spi1 (inferredBy_Orthology). |
| Ikzf1 | 7.37 | 0.0657 | Irf1 (directAnnotation). |
| Ikzf1 | 7.28 | 0.0472 | Spib (inferredBy_Orthology). |
| Ikzf1 | 7.24 | 0.0648 | Irf2 (inferredBy_Orthology). |
| Ikzf1 | 6.93 | 0.0575 | Irf1 (inferredBy_Orthology). |
| Ikzf1 | 6.92 | 0.0574 | Ep300 (inferredBy_Orthology). |
| Ikzf1 | 6.9 | 0.0456 | Spib (directAnnotation). |
| Ikzf1 | 6.85 | 0.0454 | Irf4; Spi1 (inferredBy_Orthology). |
| Ikzf1 | 6.58 | 0.0443 | Irf4 (inferredBy_Orthology). |
| Ikzf1 | 6.49 | 0.044 | Spi1 (inferredBy_Orthology). |
| Ikzf1 | 6.45 | 0.0438 | Spi1 (inferredBy_Orthology). |
| Ikzf1 | 6.43 | 0.0437 | Rela (inferredBy_Orthology). |
| Ikzf1 | 6.36 | 0.0591 | Spi1 (directAnnotation). |
| Ikzf1 | 6.32 | 0.0589 | Irf1 (inferredBy_Orthology). |
| Ikzf1 | 6.25 | 0.0537 | Irf1 (inferredBy_Orthology). |
| Ikzf1 | 6.13 | 0.0531 | Irf1 (inferredBy_Orthology). |
| Ikzf1 | 6.08 | 0.0573 | Spi1 (directAnnotation). |
| Ikzf1 | 5.72 | 0.0508 | Irf8 (inferredBy_Orthology). |
| Ikzf1 | 5.68 | 0.0506 | Irf1 (inferredBy_Orthology). |
| Ikzf1 | 5.66 | 0.0505 | Stat1 (inferredBy_Orthology). |
| Ikzf1 | 5.61 | 0.0404 | Elf1 (inferredBy_Orthology). |
| Ikzf1 | 5.31 | 0.0486 | Irf4 (directAnnotation). |
| Ikzf1 | 5.31 | 0.0392 | Bcl11a (inferredBy_Orthology). |
| Ikzf1 | 5.15 | 0.0385 | Elf1 (inferredBy_Orthology). |
| Ikzf1 | 4.91 | 0.0464 | Stat3 (inferredBy_Orthology). |
| Ikzf1 | 4.53 | 0.0443 | Spi1 (inferredBy_Orthology). |
| Ikzf1 | 4.52 | 0.0472 | Spi1 (directAnnotation). |
| Ikzf1 | 4.19 | 0.0346 | Tbl1xr1 (inferredBy_Orthology). |
| Ikzf1 | 3.32 | 0.0311 | Irf8 (inferredBy_Orthology). |
| Ikzf1 | 3.26 | 0.0374 | Spi1 (directAnnotation). |
| Ikzf1 | 3.17 | 0.0368 | Mta3 (inferredBy_Orthology). |
| Ikzf1 | 3.16 | 0.0545 | Runx2 (inferredBy_Orthology). |

**Table S8**. Table of GSEA GO biological process enrichment results of target gene of Ikzf1.

| Description | p.adjust | geneID |
| --- | --- | --- |
| mononuclear cell differentiation | 2.11E-05 | DOCK11/SH3RF1/PREX1/ZMIZ1/BLNK/IKZF1/PTPRJ/LYN/INPP5D/CSF1R/RUNX1 |
| lymphocyte differentiation | 3.89E-04 | DOCK11/SH3RF1/PREX1/ZMIZ1/BLNK/IKZF1/PTPRJ/INPP5D/RUNX1 |
| myeloid leukocyte differentiation | 7.11E-03 | LRRK1/GAB2/LYN/INPP5D/CSF1R/RUNX1 |
| B cell activation | 1.46E-02 | DOCK11/BLNK/SKAP2/PTPRJ/LYN/INPP5D |
| positive regulation of cell adhesion | 1.18E-02 | PREX1/ZMIZ1/DISC1/PTPRJ/LYN/RUNX1/ZFHX3 |
| Fc-gamma receptor signaling pathway involved in phagocytosis | 7.16E-03 | PTPRJ/LYN/HCK |
| immune response-regulating cell surface receptor signaling pathway | 1.39E-02 | BLNK/PTPRJ/LYN/LCP2/INPP5D/HCK |
| Fc-gamma receptor signaling pathway | 7.27E-03 | PTPRJ/LYN/HCK |
| Fc receptor mediated stimulatory signaling pathway | 9.07E-03 | PTPRJ/LYN/HCK |
| immune response-activating signal transduction | 1.08E-02 | BLNK/PTPRJ/LYN/LCP2/INPP5D/HCK |
| immune response-activating cell surface receptor signaling pathway | 1.08E-02 | BLNK/PTPRJ/LYN/LCP2/INPP5D/HCK |
| immune response-regulating cell surface receptor signaling pathway | 1.39E-02 | BLNK/PTPRJ/LYN/LCP2/INPP5D/HCK |
| immune response-regulating signaling pathway | 7.16E-03 | PIK3AP1/BLNK/PTPRJ/LYN/LCP2/INPP5D/HCK/CTSS |

**Table S9**. Table of GSEA GO biological process enrichment results of genes (group I-III) that exhibited changes from the initial state to two distinct cell fates.

| Description | geneID |
| --- | --- |
| lymphocyte differentiation | Pou2f2/Cd4/Ptprj/Mertk/Prex1/Nckap1l/Il18/Atm/Tgfbr2/Inpp5d/Ikzf1/Ptprc/Fcer1g/Pik3r6/Runx1/Irf8/Hdac9/Syk/Dock11/Dclre1c/B2m/Plcl2/Ptpn6/Nlrp3/Zfp36l1/Sh3rf1/Ap3b1/Tnfsf13b/Cd74/Igtp/Cmtm7/Rbpj/Itpkb/Mafb/Relb/Atp7a/Plcg2/Spi1/Cbfb/Zfp36l2/Vav1/Axl/Nfam1/Ctsl/Malt1/Il15ra/Pnp/Btk/Tcirg1/Tnfaip3/Cd83/Rhoh/Irf1/Nfatc1/Tgfb1/Nfkbiz/Smarca2/Arid2/Stat6/Il1b/Lipa/Rc3h1/Vsir/Gpr183/St3gal1/Laptm5/Ep300/Sh2b3/Mfng/Adam17/Ripk3/Lgals9/Lyl1/Lig4/Sash3/Il7r/Fut7/Hhex/Cd79b/Jak3/Cebpg/Gpr18/Prdm1/Hlx/Slamf6/Il1a/Bcl3/Stat5a/Batf/Il18r1/Myb/Rhoa/Slamf1/Rsad2/Hectd1/Tnfsf4/Nhej1/Ifnar2 |
| positive regulation of cell activation | Gab2/Itgam/Dock8/Mef2c/Cd4/Ptprj/Tnfrsf14/Nckap1l/Il18/Tgfbr2/Inpp5d/Ptprc/Pla2g4a/Fcer1g/Pik3r6/Runx1/Csf1r/Itgal/Plek/Syk/Peli1/B2m/Nlrp3/Fgfr1/Ap3b1/Ptafr/Pdcd1lg2/Tnfsf13b/Tyrobp/Cd74/Havcr2/Ctsc/Itpkb/Sh3kbp1/Aif1/Spi1/Cbfb/Axl/Malt1/Rps6ka1/Il15ra/Pnp/Btk/Ticam2/Cd83/Rhoh/Irf1/Tgfb1/Nfkbiz/Smarca2/Arid2/Stat6/Itgb2/Tlr4/Cd274/Il1b/Vsir/Gpr183/Vav3/Cd86/Il1rl1/Trem2/Igf1/Fgr/Cd47/Tnip2/Il16/Shld2/Lgals9/Rasal3/Ccdc88b/Sash3/Cd38/Sirpa/Il7r/Cd6/Pdgfb/Jak3/Hlx/Il1a/Clec7a/Capn3/Tlr6/Stat5a/Il6st/Myb/Timd2/Myd88/Rhoa/Tnfsf13/Lilra5/Slamf1/Cd40/Coro1a/Tnfsf4/Ccl2 |
| regulation of immune effector process | Cd37/Gab2/Itgam/Ptprj/Lyn/Tnfrsf14/Tlr7/Nckap1l/Il18/C3/Ptprc/Cfh/Fcer1g/Cd84/Pik3r6/Ncf1/P2rx7/Syk/Cx3cr1/B2m/Ptpn6/Nlrp3/Ptafr/Irak3/Tyrobp/Cd74/Havcr2/Casp4/Plcg2/Spi1/Vav1/Tnfrsf1b/Stx7/Fer/Axl/Irf5/Cd300a/Malt1/Tlr3/Fes/Pnp/Btk/Ticam2/Tnfaip3/Rac2/Irf1/Tgfb1/Arrb2/Nfkbiz/Fcgr2b/Stat6/Itgb2/Tlr4/Tap1/Tlr2/Il1b/Rc3h1/Vsir/Cd86/Nod1/Trem2/Fgr/Laptm5/Tek/Cd22/Cd47/Grn/Lacc1/Hmox1/Shld2/Ripk3/Cfp/Lgals9/Traf2/Dhx58/Was/Casp1/Sash3/Cr1l/Il7r/Fut7/Jak3/Unc13d/Hlx/Slamf6/Clec7a/Rabgef1/Stat5a/Il18r1/Myd88/Tnfsf13/Slamf1/Cd40/Rsad2/Tnfsf4/Ccl2 |
| myeloid cell differentiation | Lrrk1/Gab2/Itgam/Mef2c/Cd4/Clec2g/Lyn/Ptbp3/Wasf2/Nckap1l/Fli1/Tgfbr2/Inpp5d/Ikzf1/Fcer1g/Runx1/Csf1r/Irf8/C1qc/B2m/Ptpn6/Ubash3b/Zfp36l1/Ap3b1/Tyrobp/Ets1/Cd74/Nrros/Stat1/Rbpj/Itpkb/Nedd9/Cflar/Mafb/Relb/Spi1/Abi1/Cbfb/Ucp2/Rb1/Tal1/Casp8/Fes/Tnfrsf11a/Tcirg1/Nfatc1/Tgfb1/Csf3r/Nfkbia/Dab2/Tlr4/Hcls1/Psen1/Tlr2/Lipa/Gpr183/Lmo2/Il1rl1/Rassf2/Trem2/Tifab/Ep300/Cd300lf/Ccl3/Cd101/Plscr1/Sh2b3/Cib1/Zfp385a/Ldb1/Maf/Sbno2/Inpp4b/Hhex/Cebpg/Rab7b/Evi2b/Cebpb/Hif1a/Pml/Stat5a/Itgb3/Batf/Myb/Myd88/Zfp36/Tmem64/Tfe3/Zfpm1/Naglu/Slc11a2/Slc1a5 |
| macrophage activation involved in immune response | Syk/Tyrobp/Havcr2/Plcg2/Trem2/Ifi35/Grn/Nmi/Sbno2 |
| inflammatory cell apoptotic process | Itgam/Mef2c/Il18/Itpkb/Pik3cd/Pik3cb/Ccr5/Fcgr2b/Plekho2 |
| positive regulation of inflammasome-mediated signaling pathway | Casp4/Plcg2/Btk/Nek7/Tlr4/Gbp5/Gbp2/Tlr6/Myd88 |
| regulation of hemopoiesis | Itgam/Mef2c/Tmem176b/Cd4/Clec2g/Lyn/Nckap1l/Il18/Tgfbr2/Inpp5d/Ikzf1/Ptprc/Pik3r6/Runx1/Csf1r/Syk/C1qc/B2m/Ptpn6/Nlrp3/Ubash3b/Zfp36l1/Sh3rf1/Ap3b1/Tyrobp/Ets1/Cd74/Stat1/Itpkb/Nedd9/Mafb/Spi1/Cbfb/Zfp36l2/Rb1/Tal1/Axl/Nfam1/Casp8/Malt1/Fes/Il15ra/Pnp/Tmem176a/Cd83/Rhoh/Irf1/Tgfb1/Csf3r/Nfkbia/Nfkbiz/Smarca2/Arid2/Hcls1/Rc3h1/Vsir/Lmo2/Rassf2/Trem2/Tsc22d1/Ccl3/Cd101/Sh2b3/Cib1/Ldb1/Lgals9/Inpp4b/Sash3/Il7r/Jak3/Rab7b/Prdm1/Evi2b/Hlx/Cebpb/Hif1a/Stat5a/Itgb3/Myb/Rhoa/Zfp36/Tmem64/Tfe3/Zfpm1/Tnfsf4 |
| B cell activation | Pou2f2/Swap70/Blnk/Mef2c/Fcrl1/Skap2/Ptprj/Lyn/Nckap1l/Atm/Inpp5d/Ikzf1/Ptprc/Irf8/Hdac9/Syk/Dock11/Peli1/Dclre1c/Plcl2/Ptpn6/Zfp36l1/Tnfsf13b/Tyrobp/Cd74/Cmtm7/Rbpj/Sh3kbp1/Prkcb/Pik3cd/Plcg2/Spi1/Zfp36l2/Nfam1/Cd300a/Malt1/Pnp/Btk/Tcirg1/Tnfaip3/Prkcd/Nfatc1/Tgfb1/Nfkbiz/Fcgr2b/Stat6/Tlr4/Rc3h1/Gpr183/St3gal1/Vav3/Cd86/Laptm5/Cd22/Cd180/Ep300/Lat2/Tnip2/Mfng/Adam17/Shld2/Themis2/Lyl1/Lig4/Sash3/Cd38/Il7r/Hhex/Cd79b/Jak3/Cebpg/Bcl3/Stat5a/Batf/Myb/Cxcr5/Tnfsf13/Cd40/Tnfsf4/Nhej1 |
| cognition | Atp1a3/Nrxn2/Syt11/Tac1/Ptn/Scn2a/Map1a/Prnp/Snap25/Tuba1a/Ncam1/Cck/App/Ndrg4/Tusc3/Camk2n1/Slc17a7/Vip/Gpr88/Pgrmc1/Slc1a1/Ppp1r1b/Clstn3/Slc8a2/Shisa7/Chl1/Cnr1/Syt4/Thra/Slc6a1/Nptn/B3gat1/Pja2/Vdac1/Mapk8ip2/Nlgn3/Synpo/Asic1/Jakmip1/Plk2/Calb1/Ptchd1/Hmgcr/Arl6ip5/Clstn2/Gabrb3/Hrh3/Chrna7/Vdac3/Jph3/Gabra5/Adcy3/Htr2a/Pak6/Egr1/Crh/Prrt1/Fgf13/Gpr155/Nts/Itga3/B4galt2/Lamb1/Nptx2/Drd1/Syngap1/Bdnf/Btg2/Neurod2/Htr6/Kit/Jun/Paip2/Adra1b/Tbr1/Gnas/Ntf3/Mme/Abi2/Aph1b/Grm4/Chat/Psen2/Arc/Rin1/Ngf/Ppt1/Jph4/Bhlhb9/Ptgs2/Dgcr2/Gm2a/Chrna4/Lhx8/Oprk1 |
| learning or memory | Atp1a3/Nrxn2/Syt11/Tac1/Ptn/Scn2a/Map1a/Prnp/Snap25/Tuba1a/Ncam1/Cck/App/Ndrg4/Camk2n1/Slc17a7/Vip/Gpr88/Pgrmc1/Slc1a1/Ppp1r1b/Clstn3/Slc8a2/Shisa7/Cnr1/Syt4/Thra/Slc6a1/Nptn/B3gat1/Pja2/Vdac1/Mapk8ip2/Nlgn3/Synpo/Asic1/Plk2/Calb1/Ptchd1/Hmgcr/Arl6ip5/Clstn2/Gabrb3/Hrh3/Chrna7/Vdac3/Jph3/Gabra5/Adcy3/Htr2a/Pak6/Egr1/Crh/Prrt1/Fgf13/Nts/Itga3/B4galt2/Lamb1/Nptx2/Drd1/Syngap1/Bdnf/Btg2/Neurod2/Htr6/Kit/Jun/Paip2/Adra1b/Tbr1/Ntf3/Mme/Abi2/Aph1b/Grm4/Chat/Psen2/Arc/Rin1/Ngf/Ppt1/Jph4/Bhlhb9/Ptgs2/Gm2a/Lhx8/Oprk1 |
| cell junction assembly | Nrxn2/Gpm6a/App/Apod/Clstn1/Cd9/Arhgef9/Cldn11/Gap43/Lrrc4b/Nptxr/Bsn/Gabrg2/Adgrl2/Snca/Clstn3/Oxt/Sptbn2/Actb/Gabra1/Thy1/Adgrb2/Jam3/Adgrl1/Nptn/Nptx1/Dmtn/Slitrk1/Cfl1/Flrt3/Pmp22/Nlgn3/Akap5/Add2/Cadm1/Nectin1/Slitrk5/Efnb3/Icam5/Clstn2/Gabrb3/Slitrk3/Cdh7/Cntnap1/Lrrtm2/Nlgn2/Chd4/Psd/Lrtm2/Dlg4/Lgi2/Fkrp/Lrrn1/Slitrk2/Flrt1/Lhfpl4/C1ql3/Hapln4/Fgf13/Ache/Lrfn4/Lrrc24/Bdnf/Lrrn3/Cbln2/Eif4g1/Efnb2/Lrrc4/Amigo1/Wnt7a/St8sia2/Gpc4/Ptk2b/Actg1/Prkaca/Abi2/Cldn5/Lrfn3/Cript/Ramp2/Hopx/Ube2v2/Bhlhb9/Zdhhc12/S100a10/C1ql2/Cdc42/Rap2a/Fn1/Plxnd1 |
| regulation of neurogenesis | Map1b/Ptn/Camk2b/Rtn4/Epha4/Cx3cl1/Mag/Cdkl5/Syt4/Thy1/Dbn1/Ttc3/Lingo1/Kifap3/Nefl/Fzd3/Nptn/Sema3e/Bmp4/Mt3/Slitrk1/Serpine2/Sema3a/Itpka/Akap5/Nap1l1/Efnb3/Sema6b/Myo5b/Zfp365/Foxg1/Dag1/Rtn4r/Dlx1/Gjc2/Prpf19/Sema4f/Dlg4/Gpr37l1/Dnajb11/Olig2/Hapln4/Vim/Fgf13/Lhx2/Hes1/Ache/Rpl4/Nkx6-2/Syngap1/Bdnf/Btg2/Hapln1/Amigo1/Wnt7a/Kit/Sema7a/Ntf3/Mme/Ufl1/Vegfc/Crabp2/Nr1d1/Cxcl12/Hdac2/Wnt2/Stk25/Fbxo31/Ngf/Sema3f/Map2k1/Trim32/Adcyap1/Bhlhb9/S100a10/Mycn/Fezf2/Fxn/Met/Acan/Id1/Fn1/Rheb/Plxnd1/Plxna1 |
| regulation of synapse structure or activity | Ptn/Prnp/Tuba1a/Hspa8/Sparcl1/Camk2b/Gpm6a/App/Clstn1/Epha4/Slc17a7/Myh10/Kif1a/Lrrc4b/Pgrmc1/Cdkl5/Adgrl2/Snca/Clstn3/Oxt/Ngef/Tubb5/Dbn1/Adgrb2/Nefl/Adgrl1/Slitrk1/Cfl1/Flrt3/Itpka/Nlgn3/Nectin1/Slitrk5/Efnb3/Icam5/Myo5b/Dag1/Clstn2/Dctn1/Slitrk3/Chrna7/Ywhaz/Cntnap1/Lrrtm2/Nlgn2/Chd4/Psd/Ctnna2/Lrtm2/Dlg4/Lrrn1/Slitrk2/Flrt1/Lhfpl4/C1ql3/Pcdh8/Lrfn4/Rims4/Cdk5r1/Syngap1/Lrrc24/Bdnf/Lrrn3/Cbln2/Sparc/Eif4g1/Neurod2/Amigo1/Wnt7a/Camk1/St8sia2/Gpc4/Ptk2b/Zfp804a/Lrfn2/Rhob/Abi2/Lrfn3/Cript/Psen2/Arc/Sema3f/Vcp/Ube2v2/Bhlhb9/Pick1/Arhgap33/Slc17a6/Efna1/Srgn/Abhd17a/C1ql2/Cdc42/Rap2a/Rheb |
| regulation of synapse organization | Ptn/Prnp/Tuba1a/Hspa8/Sparcl1/Camk2b/Gpm6a/App/Clstn1/Epha4/Myh10/Kif1a/Lrrc4b/Pgrmc1/Cdkl5/Adgrl2/Snca/Clstn3/Oxt/Ngef/Tubb5/Dbn1/Adgrb2/Nefl/Adgrl1/Slitrk1/Cfl1/Flrt3/Itpka/Nlgn3/Nectin1/Slitrk5/Efnb3/Icam5/Myo5b/Dag1/Clstn2/Dctn1/Slitrk3/Chrna7/Ywhaz/Cntnap1/Lrrtm2/Nlgn2/Chd4/Psd/Ctnna2/Lrtm2/Dlg4/Lrrn1/Slitrk2/Flrt1/Lhfpl4/C1ql3/Pcdh8/Lrfn4/Rims4/Cdk5r1/Lrrc24/Bdnf/Lrrn3/Cbln2/Sparc/Eif4g1/Neurod2/Amigo1/Wnt7a/Camk1/St8sia2/Gpc4/Ptk2b/Zfp804a/Lrfn2/Rhob/Abi2/Lrfn3/Cript/Psen2/Arc/Sema3f/Vcp/Ube2v2/Bhlhb9/Arhgap33/Efna1/Srgn/Abhd17a/C1ql2/Cdc42/Rap2a/Rheb |
| regulation of membrane potential | Gabrg3/Ntrk3/Nlgn1/Pex5l/Ank2/Dpp6/Cacna2d1/Pclo/Dgki/Grin2b/Grin1/Scn1a/Dmd/Nrxn1/Nrcam/Akap6/Rims2/Kcnq3/Rbfox2/Scn8a/Nedd4/Rims1/Slc4a4/Gabrb1/Gria1/Mapt/Kcnma1/Ntrk2/Ryr2/Cacna1d/Igsf11/Fgf12/Neto1/Cacna1a/Ank3/Grik2/Fgf14/Hcn1/Gabra2/Ppp1r9a/Rgs7/Gabrb2/Gabra4/Kcnc2/Grid2/Rapgef4/Nalcn/Cxadr/Kcnk2/Grik5/Cacnb4/Asic2/Reln/Grin2a/Stx1b/Slc4a3/Atxn1/Gja1/Rimbp2/Nedd4l/Slc25a27/Kcnd3/Kcnh5/Cacna1c/Kcnip1/Prkcz/Unc13b/Baiap2/Shank3/Slc8a3/Agt/Dmpk/Scn9a/Slc4a8/Grik1/Gabrg1/Abat/Cacna1g/Shank1/Prkar1b/Begain/Slc24a4/Kcnq1/Afdn/Ptpn3/Grik3/Kcnq2/Actn2/Dlg1/Nos1/Mpp2/Fhl1/Lrrk2/Zmynd8/Trpc5/Mfn1/Adra1a/Grin3a/Agrn/Snta1/Grip2/Nr3c2/Grin2c/Fxyd1/Foxp1/Cacna1h/Cnih3/Scn5a/Cux2/Igsf9b/Prkce/Kcnn2/Kcnj9/Usp53/Kcnab2/Oprd1/Rnf207/Celf4/Tacr1/Abcb5/Kcnk4/Rims3/Cacnb2/Npr2/Bok/Piezo2/Cdk5/Ehd3/Gna14/Slc25a33/Insyn2a/Ppp3ca/Abcc9/Crtc1/Npas4/Kcnh8/Dvl1/Scn7a/Myh14/Bin1/Flna/Pawr/Chrnb2/Clcn1/Kcnk10/Atp1a4/Kcnh6/Kcnip2 |
| cell junction assembly | Ntrk3/Cdh10/Ctnnd2/Nlgn1/Negr1/Magi2/Pclo/Lrrtm4/Grin1/Ptprd/Gpm6b/Il1rapl1/Il1rapl2/Nrxn1/Lrfn5/Mdga2/Nrxn3/Cntn5/Adgrb3/Adgrl3/Limch1/Nfasc/Dnm3/Gria1/Mapt/Ntrk2/Robo1/Cdh8/Cacna1a/Cdh13/Lrrtm3/Erbb4/Ptprk/Gabra2/Ppp1r9a/Pard3/Gabrb2/Cdh2/Large1/Cdh11/Grid2/Sorbs1/Farp1/Actn1/Epha7/Asic2/Epha3/Reln/Cdh18/Pcdh17/Cdh9/Cdh12/Gja1/Nedd4l/Mpdz/Syndig1/Flrt2/Ptprs/Elavl2/Ephb1/Numbl/Shank3/Efna5/Nrg2/Bcr/Lzts3/Epb41l3/Agt/Ptk2/Plec/Srcin1/Zdhhc2/Mpp7/Coro2b/Sdk2/Nrg1/Kirrel3/Vegfa/Fermt2/Afdn/Tjp1/Actn2/Dlg1/Shank2/Vcl/Macf1/Cdh20/Prkca/Agrn/Rock1/Sipa1l1/Cdh6/Col16a1/Ephb2/Lamc1/Dlg5/Cldn10/Cux2/Mdga1/Dlc1/Abi3bp/Lrp1/Cdh4/Actn4/Dock7/Cdh19/Ptpn13/Enpp2/Fam107a/Sdc4/Slit2/Dusp3/Clasp2/Ephb3/Pip5k1a/Phldb2/Fbf1/Epb41l5/Patj/Arvcf/Mycbp2/Nphp4/Cdk5/Nr1h4/Adnp/Cdh22/Fmn1/Vstm5/Ptpn11/Npas4/Dvl1/Camsap3/Ppm1f/Gjc1/Il1rap/Ptpro/Chrnb2/Iqgap1/Itgb4/Arhgap6 |
| regulation of synapse organization | Ntrk3/Nlgn1/Negr1/Magi2/Cttnbp2/Lrrtm4/Grin2b/Grin1/Ptprt/Ptprd/Il1rapl1/Il1rapl2/Nrxn1/Dgkb/Nrcam/Lrfn5/Mdga2/Nedd4/Adgrb3/Adgrl3/Dnm3/Ntrk2/Gpr158/Robo1/Kalrn/Cdh8/Lrrtm3/Ppfia2/Ppp1r9a/Cdh2/Grid2/Rapgef4/Farp1/Arhgap44/Epha7/Camkv/Asic2/Ube3a/Snap91/Reln/Fyn/Grid1/Homer1/Pak3/Syndig1/Flrt2/Ptprs/Elavl2/Ephb1/Numbl/Baiap2/Shank3/Efna5/Nrg2/Lzts3/Dmpk/Ptk2/Srcin1/Frmpd4/Nrg1/Nf1/Ptprf/Afdn/Ncan/Drd2/Tanc2/C1ql1/Cntnap4/Tanc1/Mark2/Shank2/Lrrk2/Septin11/Zmynd8/Mfn1/Dab2ip/Prkca/Agrn/Rock1/Sipa1l1/Ephb2/Slc7a11/Dlg5/Itsn1/Lrp8/Cux2/Mdga1/Tiam1/Abi3bp/Ptpn13/Slit2/Ephb3/Rims3/Gripap1/Mycbp2/Cdk5/Zdhhc15/Adnp/Cask/Vstm5/Dvl1/Ror2/Il1rap/Ptpro/Chrnb2/Dhx36/Tsc2 |
| regulation of synapse structure or activity | Ntrk3/Nlgn1/Negr1/Magi2/Cttnbp2/Lrrtm4/Grin2b/Grin1/Ptprt/Ptprd/Il1rapl1/Il1rapl2/Nrxn1/Dgkb/Nrcam/Lrfn5/Mdga2/Nedd4/Adgrb3/Adgrl3/Dnm3/Ntrk2/Gpr158/Robo1/Kalrn/Cdh8/Lrrtm3/Ppfia2/Ppp1r9a/Cdh2/Grid2/Sybu/Rapgef4/Farp1/Arhgap44/Epha7/Camkv/Asic2/Ube3a/Snap91/Reln/Fyn/Grid1/Homer1/Pak3/Syndig1/Flrt2/Ptprs/Elavl2/Ephb1/Numbl/Baiap2/Shank3/Efna5/Nrg2/Lzts3/Dmpk/Ptk2/Srcin1/Frmpd4/Nrg1/Nf1/Ptprf/Afdn/Ncan/Drd2/Tanc2/C1ql1/Cntnap4/Tanc1/Mark2/Shank2/Lrrk2/Septin11/Zmynd8/Mfn1/Dab2ip/Prkca/Agrn/Rock1/Sipa1l1/Ephb2/Slc7a11/Dlg5/Itsn1/Lrp8/Cux2/Mdga1/Tiam1/Abi3bp/Ptpn13/Slit2/Ephb3/Rims3/Gripap1/Mycbp2/Cdk5/Zdhhc15/Adnp/Cask/Vstm5/Dvl1/Ror2/Il1rap/Ptpro/Chrnb2/Dhx36/Tsc2 |
| synapse assembly | Ntrk3/Nlgn1/Negr1/Magi2/Pclo/Lrrtm4/Grin1/Ptprd/Il1rapl1/Il1rapl2/Nrxn1/Lrfn5/Mdga2/Nrxn3/Cntn5/Adgrb3/Adgrl3/Dnm3/Gria1/Mapt/Ntrk2/Robo1/Cacna1a/Lrrtm3/Erbb4/Gabra2/Ppp1r9a/Gabrb2/Cdh2/Large1/Grid2/Farp1/Epha7/Asic2/Reln/Pcdh17/Cdh9/Syndig1/Flrt2/Ptprs/Elavl2/Ephb1/Numbl/Shank3/Efna5/Nrg2/Lzts3/Ptk2/Srcin1/Zdhhc2/Sdk2/Nrg1/Kirrel3/Shank2/Prkca/Agrn/Sipa1l1/Ephb2/Dlg5/Cux2/Mdga1/Abi3bp/Dock7/Ptpn13/Slit2/Ephb3/Mycbp2/Cdk5/Adnp/Vstm5/Npas4/Dvl1/Il1rap/Chrnb2 |
| locomotory behavior | Grm1/Grm5/Negr1/Grin1/Slc4a10/Scn1a/Dmd/Scn8a/Mapk10/Adgrl3/Cacna1e/Tnr/Mapt/Kcnma1/Lsamp/Kalrn/Fgf12/Cacna1a/Dab1/Fgf14/Nav2/Gnao1/Cntn1/Large1/Sptbn4/Cacna1b/Adcy5/Apba2/Cacnb4/Ube3a/Atp1a2/Sobp/Adam22/Htr2c/Reln/Rcan2/Grin2a/Atxn1/Elavl4/Cacna1c/Npas2/Prex2/Shank3/Sez6l/Alk/Gng7/Abl2/Pcdh15/Abat/Nrg1/Drd2/Shank2/Fign/Pde1b/Lrrk2/Adcy8/Pbx3/Apba1/Klhl1/Lgi4/Tmod1/Dscam/Agtpbp1/Prkce/Oprd1/Strn/Crhr1/Meis1/Slc18a2/Id2/Nr4a2/Zic1/Lepr/Mta1/Chrnb2/Ankfn1/Ccnd2/Uba6 |

**Table S10**. Table of target gene of miRNA in Exo in database: mirecords, mirtarbase, and tarbase.

| miRNA id | target gene | target ensembl | mirecords | mirtarbase | tarbase |
| --- | --- | --- | --- | --- | --- |
| hsa-miR-29a-3p | ZFP36 | ENSG00000128016 | 1 | 3 | 1 |
| hsa-miR-122-5p | XPO6 | ENSG00000169180 | 1 | 1 | 1 |
| hsa-miR-16-5p | WNT3A | ENSG00000154342 | 1 | 2 | 1 |
| hsa-miR-30a-3p | WDR82 | ENSG00000164091 | 1 | 1 | 1 |
| hsa-miR-181b-5p | VSNL1 | ENSG00000163032 | 1 | 1 | 1 |
| hsa-miR-16-5p | VPS45 | ENSG00000136631 | 1 | 1 | 1 |
| hsa-miR-210-3p | VMP1 | ENSG00000062716 | 1 | 2 | 1 |
| hsa-miR-30a-3p | VEZT | ENSG00000028203 | 1 | 1 | 1 |
| hsa-miR-16-5p | VEGFA | ENSG00000112715 | 1 | 14 | 2 |
| hsa-miR-17-5p | VEGFA | ENSG00000112715 | 1 | 1 | 2 |
| hsa-miR-93-5p | VEGFA | ENSG00000112715 | 1 | 2 | 1 |
| hsa-miR-34a-5p | VEGFA | ENSG00000112715 | 1 | 1 | 1 |
| hsa-miR-10a-5p | USF2 | ENSG00000105698 | 1 | 1 | 1 |
| hsa-miR-16-5p | UGDH | ENSG00000109814 | 1 | 2 | 1 |
| hsa-miR-30c-5p | UBE2I | ENSG00000103275 | 2 | 1 | 1 |
| hsa-miR-122-5p | UBAP2 | ENSG00000137073 | 1 | 1 | 1 |
| hsa-miR-128-3p | TXNIP | ENSG00000265972 | 1 | 3 | 1 |
| hsa-miR-30a-3p | TUBA1A | ENSG00000167552 | 1 | 1 | 1 |
| hsa-miR-17-5p | TSG101 | ENSG00000074319 | 1 | 3 | 2 |
| hsa-let-7a-5p | TRIM71 | ENSG00000206557 | 2 | 4 | 1 |
| hsa-let-7c-5p | TRIM71 | ENSG00000206557 | 1 | 3 | 1 |
| hsa-miR-122-5p | TRIB1 | ENSG00000173334 | 1 | 1 | 1 |
| hsa-miR-146a-5p | TRAF6 | ENSG00000175104 | 1 | 18 | 1 |
| hsa-miR-16-5p | TPPP3 | ENSG00000159713 | 1 | 1 | 1 |
| hsa-miR-21-5p | TPM1 | ENSG00000140416 | 1 | 5 | 2 |
| hsa-miR-122-5p | TPD52L2 | ENSG00000101150 | 1 | 2 | 1 |
| hsa-miR-21-5p | TP63 | ENSG00000073282 | 1 | 1 | 1 |
| hsa-miR-125b-5p | TP53INP1 | ENSG00000164938 | 1 | 5 | 1 |
| hsa-miR-21-5p | TP53BP2 | ENSG00000143514 | 1 | 1 | 1 |
| hsa-miR-125a-5p | TP53 | ENSG00000141510 | 2 | 2 | 1 |
| hsa-miR-21-5p | TOPORS | ENSG00000197579 | 1 | 2 | 1 |
| hsa-let-7i-5p | TLR4 | ENSG00000136869 | 1 | 1 | 1 |
| hsa-miR-21-5p | TIMP3 | ENSG00000100234 | 1 | 5 | 2 |
| hsa-miR-221-3p | TIMP3 | ENSG00000100234 | 1 | 4 | 1 |
| hsa-miR-222-3p | TIMP3 | ENSG00000100234 | 1 | 4 | 1 |
| hsa-miR-19a-3p | THBS1 | ENSG00000137801 | 1 | 1 | 1 |
| hsa-miR-30a-3p | THBS1 | ENSG00000137801 | 1 | 1 | 1 |
| hsa-miR-21-5p | TGFBR3 | ENSG00000069702 | 1 | 2 | 1 |
| hsa-miR-21-5p | TGFBR2 | ENSG00000163513 | 2 | 7 | 1 |
| hsa-let-7c-5p | TGFBR1 | ENSG00000106799 | 2 | 1 | 1 |
| hsa-miR-125b-5p | TDG | ENSG00000139372 | 1 | 1 | 1 |
| hsa-miR-29c-3p | TDG | ENSG00000139372 | 1 | 4 | 1 |
| hsa-miR-29b-3p | TCL1A | ENSG00000100721 | 1 | 2 | 1 |
| hsa-miR-181b-5p | TCL1A | ENSG00000100721 | 1 | 1 | 1 |
| hsa-miR-122-5p | TBX19 | ENSG00000143178 | 1 | 1 | 1 |
| hsa-miR-221-3p | TBK1 | ENSG00000183735 | 1 | 1 | 1 |
| hsa-miR-130a-3p | TAC1 | ENSG00000006128 | 1 | 1 | 1 |
| hsa-miR-222-3p | STAT5A | ENSG00000126561 | 1 | 2 | 1 |
| hsa-miR-21-5p | STAT3 | ENSG00000168610 | 1 | 4 | 1 |
| hsa-miR-146a-5p | STAT1 | ENSG00000115415 | 2 | 5 | 1 |
| hsa-miR-29a-3p | SPARC | ENSG00000113140 | 1 | 5 | 1 |
| hsa-miR-27a-3p | SP4 | ENSG00000105866 | 1 | 2 | 1 |
| hsa-miR-27a-3p | SP3 | ENSG00000172845 | 1 | 2 | 1 |
| hsa-miR-27a-3p | SP1 | ENSG00000185591 | 1 | 4 | 1 |
| hsa-miR-29b-3p | SP1 | ENSG00000185591 | 2 | 5 | 1 |
| hsa-miR-21-5p | SOCS5 | ENSG00000171150 | 1 | 1 | 1 |
| hsa-miR-19a-3p | SOCS1 | ENSG00000185338 | 1 | 5 | 1 |
| hsa-miR-19b-3p | SOCS1 | ENSG00000185338 | 1 | 4 | 1 |
| hsa-miR-7-5p | SNCA | ENSG00000145335 | 1 | 2 | 1 |
| hsa-miR-125b-5p | SMO | ENSG00000128602 | 1 | 2 | 1 |
| hsa-miR-324-5p | SMO | ENSG00000128602 | 1 | 2 | 1 |
| hsa-let-7e-5p | SMC1A | ENSG00000072501 | 2 | 5 | 1 |
| hsa-miR-30a-3p | SLC7A6 | ENSG00000103064 | 1 | 1 | 1 |
| hsa-miR-122-5p | SLC7A11 | ENSG00000151012 | 1 | 1 | 1 |
| hsa-miR-122-5p | SLC7A1 | ENSG00000139514 | 2 | 4 | 1 |
| hsa-miR-126-5p | SLC45A3 | ENSG00000158715 | 2 | 1 | 1 |
| hsa-miR-21-5p | SLC16A10 | ENSG00000112394 | 1 | 1 | 1 |
| hsa-miR-16-5p | SKAP2 | ENSG00000005020 | 1 | 1 | 1 |
| hsa-miR-34a-5p | SIRT1 | ENSG00000096717 | 1 | 21 | 2 |
| hsa-miR-125b-5p | SGPL1 | ENSG00000166224 | 1 | 2 | 1 |
| hsa-miR-29b-3p | SFPQ | ENSG00000116560 | 1 | 1 | 1 |
| hsa-miR-21-5p | SESN1 | ENSG00000080546 | 1 | 1 | 1 |
| hsa-miR-21-5p | SERPINB5 | ENSG00000206075 | 1 | 6 | 2 |
| hsa-miR-17-5p | RUNX1 | ENSG00000159216 | 2 | 1 | 1 |
| hsa-miR-27a-3p | RUNX1 | ENSG00000159216 | 2 | 1 | 2 |
| hsa-miR-145-5p | RTKN | ENSG00000114993 | 1 | 1 | 2 |
| hsa-miR-21-5p | RP2 | ENSG00000102218 | 2 | 1 | 1 |
| hsa-miR-138-5p | ROCK2 | ENSG00000134318 | 1 | 1 | 1 |
| hsa-miR-138-5p | RHOC | ENSG00000155366 | 1 | 6 | 1 |
| hsa-miR-128-3p | RELN | ENSG00000189056 | 1 | 1 | 1 |
| hsa-miR-16-5p | RECK | ENSG00000122707 | 2 | 3 | 1 |
| hsa-miR-21-5p | RECK | ENSG00000122707 | 3 | 10 | 1 |
| hsa-miR-17-5p | RBL1 | ENSG00000080839 | 1 | 1 | 2 |
| hsa-miR-21-5p | RASGRP1 | ENSG00000172575 | 1 | 3 | 1 |
| hsa-miR-7-5p | RAF1 | ENSG00000132155 | 4 | 3 | 1 |
| hsa-miR-16-5p | RAD51C | ENSG00000108384 | 1 | 1 | 1 |
| hsa-miR-122-5p | RAD21 | ENSG00000164754 | 1 | 3 | 1 |
| hsa-miR-122-5p | RAB6B | ENSG00000154917 | 1 | 1 | 1 |
| hsa-miR-122-5p | RAB11FIP1 | ENSG00000156675 | 1 | 2 | 1 |
| hsa-miR-19a-3p | PTEN | ENSG00000171862 | 6 | 9 | 1 |
| hsa-miR-21-5p | PTEN | ENSG00000171862 | 4 | 62 | 2 |
| hsa-miR-22-3p | PTEN | ENSG00000171862 | 2 | 2 | 1 |
| hsa-miR-29a-3p | PTEN | ENSG00000171862 | 4 | 5 | 1 |
| hsa-miR-214-3p | PTEN | ENSG00000171862 | 2 | 8 | 1 |
| hsa-miR-221-3p | PTEN | ENSG00000171862 | 2 | 7 | 1 |
| hsa-miR-125b-5p | PRKRA | ENSG00000180228 | 1 | 1 | 1 |
| hsa-miR-16-5p | PRIM1 | ENSG00000198056 | 1 | 1 | 2 |
| hsa-let-7a-5p | PRDM1 | ENSG00000057657 | 1 | 2 | 1 |
| hsa-miR-145-5p | PPP3CA | ENSG00000138814 | 1 | 1 | 1 |
| hsa-miR-31-5p | PPP2R2A | ENSG00000221914 | 1 | 1 | 1 |
| hsa-miR-222-3p | PPP2R2A | ENSG00000221914 | 1 | 5 | 1 |
| hsa-miR-125b-5p | PPP2CA | ENSG00000113575 | 1 | 1 | 1 |
| hsa-miR-125b-5p | PPP1CA | ENSG00000172531 | 1 | 3 | 1 |
| hsa-miR-21-5p | PPIF | ENSG00000108179 | 1 | 2 | 1 |
| hsa-miR-21-5p | PPARA | ENSG00000186951 | 1 | 4 | 1 |
| hsa-miR-16-5p | PNN | ENSG00000100941 | 1 | 1 | 1 |
| hsa-miR-16-5p | PMS1 | ENSG00000064933 | 2 | 1 | 1 |
| hsa-miR-23b-3p | PLAU | ENSG00000122861 | 2 | 2 | 2 |
| hsa-miR-193b-3p | PLAU | ENSG00000122861 | 1 | 3 | 1 |
| hsa-miR-424-5p | PLAG1 | ENSG00000181690 | 1 | 6 | 1 |
| hsa-miR-29a-3p | PIK3R1 | ENSG00000145675 | 1 | 1 | 1 |
| hsa-miR-29b-3p | PIK3R1 | ENSG00000145675 | 1 | 2 | 1 |
| hsa-miR-16-5p | PHKB | ENSG00000102893 | 1 | 1 | 1 |
| hsa-miR-27a-3p | PHB | ENSG00000167085 | 1 | 5 | 2 |
| hsa-miR-21-5p | PELI1 | ENSG00000197329 | 1 | 4 | 1 |
| hsa-miR-16-5p | PDCD4 | ENSG00000150593 | 1 | 2 | 1 |
| hsa-miR-21-5p | PDCD4 | ENSG00000150593 | 9 | 50 | 2 |
| hsa-miR-7-5p | PAK1 | ENSG00000149269 | 1 | 3 | 1 |
| hsa-miR-16-5p | OMA1 | ENSG00000162600 | 1 | 1 | 1 |
| hsa-miR-122-5p | NUMBL | ENSG00000105245 | 1 | 1 | 1 |
| hsa-miR-125b-5p | NTRK3 | ENSG00000140538 | 1 | 1 | 1 |
| hsa-miR-128-3p | NTRK3 | ENSG00000140538 | 1 | 2 | 1 |
| hsa-miR-485-3p | NTRK3 | ENSG00000140538 | 1 | 1 | 2 |
| hsa-miR-16-5p | NT5DC1 | ENSG00000178425 | 1 | 1 | 1 |
| hsa-let-7a-5p | NRAS | ENSG00000213281 | 18 | 2 | 1 |
| hsa-miR-19a-3p | NR4A2 | ENSG00000153234 | 1 | 1 | 1 |
| hsa-miR-34a-5p | NOTCH2 | ENSG00000134250 | 2 | 2 | 2 |
| hsa-miR-24-3p | NOTCH1 | ENSG00000148400 | 1 | 1 | 1 |
| hsa-miR-34a-5p | NOTCH1 | ENSG00000148400 | 7 | 15 | 2 |
| hsa-miR-23b-3p | NOTCH1 | ENSG00000148400 | 1 | 1 | 1 |
| hsa-miR-181b-5p | NLK | ENSG00000087095 | 1 | 1 | 1 |
| hsa-miR-21-5p | NFIB | ENSG00000147862 | 1 | 1 | 1 |
| hsa-miR-122-5p | NFATC2IP | ENSG00000176953 | 1 | 1 | 1 |
| hsa-let-7a-5p | NF2 | ENSG00000186575 | 2 | 1 | 1 |
| hsa-miR-17-5p | NCOA3 | ENSG00000124151 | 3 | 3 | 2 |
| hsa-miR-122-5p | NCAM1 | ENSG00000149294 | 2 | 3 | 1 |
| hsa-let-7c-5p | MYC | ENSG00000136997 | 1 | 2 | 1 |
| hsa-miR-24-3p | MYC | ENSG00000136997 | 6 | 2 | 1 |
| hsa-miR-34a-5p | MYC | ENSG00000136997 | 1 | 7 | 1 |
| hsa-miR-145-5p | MYC | ENSG00000136997 | 1 | 5 | 1 |
| hsa-miR-34a-5p | MYB | ENSG00000118513 | 3 | 3 | 1 |
| hsa-miR-150-5p | MYB | ENSG00000118513 | 4 | 12 | 1 |
| hsa-miR-145-5p | MUC1 | ENSG00000185499 | 1 | 4 | 1 |
| hsa-miR-100-5p | MTOR | ENSG00000198793 | 1 | 5 | 2 |
| hsa-miR-199a-3p | MTOR | ENSG00000198793 | 1 | 4 | 1 |
| hsa-miR-16-5p | MSH2 | ENSG00000095002 | 2 | 1 | 1 |
| hsa-miR-146a-5p | METTL7A | ENSG00000185432 | 1 | 1 | 1 |
| hsa-miR-34a-5p | MET | ENSG00000105976 | 2 | 15 | 2 |
| hsa-miR-23b-3p | MET | ENSG00000105976 | 4 | 3 | 1 |
| hsa-miR-130a-3p | MEOX2 | ENSG00000106511 | 1 | 1 | 1 |
| hsa-miR-17-5p | MEF2D | ENSG00000116604 | 1 | 1 | 1 |
| hsa-miR-16-5p | MCL1 | ENSG00000143384 | 1 | 1 | 1 |
| hsa-miR-29a-3p | MCL1 | ENSG00000143384 | 1 | 9 | 1 |
| hsa-miR-29b-3p | MCL1 | ENSG00000143384 | 2 | 15 | 1 |
| hsa-miR-22-3p | MAX | ENSG00000125952 | 1 | 1 | 1 |
| hsa-miR-17-5p | MAPK9 | ENSG00000050748 | 1 | 3 | 2 |
| hsa-miR-143-3p | MAPK7 | ENSG00000166484 | 2 | 14 | 1 |
| hsa-miR-24-3p | MAPK14 | ENSG00000112062 | 1 | 1 | 1 |
| hsa-miR-122-5p | MAPK11 | ENSG00000185386 | 1 | 1 | 1 |
| hsa-miR-17-5p | MAP3K12 | ENSG00000139625 | 1 | 1 | 1 |
| hsa-miR-34a-5p | MAP2K1 | ENSG00000169032 | 1 | 1 | 2 |
| hsa-miR-424-5p | MAP2K1 | ENSG00000169032 | 1 | 2 | 1 |
| hsa-miR-130a-3p | MAFB | ENSG00000204103 | 1 | 1 | 2 |
| hsa-miR-128-3p | LDLR | ENSG00000130164 | 2 | 2 | 1 |
| hsa-miR-31-5p | LATS2 | ENSG00000150457 | 1 | 1 | 1 |
| hsa-miR-29c-3p | LAMC1 | ENSG00000135862 | 1 | 1 | 1 |
| hsa-miR-145-5p | KRT7 | ENSG00000135480 | 1 | 1 | 1 |
| hsa-miR-143-3p | KRAS | ENSG00000133703 | 2 | 4 | 1 |
| hsa-miR-221-3p | KIT | ENSG00000157404 | 2 | 8 | 1 |
| hsa-miR-222-3p | KIT | ENSG00000157404 | 1 | 6 | 1 |
| hsa-miR-146a-5p | KIF22 | ENSG00000079616 | 1 | 1 | 1 |
| hsa-miR-16-5p | JUN | ENSG00000177606 | 1 | 1 | 2 |
| hsa-miR-21-5p | JMY | ENSG00000152409 | 1 | 1 | 1 |
| hsa-miR-17-5p | JAK1 | ENSG00000162434 | 1 | 2 | 1 |
| hsa-miR-21-5p | JAG1 | ENSG00000101384 | 1 | 3 | 1 |
| hsa-miR-34a-5p | JAG1 | ENSG00000101384 | 1 | 3 | 1 |
| hsa-miR-92a-3p | ITGA5 | ENSG00000161638 | 1 | 1 | 1 |
| hsa-miR-7-5p | IRS2 | ENSG00000185950 | 3 | 1 | 1 |
| hsa-miR-7-5p | IRS1 | ENSG00000169047 | 1 | 1 | 1 |
| hsa-miR-145-5p | IRS1 | ENSG00000169047 | 2 | 8 | 1 |
| hsa-miR-146a-5p | IRAK1 | ENSG00000184216 | 2 | 22 | 1 |
| hsa-miR-19a-3p | IKZF1 | ENSG00000185811 | 1 | 1 | 1 |
| hsa-miR-19b-3p | IKZF1 | ENSG00000185811 | 1 | 1 | 1 |
| hsa-miR-27a-3p | IKZF1 | ENSG00000185811 | 1 | 1 | 1 |
| hsa-miR-92a-3p | IKZF1 | ENSG00000185811 | 1 | 1 | 1 |
| hsa-miR-34a-5p | IKZF1 | ENSG00000185811 | 1 | 1 | 1 |
| hsa-let-7g-5p | IGF2BP1 | ENSG00000159217 | 1 | 3 | 1 |
| hsa-miR-221-3p | ICAM1 | ENSG00000090339 | 1 | 3 | 1 |
| hsa-miR-16-5p | HSPA1A | ENSG00000204389 | 1 | 2 | 1 |
| hsa-miR-16-5p | HSP90B1 | ENSG00000166598 | 1 | 1 | 1 |
| hsa-miR-16-5p | HSDL2 | ENSG00000119471 | 1 | 1 | 1 |
| hsa-miR-196a-5p | HOXD8 | ENSG00000175879 | 1 | 2 | 1 |
| hsa-miR-196a-5p | HOXC8 | ENSG00000037965 | 2 | 11 | 1 |
| hsa-miR-196a-5p | HOXB8 | ENSG00000120068 | 2 | 6 | 1 |
| hsa-miR-196a-5p | HOXA7 | ENSG00000122592 | 4 | 4 | 1 |
| hsa-miR-130a-3p | HOXA5 | ENSG00000106004 | 1 | 2 | 1 |
| hsa-miR-10a-5p | HOXA1 | ENSG00000105991 | 1 | 2 | 1 |
| hsa-miR-21-5p | HNRNPK | ENSG00000165119 | 2 | 1 | 1 |
| hsa-let-7a-5p | HMGA2 | ENSG00000149948 | 7 | 13 | 1 |
| hsa-let-7b-5p | HMGA2 | ENSG00000149948 | 6 | 7 | 1 |
| hsa-let-7c-5p | HMGA2 | ENSG00000149948 | 8 | 8 | 1 |
| hsa-let-7e-5p | HMGA2 | ENSG00000149948 | 6 | 4 | 1 |
| hsa-miR-98-5p | HMGA2 | ENSG00000149948 | 7 | 6 | 1 |
| hsa-let-7g-5p | HMGA2 | ENSG00000149948 | 7 | 7 | 1 |
| hsa-miR-185-5p | HMGA2 | ENSG00000149948 | 1 | 1 | 1 |
| hsa-miR-16-5p | HMGA1 | ENSG00000137309 | 1 | 3 | 1 |
| hsa-miR-21-5p | HIPK3 | ENSG00000110422 | 1 | 1 | 1 |
| hsa-miR-16-5p | HDHD2 | ENSG00000167220 | 1 | 1 | 1 |
| hsa-miR-122-5p | GYS1 | ENSG00000104812 | 1 | 2 | 1 |
| hsa-miR-16-5p | GTF2H1 | ENSG00000110768 | 1 | 1 | 1 |
| hsa-miR-181b-5p | GRIA2 | ENSG00000120251 | 1 | 1 | 1 |
| hsa-miR-16-5p | GOLGA5 | ENSG00000066455 | 1 | 1 | 1 |
| hsa-miR-324-5p | GLI1 | ENSG00000111087 | 1 | 2 | 1 |
| hsa-miR-21-5p | GLCCI1 | ENSG00000106415 | 1 | 2 | 1 |
| hsa-miR-181b-5p | GATA6 | ENSG00000141448 | 1 | 2 | 1 |
| hsa-miR-122-5p | GALNT10 | ENSG00000164574 | 1 | 2 | 1 |
| hsa-miR-17-5p | GAB1 | ENSG00000109458 | 1 | 1 | 1 |
| hsa-miR-122-5p | G6PC3 | ENSG00000141349 | 2 | 2 | 1 |
| hsa-miR-122-5p | FUNDC2 | ENSG00000165775 | 2 | 1 | 1 |
| hsa-miR-145-5p | FSCN1 | ENSG00000075618 | 4 | 12 | 1 |
| hsa-miR-122-5p | FOXP1 | ENSG00000114861 | 1 | 1 | 1 |
| hsa-miR-27a-3p | FOXO1 | ENSG00000150907 | 1 | 4 | 2 |
| hsa-miR-122-5p | FOXJ3 | ENSG00000198815 | 1 | 1 | 1 |
| hsa-miR-221-3p | FOS | ENSG00000170345 | 1 | 3 | 1 |
| hsa-miR-222-3p | FOS | ENSG00000170345 | 1 | 3 | 1 |
| hsa-miR-143-3p | FNDC3B | ENSG00000075420 | 1 | 1 | 1 |
| hsa-miR-100-5p | FGFR3 | ENSG00000068078 | 1 | 5 | 2 |
| hsa-miR-16-5p | FGFR1 | ENSG00000077782 | 2 | 1 | 1 |
| hsa-miR-424-5p | FGFR1 | ENSG00000077782 | 2 | 3 | 1 |
| hsa-miR-24-3p | FEN1 | ENSG00000168496 | 1 | 1 | 1 |
| hsa-miR-25-3p | FBXW7 | ENSG00000109670 | 1 | 3 | 1 |
| hsa-miR-27a-3p | FBXW7 | ENSG00000109670 | 2 | 3 | 1 |
| hsa-miR-29c-3p | FBN1 | ENSG00000166147 | 1 | 2 | 1 |
| hsa-miR-21-5p | FAS | ENSG00000026103 | 2 | 1 | 1 |
| hsa-miR-21-5p | FAM3C | ENSG00000196937 | 1 | 1 | 1 |
| hsa-miR-146a-5p | FADD | ENSG00000168040 | 2 | 1 | 1 |
| hsa-miR-19a-3p | ESR1 | ENSG00000091831 | 1 | 1 | 1 |
| hsa-miR-22-3p | ESR1 | ENSG00000091831 | 2 | 2 | 2 |
| hsa-miR-221-3p | ESR1 | ENSG00000091831 | 1 | 2 | 2 |
| hsa-miR-193b-3p | ESR1 | ENSG00000091831 | 1 | 1 | 1 |
| hsa-miR-19a-3p | ERBB4 | ENSG00000178568 | 1 | 1 | 2 |
| hsa-miR-125b-5p | ERBB3 | ENSG00000065361 | 1 | 3 | 1 |
| hsa-miR-125a-5p | ERBB3 | ENSG00000065361 | 1 | 3 | 1 |
| hsa-miR-125b-5p | ERBB2 | ENSG00000141736 | 1 | 8 | 1 |
| hsa-miR-125a-5p | ERBB2 | ENSG00000141736 | 1 | 4 | 1 |
| hsa-miR-122-5p | ENTPD4 | ENSG00000197217 | 1 | 1 | 1 |
| hsa-let-7d-5p | EIF4G2 | ENSG00000110321 | 1 | 3 | 1 |
| hsa-let-7g-5p | EIF4G2 | ENSG00000110321 | 1 | 3 | 1 |
| hsa-let-7i-5p | EIF4G2 | ENSG00000110321 | 1 | 3 | 1 |
| hsa-miR-150-5p | EGR2 | ENSG00000122877 | 2 | 2 | 1 |
| hsa-miR-122-5p | EGLN3 | ENSG00000129521 | 1 | 1 | 1 |
| hsa-miR-7-5p | EGFR | ENSG00000146648 | 6 | 13 | 2 |
| hsa-miR-210-3p | EFNA3 | ENSG00000143590 | 1 | 8 | 1 |
| hsa-miR-16-5p | ECHDC1 | ENSG00000093144 | 1 | 1 | 1 |
| hsa-miR-34a-5p | E2F3 | ENSG00000112242 | 2 | 12 | 1 |
| hsa-miR-125b-5p | E2F3 | ENSG00000112242 | 1 | 2 | 1 |
| hsa-miR-24-3p | E2F2 | ENSG00000007968 | 1 | 1 | 1 |
| hsa-miR-93-5p | E2F1 | ENSG00000101412 | 2 | 4 | 1 |
| hsa-miR-122-5p | DUSP2 | ENSG00000158050 | 1 | 1 | 1 |
| hsa-miR-29a-3p | DNMT3B | ENSG00000088305 | 6 | 3 | 1 |
| hsa-miR-29b-3p | DNMT3B | ENSG00000088305 | 4 | 9 | 1 |
| hsa-miR-29c-3p | DNMT3B | ENSG00000088305 | 2 | 1 | 1 |
| hsa-miR-29a-3p | DNMT3A | ENSG00000119772 | 1 | 3 | 1 |
| hsa-miR-29b-3p | DNMT3A | ENSG00000119772 | 2 | 8 | 1 |
| hsa-miR-29c-3p | DNMT3A | ENSG00000119772 | 1 | 5 | 1 |
| hsa-miR-342-3p | DNMT1 | ENSG00000130816 | 2 | 2 | 1 |
| hsa-miR-29b-3p | DNAJB11 | ENSG00000090520 | 1 | 1 | 1 |
| hsa-miR-34a-5p | DLL1 | ENSG00000198719 | 3 | 4 | 2 |
| hsa-let-7a-5p | DICER1 | ENSG00000100697 | 2 | 2 | 1 |
| hsa-let-7d-5p | DICER1 | ENSG00000100697 | 2 | 1 | 1 |
| hsa-miR-130a-3p | DDX6 | ENSG00000110367 | 2 | 1 | 1 |
| hsa-miR-221-3p | DDIT4 | ENSG00000168209 | 2 | 2 | 1 |
| hsa-miR-146a-5p | CXCR4 | ENSG00000121966 | 4 | 3 | 1 |
| hsa-miR-23a-3p | CXCL12 | ENSG00000107562 | 3 | 1 | 1 |
| hsa-miR-130a-3p | CSF1 | ENSG00000184371 | 3 | 2 | 1 |
| hsa-miR-16-5p | CREBL2 | ENSG00000111269 | 1 | 3 | 1 |
| hsa-miR-103a-3p | CREB1 | ENSG00000118260 | 1 | 1 | 1 |
| hsa-miR-29c-3p | COL4A2 | ENSG00000134871 | 2 | 2 | 1 |
| hsa-miR-29c-3p | COL4A1 | ENSG00000187498 | 1 | 6 | 1 |
| hsa-miR-29a-3p | COL3A1 | ENSG00000168542 | 2 | 2 | 1 |
| hsa-miR-29b-3p | COL3A1 | ENSG00000168542 | 2 | 4 | 1 |
| hsa-miR-29c-3p | COL3A1 | ENSG00000168542 | 6 | 3 | 1 |
| hsa-miR-29c-3p | COL1A2 | ENSG00000164692 | 2 | 3 | 1 |
| hsa-miR-29b-3p | COL1A1 | ENSG00000108821 | 2 | 8 | 2 |
| hsa-miR-29c-3p | COL1A1 | ENSG00000108821 | 6 | 2 | 1 |
| hsa-miR-29c-3p | COL15A1 | ENSG00000204291 | 1 | 1 | 1 |
| hsa-miR-145-5p | CLINT1 | ENSG00000113282 | 1 | 1 | 1 |
| hsa-miR-122-5p | CLIC4 | ENSG00000169504 | 1 | 4 | 1 |
| hsa-miR-16-5p | CFL2 | ENSG00000165410 | 2 | 1 | 1 |
| hsa-miR-16-5p | CENPJ | ENSG00000151849 | 1 | 1 | 1 |
| hsa-miR-125b-5p | CEBPG | ENSG00000153879 | 1 | 1 | 1 |
| hsa-miR-181b-5p | CDX2 | ENSG00000165556 | 1 | 1 | 1 |
| hsa-miR-24-3p | CDKN2A | ENSG00000147889 | 2 | 1 | 1 |
| hsa-miR-221-3p | CDKN1C | ENSG00000129757 | 3 | 5 | 1 |
| hsa-miR-222-3p | CDKN1C | ENSG00000129757 | 2 | 4 | 1 |
| hsa-miR-221-3p | CDKN1B | ENSG00000111276 | 11 | 28 | 1 |
| hsa-miR-222-3p | CDKN1B | ENSG00000111276 | 9 | 23 | 1 |
| hsa-miR-17-5p | CDKN1A | ENSG00000124762 | 1 | 11 | 1 |
| hsa-miR-93-5p | CDKN1A | ENSG00000124762 | 2 | 6 | 1 |
| hsa-let-7b-5p | CDK6 | ENSG00000105810 | 1 | 1 | 1 |
| hsa-miR-16-5p | CDK6 | ENSG00000105810 | 1 | 2 | 1 |
| hsa-miR-29a-3p | CDK6 | ENSG00000105810 | 1 | 7 | 1 |
| hsa-miR-30a-3p | CDK6 | ENSG00000105810 | 1 | 1 | 1 |
| hsa-miR-29b-3p | CDK6 | ENSG00000105810 | 3 | 7 | 1 |
| hsa-miR-34a-5p | CDK6 | ENSG00000105810 | 1 | 8 | 2 |
| hsa-miR-424-5p | CDK6 | ENSG00000105810 | 1 | 2 | 1 |
| hsa-miR-24-3p | CDK4 | ENSG00000135446 | 2 | 1 | 1 |
| hsa-miR-103a-3p | CDK2 | ENSG00000123374 | 1 | 1 | 1 |
| hsa-miR-29a-3p | CDC42 | ENSG00000070831 | 2 | 3 | 1 |
| hsa-miR-29b-3p | CDC42 | ENSG00000070831 | 2 | 2 | 1 |
| hsa-miR-29c-3p | CDC42 | ENSG00000070831 | 2 | 2 | 1 |
| hsa-let-7b-5p | CDC34 | ENSG00000099804 | 1 | 3 | 1 |
| hsa-let-7b-5p | CDC25A | ENSG00000164045 | 2 | 2 | 1 |
| hsa-miR-21-5p | CDC25A | ENSG00000164045 | 1 | 5 | 1 |
| hsa-miR-34a-5p | CD44 | ENSG00000026508 | 2 | 6 | 2 |
| hsa-miR-328-3p | CD44 | ENSG00000026508 | 1 | 3 | 1 |
| hsa-miR-29a-3p | CD276 | ENSG00000103855 | 1 | 2 | 1 |
| hsa-miR-122-5p | CCNG1 | ENSG00000113328 | 1 | 5 | 1 |
| hsa-miR-16-5p | CCNE1 | ENSG00000105173 | 5 | 11 | 1 |
| hsa-miR-103a-3p | CCNE1 | ENSG00000105173 | 1 | 1 | 1 |
| hsa-miR-424-5p | CCNE1 | ENSG00000105173 | 1 | 5 | 1 |
| hsa-miR-16-5p | CCND3 | ENSG00000112576 | 1 | 1 | 2 |
| hsa-miR-424-5p | CCND3 | ENSG00000112576 | 1 | 1 | 1 |
| hsa-let-7b-5p | CCND1 | ENSG00000110092 | 1 | 5 | 1 |
| hsa-miR-16-5p | CCND1 | ENSG00000110092 | 3 | 7 | 1 |
| hsa-miR-17-5p | CCND1 | ENSG00000110092 | 2 | 10 | 2 |
| hsa-miR-19a-3p | CCND1 | ENSG00000110092 | 1 | 1 | 1 |
| hsa-miR-34a-5p | CCND1 | ENSG00000110092 | 1 | 5 | 2 |
| hsa-miR-424-5p | CCND1 | ENSG00000110092 | 1 | 3 | 1 |
| hsa-miR-24-3p | CCNA2 | ENSG00000145386 | 1 | 1 | 1 |
| hsa-miR-421 | CBX7 | ENSG00000100307 | 1 | 1 | 1 |
| hsa-miR-145-5p | CBFB | ENSG00000067955 | 1 | 2 | 2 |
| hsa-let-7a-5p | CASP3 | ENSG00000164305 | 1 | 1 | 1 |
| hsa-miR-16-5p | CAPRIN1 | ENSG00000135387 | 1 | 1 | 1 |
| hsa-miR-16-5p | CADM1 | ENSG00000182985 | 1 | 1 | 1 |
| hsa-miR-16-5p | C17orf80 | ENSG00000141219 | 1 | 1 | 1 |
| hsa-miR-21-5p | BTG2 | ENSG00000159388 | 2 | 6 | 1 |
| hsa-miR-24-3p | BRCA1 | ENSG00000012048 | 2 | 2 | 1 |
| hsa-miR-221-3p | BNIP3L | ENSG00000104765 | 1 | 1 | 1 |
| hsa-miR-17-5p | BMPR2 | ENSG00000204217 | 2 | 3 | 1 |
| hsa-miR-21-5p | BMPR2 | ENSG00000204217 | 6 | 4 | 1 |
| hsa-miR-128-3p | BMI1 | ENSG00000168283 | 1 | 6 | 1 |
| hsa-miR-708-5p | BMI1 | ENSG00000168283 | 1 | 1 | 1 |
| hsa-miR-122-5p | BCL2L2 | ENSG00000129473 | 1 | 3 | 1 |
| hsa-miR-17-5p | BCL2L11 | ENSG00000153094 | 1 | 9 | 2 |
| hsa-miR-19a-3p | BCL2L11 | ENSG00000153094 | 1 | 1 | 1 |
| hsa-miR-19b-3p | BCL2L11 | ENSG00000153094 | 1 | 2 | 1 |
| hsa-miR-16-5p | BCL2 | ENSG00000171791 | 7 | 17 | 1 |
| hsa-miR-17-5p | BCL2 | ENSG00000171791 | 1 | 2 | 2 |
| hsa-miR-34a-5p | BCL2 | ENSG00000171791 | 4 | 16 | 2 |
| hsa-miR-221-3p | BBC3 | ENSG00000105327 | 1 | 5 | 1 |
| hsa-miR-125b-5p | BAK1 | ENSG00000030110 | 2 | 5 | 1 |
| hsa-miR-29a-3p | BACE1 | ENSG00000186318 | 1 | 1 | 1 |
| hsa-miR-34a-5p | AXL | ENSG00000167601 | 1 | 4 | 1 |
| hsa-miR-34a-5p | AXIN2 | ENSG00000168646 | 4 | 1 | 1 |
| hsa-miR-24-3p | AURKB | ENSG00000178999 | 1 | 1 | 1 |
| hsa-miR-19a-3p | ATXN1 | ENSG00000124788 | 1 | 2 | 1 |
| hsa-miR-130a-3p | ATXN1 | ENSG00000124788 | 1 | 1 | 1 |
| hsa-miR-122-5p | ATP1A2 | ENSG00000018625 | 2 | 1 | 1 |
| hsa-miR-16-5p | ASXL2 | ENSG00000143970 | 1 | 1 | 1 |
| hsa-miR-224-5p | API5 | ENSG00000166181 | 3 | 1 | 1 |
| hsa-miR-21-5p | APAF1 | ENSG00000120868 | 2 | 3 | 1 |
| hsa-miR-122-5p | AP3M2 | ENSG00000070718 | 1 | 1 | 1 |
| hsa-miR-224-5p | AP2M1 | ENSG00000161203 | 1 | 1 | 1 |
| hsa-miR-122-5p | ANXA11 | ENSG00000122359 | 1 | 1 | 1 |
| hsa-miR-196a-5p | ANXA1 | ENSG00000135046 | 1 | 2 | 1 |
| hsa-miR-122-5p | ANK2 | ENSG00000145362 | 1 | 1 | 1 |
| hsa-miR-122-5p | ALDOA | ENSG00000149925 | 2 | 2 | 1 |
| hsa-miR-122-5p | AKT3 | ENSG00000117020 | 1 | 2 | 1 |
| hsa-miR-128-3p | ADORA2B | ENSG00000170425 | 1 | 2 | 1 |
| hsa-miR-122-5p | ADAM17 | ENSG00000151694 | 1 | 3 | 1 |
| hsa-miR-24-3p | ACVR1B | ENSG00000135503 | 2 | 3 | 1 |
| hsa-miR-328-3p | ABCG2 | ENSG00000118777 | 1 | 3 | 1 |
| hsa-miR-122-5p | AACS | ENSG00000081760 | 1 | 1 | 1 |

**Table S11**. Table of GSEA GO biological process enrichment results of target gene for miRNA.

| Description | geneID |
| --- | --- |
| positive regulation of kinase activity | WNT3A/FBXW7/SIRT1/VAV3/RASGRP1/SOCS1/AXIN2/TCL1A/HMGA2/MAP3K12/VEGFA/TRAF6/TLR4/THBS1/TGFBR2/ADAM17/SNCA/RELN/MAP2K1/PPP2CA/PAK1/NTRK3/MET/KIT/IRS1/IRAK1/IL6R/MTOR/FGFR3/FGFR1/ERBB4/ERBB3/ERBB2/EPHA5/EGFR/EFNA5/CSF1/CLU/CDKN1B/CDKN1A/CDC42/CCND3/BMPR2/CCND1/AXL/RHOA |
| epithelial cell proliferation | WNT3A/FBXW7/UHRF1/SIRT1/AKT3/TP63/ZFP36/VEGFA/THBS1/TGFBR3/TGFBR1/ADAM17/STAT5A/STAT3/STAT1/SPARC/SP1/SMO/CXCL12/PTEN/MAP2K1/SERPINB5/NRAS/NOTCH2/NOTCH1/NFIB/MYC/LAMC1/KIT/JUN/ID1/HOXA5/GLI1/FGFR1/ESR1/ERBB2/EGFR/CDKN1C/CDKN1B/CDK6/CDC42/BRCA2/BMPR2/BCL2L2/CCND1 |
| response to peptide | BACE1/SIRT1/BCL2L11/ROCK2/IRS2/SOCS1/BTG2/WNT1/VIM/TP53/TLR4/TGFBR3/STAT5A/STAT3/STAT1/SP1/CXCL12/PPARA/POU4F2/PIK3R1/NR4A2/NOTCH1/MYC/MAX/JAK1/IRS1/ID1/ICAM1/GAB1/MTOR/FOXO1/FBN1/EGR2/MAPK14/CREB1/COL3A1/COL1A1/CDKN1B/CDK4/CCND3/CCNA2/BCL2L2/ASS1/ANXA1/JAG1 |
| myeloid cell differentiation | FBXW7/DLL1/FOXP1/SIRT1/IKZF1/TRIB1/MAFB/FADD/ZFP36/VEGFA/TRAF6/TLR4/TGFBR3/TGFBR2/STAT3/STAT1/SP3/POU4F2/PIK3R1/NOTCH2/MYC/LIF/KIT/JUN/HSPA1A/HOXB8/HOXA7/HOXA5/MTOR/FOS/FBN1/CSF1/MAPK14/CREB1/CEBPG/CDKN1C/CDK6/CDC42/CBFB/RUNX1/CASP3/BCL6/JAG1/ACVR1B |
| regulation of epithelial cell proliferation | WNT3A/FBXW7/UHRF1/SIRT1/AKT3/TP63/ZFP36/VEGFA/THBS1/TGFBR3/TGFBR1/ADAM17/STAT5A/STAT3/STAT1/SPARC/SP1/SMO/CXCL12/PTEN/SERPINB5/NRAS/NOTCH2/NOTCH1/NFIB/MYC/LAMC1/JUN/ID1/HOXA5/GLI1/FGFR1/ERBB2/EGFR/CDKN1C/CDKN1B/CDK6/CDC42/BRCA2/BMPR2/CCND1 |
| mitotic cell cycle phase transition | TRIM71/CENPJ/CCNJ/LATS2/ADAMTS1/AURKB/HMGA2/TP53/ADAM17/RDX/RBL1/RAD51C/RAD21/PTEN/PPP3CA/PPP2CA/PLK1/MYC/MUC1/EGFR/E2F3/E2F1/CDKN3/CDKN2A/CDKN1C/CDKN1B/CDKN1A/CDK6/CDK4/CDK2/CDC34/CDC25A/CCNG1/CCNE1/CCND3/CCNA2/BRCA1/BCL2/CCND1/ANXA1/ACVR1B |
| regulation of cell development | WNT3A/FBXW7/DNAJB11/DLL1/DICER1/RAB21/TRIB1/ROCK2/NUMBL/RECK/AXIN2/CXCR4/VEGFA/TP53/SMO/CXCL12/PTEN/RELN/MAP2K1/PPP3CA/POU4F2/PLAG1/NTRK3/NOTCH2/NOTCH1/NF2/MYC/CAPRIN1/LIF/LDLR/KIT/ID1/MTOR/FBN1/EIF4G2/EGR2/EFNA5/E2F1/CDC42/BMPR2/BCL2 |
| neuron death | EGLN3/WNT3A/FBXW7/DDIT4/TBK1/SLC7A11/BACE1/SIRT1/BCL2L11/FADD/TP63/BTG2/MAP3K12/WNT1/TP53/TLR4/SOD2/SNCA/SET/RASA1/PPARA/NR4A2/MSH2/MCL1/MAX/KRAS/JUN/FOS/ERBB3/CSF1/CLU/CDC42/CDC34/CASP7/CASP3/BCL2/AXL/RHOA/FAS/APAF1 |
| mononuclear cell differentiation | DLL1/FOXP1/IKZF1/RASGRP1/MAFB/SOCS5/FADD/SOCS1/WNT1/VEGFA/TRAF6/TP53/TGFBR2/ADAM17/STAT3/SP3/NOTCH2/MYC/MYB/MSH2/KIT/JUN/IL6R/HOXA7/MTOR/ERBB2/CSF1/CEBPG/CDK6/CBFB/RUNX1/PRDM1/BCL6/BCL2/BAK1/AXL/RHOA/ANXA1 |
| reproductive structure development | TPPP3/SIRT1/BCL2L11/ADAMTS1/SGPL1/TP63/WT1/VEGFA/TGFBR1/SP3/PTEN/MAP2K1/PLAG1/SERPINB5/NOTCH2/NOTCH1/MYC/MSH2/LIF/KIT/GLI1/GATA6/ESR1/EGFR/MAPK14/CDX2/CDKN1C/CDKN1B/CASP3/BRCA2/BMPR2/PRDM1/BCL2L2/BCL2/CCND1/BAK1/AXL/ANXA1 |
| response to oxygen levels | EGLN3/DDIT4/SIRT1/ROCK2/CXCR4/VEGFA/HSP90B1/TP53/THBS1/TGFBR3/TGFBR2/ADAM17/SOD2/CXCL12/PTEN/PPARA/POU4F2/PLAU/NR4A2/NOTCH1/MYC/IRAK1/GATA6/MTOR/FOXO1/E2F1/DNMT3A/COL1A1/CDKN1B/CCNA2/CASP3/BNIP3L/BCL2/RHOA/FAS/APAF1 |
| urogenital system development | DLL1/BCL2L11/ADAMTS1/SGPL1/FADD/TP63/WT1/WNT1/VEGFA/TGFBR1/STAT1/SMO/PTEN/PLAG1/SERPINB5/NOTCH2/NOTCH1/NFIA/MYC/LIF/IL6R/ID3/GLI1/FBN1/ESR1/ERBB4/COL4A1/CDKN1C/CDKN1B/PRDM1/BCL2/ASS1/RHOA/APAF1/ANXA1/JAG1 |
| cell cycle G1/S phase transition | TRIM71/CENPJ/FBXW7/LATS2/ADAMTS1/TP53/ADAM17/RDX/RBL1/PTEN/PPP3CA/PPP2CA/MYC/MUC1/GLI1/EGFR/E2F3/E2F1/CDKN3/CDKN2A/CDKN1B/CDKN1A/CDK6/CDK4/CDK2/CDC34/CDC25A/CCNE1/CCND3/CCNA2/BCL2/CCND1/ANXA1/ACVR1B |
| regulation of neuron death | EGLN3/WNT3A/FBXW7/DDIT4/TBK1/SLC7A11/BACE1/SIRT1/BCL2L11/BTG2/MAP3K12/WNT1/TP53/TLR4/SOD2/SNCA/SET/RASA1/PPARA/NR4A2/MSH2/MCL1/KRAS/JUN/FOS/ERBB3/CSF1/CLU/CDC42/CDC34/CASP3/BCL2/AXL/RHOA |
| regulation of leukocyte differentiation | FBXW7/FOXP1/TRIB1/RASGRP1/MAFB/SOCS5/FADD/SOCS1/TRAF6/TLR4/TGFBR2/POU4F2/PIK3R1/NOTCH2/MYC/MYB/LIF/HOXA7/MTOR/FOS/FBN1/ERBB2/CSF1/CREB1/CDK6/CBFB/RUNX1/PRDM1/BCL6/AXL/RHOA/ANXA1 |
| lymphocyte differentiation | DLL1/FOXP1/IKZF1/RASGRP1/MAFB/SOCS5/FADD/SOCS1/WNT1/TP53/TGFBR2/ADAM17/STAT3/SP3/NOTCH2/MYB/MSH2/KIT/IL6R/MTOR/ERBB2/CEBPG/CDK6/CBFB/RUNX1/PRDM1/BCL6/BCL2/BAK1/AXL/RHOA/ANXA1 |
| G1/S transition of mitotic cell cycle | TRIM71/CENPJ/LATS2/ADAMTS1/TP53/ADAM17/RDX/RBL1/PTEN/PPP3CA/PPP2CA/MYC/MUC1/EGFR/E2F3/E2F1/CDKN3/CDKN2A/CDKN1B/CDKN1A/CDK6/CDK4/CDK2/CDC34/CDC25A/CCNE1/CCND3/BCL2/CCND1/ANXA1/ACVR1B |
| homeostasis of number of cells | SLC7A11/IKZF1/BCL2L11/AKT3/MAFB/FADD/ZFP36/VEGFA/TGFBR3/ADAM17/STAT3/STAT1/SP3/SMO/NOTCH1/KRAS/KIT/HSPA1A/HOXA5/CSF1/MAPK14/CEBPG/CDK6/CASP3/BCL6/BCL2/BAK1/AXL/FAS/ANXA1/ACVR1B |
| erythrocyte homeostasis | IKZF1/MAFB/ZFP36/VEGFA/TGFBR3/STAT3/STAT1/SP3/KIT/HSPA1A/HOXA5/MAPK14/CEBPG/CDK6/CASP3/BCL6/AXL/ACVR1B |
| erythrocyte differentiation | IKZF1/MAFB/ZFP36/VEGFA/TGFBR3/STAT3/STAT1/SP3/KIT/HSPA1A/HOXA5/MAPK14/CEBPG/CDK6/CASP3/BCL6/ACVR1B |
| mesoderm development | WNT3A/IKZF1/TBX19/TP63/HMGA2/VEGFA/SMO/PPP2CA/NF2/BMPR2 |
| regulation of protein serine/threonine kinase activity | CCNJ/PDCD4/LATS2/SIRT1/TRIB1/RASGRP1/HIPK3/TCL1A/HMGA2/MAP3K12/VEGFA/TSG101/TRAF6/TLR4/THBS1/ADAM17/SNCA/PTEN/MAP2K1/PPP2CA/PLK1/PAK1/NTRK3/KIT/IRAK1/GTF2H1/FGFR1/ERBB2/EGFR/CDKN3/CDKN2A/CDKN1C/CDKN1B/CDKN1A/CDC25A/CCNG1/CCNE1/CCND3/CCNA2/CASP3/BMPR2/CCND1/RHOA |
| regulation of hemopoiesis | FBXW7/DLL1/FOXP1/TRIB1/RASGRP1/MAFB/SOCS5/FADD/SOCS1/ZFP36/TRAF6/TLR4/TGFBR2/STAT3/STAT1/POU4F2/PIK3R1/NOTCH2/MYC/MYB/LIF/HSPA1A/HOXB8/HOXA7/HOXA5/MTOR/FOS/FBN1/ERBB2/CSF1/MAPK14/CREB1/CDK6/CBFB/RUNX1/PRDM1/BCL6/AXL/RHOA/ANXA1/JAG1/ACVR1B |
| positive regulation of protein kinase activity | WNT3A/FBXW7/SIRT1/RASGRP1/SOCS1/AXIN2/TCL1A/HMGA2/MAP3K12/VEGFA/TRAF6/TLR4/THBS1/TGFBR2/ADAM17/SNCA/RELN/MAP2K1/PPP2CA/PAK1/NTRK3/KIT/IRAK1/IL6R/MTOR/FGFR1/ERBB3/ERBB2/EGFR/EFNA5/CSF1/CLU/CDKN1B/CDKN1A/CCND3/BMPR2/CCND1/RHOA |
| response to hypoxia | EGLN3/DDIT4/SIRT1/ROCK2/CXCR4/VEGFA/HSP90B1/TP53/THBS1/TGFBR3/TGFBR2/ADAM17/SOD2/CXCL12/PTEN/PPARA/PLAU/NR4A2/NOTCH1/MYC/IRAK1/GATA6/MTOR/E2F1/DNMT3A/CDKN1B/CCNA2/CASP3/BNIP3L/BCL2/RHOA/APAF1 |
| response to decreased oxygen levels | EGLN3/DDIT4/SIRT1/ROCK2/CXCR4/VEGFA/HSP90B1/TP53/THBS1/TGFBR3/TGFBR2/ADAM17/SOD2/CXCL12/PTEN/PPARA/PLAU/NR4A2/NOTCH1/MYC/IRAK1/GATA6/MTOR/E2F1/DNMT3A/CDKN1B/CCNA2/CASP3/BNIP3L/BCL2/RHOA/APAF1 |
| intrinsic apoptotic signaling pathway in response to DNA damage | DDIT4/SIRT1/TOPORS/BCL2L11/TP63/TP53/SOD2/CXCL12/PIK3R1/MUC1/MCL1/HNRNPK/E2F1/CLU/CDKN1A/CD44/BRCA2/BRCA1/BCL2L2/BCL2/BAK1 |
| peptidyl-serine phosphorylation | WNT3A/DDIT4/NLK/TBK1/LATS2/HIPK3/AKT3/ROCK2/AURKB/TCL1A/HMGA2/MAP3K12/VEGFA/TGFBR2/TGFBR1/SNCA/RAF1/PTEN/MAPK9/MAPK7/PLK1/PAK1/NTRK3/LIF/MTOR/EGFR/DYRK1A/MAPK14/CREBL2/CDK2/CD44/BCL2/BAK1 |
| muscle cell proliferation | PDCD4/FOXP1/TRIB1/ADAMTS1/TPM1/TLR4/THBS1/TGFBR3/TGFBR2/TGFBR1/STAT1/SOD2/PTEN/MAPK11/NOTCH1/MYC/MEF2D/JUN/JARID2/IRAK1/IL6R/IGFBP3/GLI1/GATA6/ERBB4/DNMT1/MAPK14/CDKN1B/CDKN1A/RUNX1/BMPR2/RHOA |
| neuron apoptotic process | EGLN3/FBXW7/BACE1/BCL2L11/FADD/TP63/BTG2/MAP3K12/TP53/SOD2/SNCA/SET/RASA1/NR4A2/MSH2/MCL1/MAX/KRAS/JUN/ERBB3/CDC42/CDC34/CASP7/CASP3/BCL2/AXL/RHOA/FAS/APAF1 |

**Table S12**. Table of Information about adenoviruses.

| Adenovirus |  |
| --- | --- |
| Species | Rats |
| Vector name | GV407 |
| Element sequence | pAAV-F4/80p-EGFP-MIR155(RNAi)-SV40 PolyA |
| Control insert sequence | TTCTCCGAACGTGTCACGT |
| SiRNA sequence | CCCTATGACAGTGCCAACTAT |

**Table S13**. Table of Information about a lentivirus.

| Lentivirus |  |
| --- | --- |
| Species | Mouse |
| Vector name | Plv-Neo |
| Element sequence | Psi-RRE-Cppt/CTS-PCMV IE-MCS-IRES-Neomycin-WPRE |
| Control insert sequence | TTCTCCGAACGTGTCACGT |
| SiRNA sequence | GCCCTATGACAGTGCCAACTA |

**Table S14**. Table of patch clamp results last 10 s.

|  | ICH+Si-NC | | | | | ICH+Si-Ikzf1 | | | | |
| --- | --- | --- | --- | --- | --- | --- | --- | --- | --- | --- |
| Time (Min) | 1 | 2 | 3 | 4 | 5 | 1 | 2 | 3 | 4 | 5 |
| 80.5 | 107.1964 | 103.8949 | 127.7092 | 119.3281 | 127.8534 | 134.1968 | 127.4112 | 135.0976 | 158.7413 | 138.3790 |
| 81.0 | 114.6830 | 107.2775 | 128.4296 | 120.0218 | 132.2761 | 132.6431 | 134.9306 | 141.5814 | 154.6026 | 129.6750 |
| 81.5 | 111.6938 | 112.9469 | 130.7638 | 119.6119 | 124.7301 | 133.5089 | 127.3158 | 140.8937 | 166.1661 | 131.1172 |
| 82.0 | 111.4263 | 105.5290 | 126.9463 | 123.6840 | 122.2723 | 130.6026 | 142.6582 | 143.2065 | 163.4201 | 126.7861 |
| 82.5 | 113.2531 | 106.1574 | 125.6219 | 121.3232 | 132.2387 | 139.5448 | 133.4579 | 136.6646 | 158.2241 | 127.1067 |
| 83.0 | 113.1274 | 102.3681 | 129.2048 | 117.4178 | 126.5396 | 129.0397 | 143.5363 | 145.1622 | 157.5943 | 123.0369 |
| 83.5 | 109.7336 | 98.0845 | 123.7614 | 127.8626 | 107.9779 | 136.9616 | 126.2092 | 133.8961 | 165.4633 | 125.9345 |
| 84.0 | 115.8419 | 109.0920 | 129.1866 | 126.3292 | 117.8134 | 119.8666 | 122.3223 | 144.4053 | 160.1746 | 121.0675 |
| 84.5 | 107.4408 | 100.2831 | 131.9482 | 127.1953 | 123.5088 | 129.3987 | 122.7641 | 141.4887 | 167.0324 | 123.3582 |
| 85.0 | 104.5886 | 109.9995 | 124.0763 | 123.6945 | 118.6554 | 128.7216 | 134.1044 | 138.1545 | 160.6761 | 125.8896 |
| 85.5 | 113.0104 | 107.9172 | 121.0267 | 122.6695 | 122.5679 | 134.4093 | 130.3971 | 140.0266 | 159.1727 | 127.2677 |
| 86.0 | 110.0803 | 107.8612 | 127.9272 | 122.2294 | 121.6539 | 132.0382 | 137.6209 | 131.4268 | 150.3652 | 126.7236 |
| 86.5 | 107.7496 | 110.3626 | 122.0169 | 121.2306 | 115.6738 | 128.8049 | 127.0517 | 129.5405 | 162.0218 | 124.4888 |
| 87.0 | 108.1209 | 102.2446 | 126.8964 | 125.3355 | 113.7758 | 128.3958 | 128.2986 | 134.9603 | 170.6262 | 120.7866 |
| 87.5 | 105.5672 | 107.1652 | 124.2016 | 128.7869 | 129.9657 | 131.4279 | 117.6468 | 132.8199 | 157.0986 | 137.1978 |
| 88.0 | 107.2282 | 105.8668 | 130.2441 | 128.8250 | 124.6508 | 127.5774 | 123.6358 | 136.2140 | 169.8975 | 134.2008 |
| 88.5 | 112.4820 | 102.5515 | 125.8273 | 124.6785 | 114.8707 | 133.3475 | 127.6173 | 144.1164 | 156.6223 | 131.4774 |
| 89.0 | 109.3283 | 108.7368 | 124.4449 | 119.6748 | 122.1431 | 129.2433 | 124.7131 | 134.9425 | 163.5126 | 136.8551 |
| 89.5 | 117.1419 | 111.1443 | 120.3511 | 128.9343 | 131.3152 | 126.8935 | 136.8284 | 129.7487 | 162.1851 | 131.2929 |
| 90.0 | 110.4902 | 109.0289 | 124.0177 | 123.0435 | 127.6812 | 124.7661 | 128.0414 | 144.2902 | 163.4694 | 141.6536 |
| Mean | 110.5092 | 106.4256 | 126.2301 | 123.5938 | 122.9082 | 130.5694 | 129.8280 | 137.9318 | 161.3533 | 129.2148 |

**Table S15**. Table of key resources.

| **Reagent or Resource** | **Sources** | **IDENTIFIER** |
| --- | --- | --- |
| anti-Bax | CST | #14796 |
| anti-Bcl2 | CST | #3498 |
| anti-Ampk | CST | #9158 |
| anti-p-Ampk | CST | #2535 |
| anti-Ikzf1 | CST | #14859 |
| anti-GSDMD | CST | #39754 |
| anti-cleave-GSDMD | CST | # 36425 |
| anti-Il1b | CST | #12242 |
| anti-cleave-caspase-3 | CST | #9661 |
| anti-S1RT1 | Proteintech | 60303-1-lg |
| anti-Caspase-3 | Proteintech | 66470-2-lg |
| anti-Caspase-9 | Proteintech | 10380-1-AP |
| anti-Caspase-1 | Proteintech | 22915-1-AP |
| anti-ASC | Proteintech | 67494-1-lg |
| anti-PAPR1 | Proteintech | 13371-1-AP |
| anti-NLRP3 | Proteintech | 30109-1-AP |
| anti-mouse | Proteintech | SA00001-7L |
| anti-rorbt | Proteintech | SA00001-4 |
| anti-β-actin | Proteintech | 81115-1-RR |
| anti-Ptn | SANTA | sc-74443 |
| anti-Il18 | abcam | ab191860 |
| anti-Map2 | Invitrogen | PA1-10005 |
| anti-Iba1 | Invitrogen | PA5-143572 |
| anti-chicken | Invitrogen | A-21437 |
| anti-mouse | Invitrogen | A-21200 |
| anti-rorbt | Invitrogen | A-31572 |
| Alexa Fluor® 700 anti-rat CD45 | BioLegend | 202218 |
| FITC anti-rat CD11b/c | BioLegend | 201805 |
| CD11b/c (Microglia) MicroBeads, rat | Miltenyi Biotec | 130-105-634 |
| FlexAble CoraLite® Plus 750 Antibody Labeling Kit for Mouse IgG1 | Proteintech | KFA024 |
| FlexAble CoraLite® Plus 405 Antibody Labeling Kit for Rorbt IgG | Proteintech | KFA006 |
